# Commensal bacteria weaken the intestinal barrier by suppressing epithelial neuropilin-1 and Hedgehog signaling

**DOI:** 10.1038/s42255-023-00828-5

**Published:** 2023-07-06

**Authors:** Giulia Pontarollo, Bettina Kollar, Amrit Mann, My Phung Khuu, Klytaimnistra Kiouptsi, Franziska Bayer, Inês Brandão, Valeriya V. Zinina, Jennifer Hahlbrock, Frano Malinarich, Maximilian Mimmler, Sudhanshu Bhushan, Federico Marini, Wolfram Ruf, Meriem Belheouane, John F. Baines, Kristina Endres, Scott M. Reba, Verena K. Raker, Carsten Deppermann, Christoph Welsch, Markus Bosmann, Natalia Soshnikova, Benoit Chassaing, Mattias Bergentall, Felix Sommer, Fredrik Bäckhed, Christoph Reinhardt

**Affiliations:** 1 Center for Thrombosis and Hemostasis (CTH), University Medical Center Mainz, Johannes Gutenberg-University Mainz, Mainz, Germany; 2 Department of Chemistry, Biochemistry, Johannes Gutenberg-University Mainz, Mainz, Germany; 3 German Center for Cardiovascular Research (DZHK), Partner Site RhineMain, Mainz, Germany; 4 Institute of Molecular Medicine, University Medical Center Mainz, Johannes Gutenberg-University Mainz, Mainz, Germany; 5 Institute of Anatomy and Cell Biology, Unit of Reproductive Biology, Justus-Liebig-University of Giessen, Giessen, Germany; 6 Institute of Medical Biostatistics, Epidemiology and Informatics (IMBEI), University Medical Center Mainz, Johannes Gutenberg-University Mainz, Mainz, Germany; 7 Institute for Experimental Medicine, Kiel University and Max Planck Institute for Evolutionary Biology, Plön, Germany; 8 Department of Psychiatry and Psychotherapy, University Medical Center Mainz, Johannes Gutenberg-University Mainz, Mainz, Germany; 9 Department of Medicine, Case Western Reserve University and University Hospitals Cleveland Medical Center, Cleveland, OH USA; 10 Department of Internal Medicine 1, Goethe University Hospital Frankfurt, Frankfurt am Main, Germany; 11 Pulmonary Center, Department of Medicine, Boston University School of Medicine, Boston, MA USA; 12 INSERM U1016, Team ‘Mucosal microbiota in chronic inflammatory diseases’, CNRS UMR 8104, Université de Paris, Paris, France; 13 Department of Molecular and Clinical Medicine, Wallenberg Laboratory, Institute of Medicine, University of Gothenburg, Gothenburg, Sweden; 14 Institute of Clinical Molecular Biology, Christian-Albrechts-University, Kiel, Germany; 15 Novo Nordisk Foundation Center for Basic Metabolic Research, Section for Metabolic Receptology and Enteroendocrinology, Faculty of Health Sciences, University of Copenhagen, Copenhagen, Denmark; 16 Department of Clinical Physiology, Region Västra Götland, Sahlgrenska University Hospital, Gothenburg, Sweden

**Keywords:** Toll-like receptors, Microbiome, Metabolism, Gastrointestinal system

## Abstract

The gut microbiota influences intestinal barrier integrity through mechanisms that are incompletely understood. Here we show that the commensal microbiota weakens the intestinal barrier by suppressing epithelial neuropilin-1 (NRP1) and Hedgehog (Hh) signaling. Microbial colonization of germ-free mice dampens signaling of the intestinal Hh pathway through epithelial Toll-like receptor (TLR)-2, resulting in decreased epithelial NRP1 protein levels. Following activation via TLR2/TLR6, epithelial NRP1, a positive-feedback regulator of Hh signaling, is lysosomally degraded. Conversely, elevated epithelial NRP1 levels in germ-free mice are associated with a strengthened gut barrier. Functionally, intestinal epithelial cell-specific *Nrp1* deficiency (*Nrp1*^ΔIEC^) results in decreased Hh pathway activity and a weakened gut barrier. In addition, *Nrp1*^ΔIEC^ mice have a reduced density of capillary networks in their small intestinal villus structures. Collectively, our results reveal a role for the commensal microbiota and epithelial NRP1 signaling in the regulation of intestinal barrier function through postnatal control of Hh signaling.

## Abstract (web summary)

A molecular mechanism is revealed through which commensal bacteria modulate intestinal epithelial barrier function.

## Main

At birth, mammals are colonized by microbes from the environment, resulting in the formation of a mutualistic microbial ecosystem, the microbiota^1^. In the gastrointestinal tract, this symbiotic relationship shapes postnatal gut development^2,3^, promotes epithelial cell turnover^4–6^ and regulates the intestinal epithelial barrier^7^.

The gut epithelial barrier consists of a monolayer of terminally differentiated intestinal epithelial cells (IECs) arising from the stem cell niche. Neighboring epithelial cells are sealed by tight and adherens junction protein complexes, whose main role is to prevent paracellular leakage of luminal contents^8,9^. Tight junction complexes consist mainly of claudins, junctional adhesion molecules, zonula occludens-1 (ZO-1) and occludin, whereas adherens junctions are formed by interaction of E-cadherin, α-catenin and β-catenin.

The gut epithelial barrier^10–13^, as well as intestinal epithelial renewal^14–16^, is regulated by pattern recognition receptor signaling pathways and specifically by TLRs. IECs can, to some extent, sense pathogen-associated molecular patterns of gut-resident microbes via TLR signaling^12,16–19^. How pattern recognition of bacterial ligands by the gut epithelium translates into impaired intestinal barrier function remains elusive.

Intestinal growth and differentiation are tightly controlled by feedback-signaling loops that transmit signals between the epithelium and the mesenchyme^20,21^. The epithelial morphogens Indian Hedgehog (IHH) and Sonic Hedgehog (SHH) are engaged in normal gut development^22,23^, but their regulatory role in adult gut physiology is poorly understood. In this intestinal morphogenetic signaling pathway, which signals from the epithelium to the mesenchyme^23^, the Hh precursor proteins IHH and SHH are expressed by terminally differentiated IECs and signal to Patched (PTCH) receptors, expressed by subepithelial myofibroblasts and smooth muscle cells^23–25^. Binding to PTCH, the active Hh ligands alleviate the inhibitory effect on the G-protein coupled receptor Smoothened (SMO)^21^, in turn activating the zinc-finger glioma-associated oncogene transcription factors (GLI), thus restricting uncontrolled epithelial renewal from the stem cell niche via the regulation of Wnt signaling through bone morphogenetic proteins (BMPs)^21,26^.

The type I transmembrane glycoprotein NRP1, an important regulator of developmental tyrosine kinase signaling^27^, has been unveiled as a positive regulator of Hh signal transduction in cell culture models, acting in a positive-feedback circuit^28,29^. Notably, NRP1 expressed by colon adenocarcinoma cells promotes tumor angiogenesis^30^. In endothelial cells, NRP1 is a well-established co-receptor of angiogenic vascular endothelial growth factor receptor-2 (VEGFR-2) signaling and it is a receptor for class 3 semaphorins (SEMA3), influencing both angiogenesis and neuronal axon guidance^31,32^. Although the Hh pathway is pivotal for gut development^21^, it is presently unknown whether this pathway is modulated by epithelial NRP1 and the commensal gut microbiota in the small intestine in vivo and how this affects intestinal physiology at steady-state conditions.

Here, we present a role for microbiota-triggered TLR signaling in the small intestinal epithelium in the postnatal control of the Hh pathway and the regulation of the intestinal barrier. We identified epithelial NRP1 as a critical element that in the absence of gut commensals augments Hh signaling, thus supporting the integrity of the gut epithelial barrier. By colonization with gut microbiota, epithelial cell surface expression of NRP1 was abolished via TLR2 signaling, promoting NRP1 degradation. Unexpectedly, epithelial deficiency of NRP1 was linked to decreased vascularization of small intestinal villus structures. In line with strengthened gut barrier function and increased epithelial NRP1 levels of germ-free (GF) mice, the tissue-specific epithelial deficiency of NRP1 expression resulted in the microbiota-dependent suppression of Hh signaling, linked to a weakened gut epithelial barrier.

## Commensals suppress morphogenetic Hedgehog signals via TLR2

**Fig. 1:**
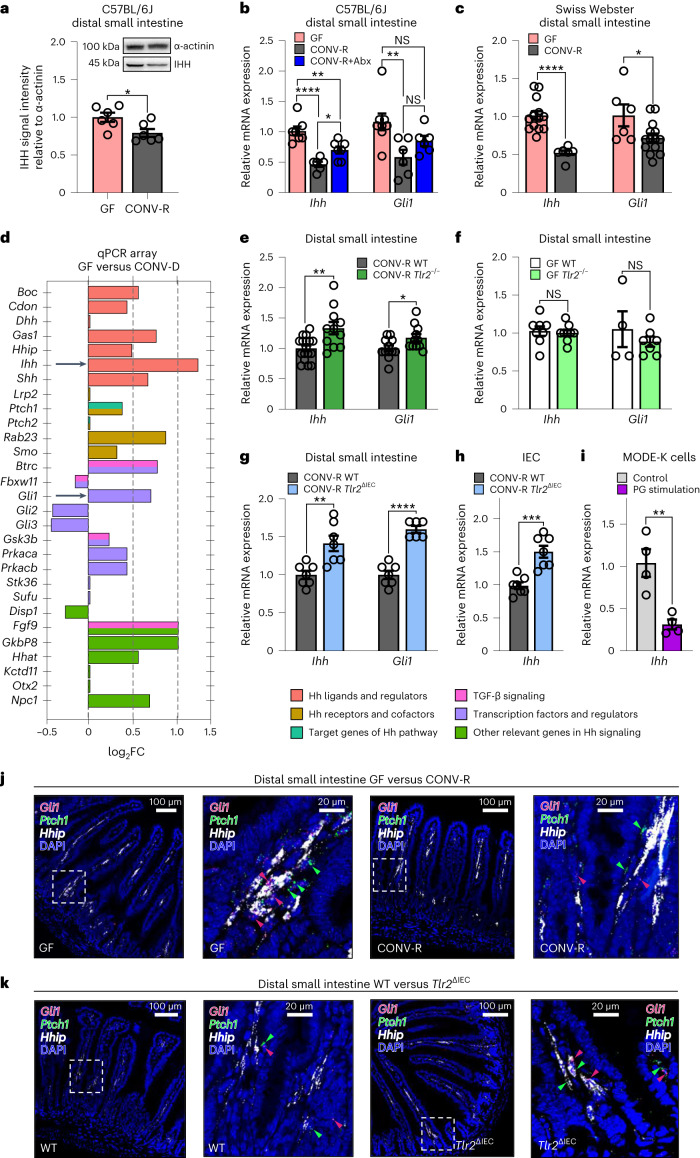
Hedgehog pathway is modulated by the gut microbiota via epithelial TLR2. **a**, Comparative immunoblot analysis of IHH protein levels of small intestinal tissue lysates from GF versus CONV-R C57BL/6J mice, relative to α-actinin (*n* = 6 versus 6, *P* = 0.0262). The 45 kDa IHH precursor is detected. Insert shows a representative western blot. **b**,**c**, Relative gene expression of *Ihh* and *Gli1* in (**b**) C57BL/6J GF, CONV-R and CONV-R mice treated with antibiotics (CONV-R + Abx) (*Ihh*: *n* = 7 versus 6 versus 7; GF versus CONV-R, *P* < 0.0001; GF versus CONV-R + Abx, *P* = 0.0045; CONV-R versus CONV-R + Abx, *P* = 0.0413; *Gli1*: *n* = 8 versus 6 versus 6; GF versus CONV-R, *P* = 0.0097) or (**c**) Swiss Webster GF versus CONV-R mice (*Ihh*: *n* = 14 versus 6, *P* < 0.0001; *Gli1*: *n* = 6 versus 14, *P* = 0.0303). NS, not significant. **d**, qRT–PCR array on pooled concentration-adjusted mRNAs of seven mice per group (*n* = 7 versus 7), showing differential expression of genes involved in the Hh pathway (see legend) between GF and CONV-D mice (relative to CONV-D). *Ihh* and *Gli1* are highlighted with a black arrow. FC, fold change. TGF, transforming growth factor. **e–h**, Relative gene expression of *Ihh* and *Gli1* in WT mice versus *Tlr2*^−/−^ global knockout mice in CONV-R (**e**) (*Ihh*: *n* = 17 versus 11, *P* = 0.0025; *Gli1*: *n* = 12 versus 11, *P* = 0.0298) or GF (**f**) (*Ihh*: *n* = 8 versus 9; *Gli1*: *n* = 4 versus 7) housing conditions and in *Tlr2*^ΔIEC^ CONV-R mice (**g**,**h**) in comparison to WT littermates (distal small intestine: *Ihh*, *n* = 7 versus 7, *P* = 0.0035; *Gli1*: *n* = 7 versus 6, *P* < 0.0001. IECs: *Ihh*, *n* = 7 versus 7, *P* = 0.0003). qRT–PCR analyses were performed on the whole tissue (distal small intestine) (**b–g**), whereas for **h** analyses were on isolated IECs. **i**, Relative *Ihh* expression in MODE-K cells after stimulation with the TLR2 agonist PG (*n* = 4 versus 4, *P* = 0.0059). For qRT–PCR assays, *L32* was used as a housekeeping gene. In all panels, values were normalized for the mean expression of the control group. Individual values are displayed as dots, while mean ± s.e.m. is shown as a column and error bar (**a**–**c**,**e**–**i**). Statistical analyses were performed with one-way analysis of variance (ANOVA) and Tukey’s multiple comparison test (**b**). Unpaired Student’s *t*-test was used (**a**,**c**,**e**–**i**). *P < 0.05, **P < 0.01, ***P < 0.001, ****P < 0.0001. **j**,**k**, sm-FISH for the Hh downstream targets *Gli1* (magenta), *Ptch1* (green) and *Hhip* (white) on distal small intestine sections from GF versus CONV-R (**j**) and *Tlr2*^ΔIEC^ in comparison to WT littermates (**k**). *Gli1* and *Ptch1* transcripts are highlighted with color-coded arrowheads. For each group, the experiment was performed on *n* = 3 mice. For each representative image, two magnifications are shown. Scale bars, 100 μm and 20 μm. DAPI, 4,6-diamidino-2-phenylindole. *n* represents the number of biological independent mice (**a**–**c**,**e**–**h**,**j**), whereas in **i** it represents the number of independent experiments on cell cultures. Source data

**Extended Data Fig. 1:**
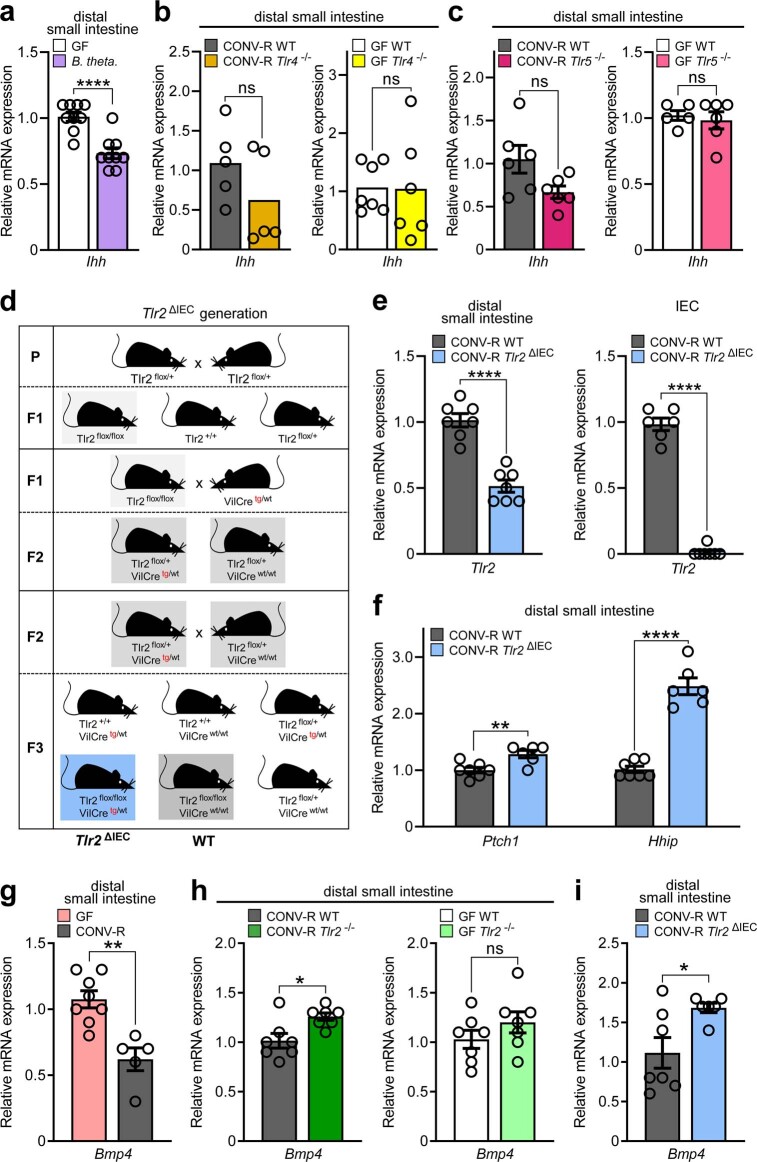
Indian Hedgehog expression in monocolonized and TLR-knockout mouse models; generation and characterization of conditional intestinal epithelial *Tlr2* knockout mouse model; bone morphogenetic protein 4 regulation by gut microbiota through TLR2. **(a)** Relative mRNA expression of *Ihh* in the distal small intestine of GF mice compared to ex-GF mice monocolonized with *Bacteroides thetaiotaomicron* (that is *B. theta*.) (*n* = 9 versus 9,  *P* < 0.0001). **(b, c)** Relative gene expression of *Ihh* in the distal small intestine of WT mice versus (**b**) *Tlr4*^*−/−*^ (CONV-R: *n* = 5 versus 5; GF, *n* = 7 versus 6) and (**c**) *Tlr5*^*−/−*^ (CONV-R: *n* = 6 versus 6; GF, *n* = 5 versus 6) global knockout mice in CONV-R and GF housing conditions. (**d**) Generation of the conditional intestinal epithelial *Tlr2*-deficient mouse line (*Tlr2*^ΔIEC^). Mice used in the breeding pairs are highlighted by colored squares. P: parental generation; F1, F2, F3: first, second, third filial generation. **(e)** Relative mRNA expression of *Tlr2* in the distal small intestine (*n* = 7 versus 7, *P* < 0.0001) or IEC (*n* = 6 versus 7, *P* < 0.0001) from WT littermates versus *Tlr2*^ΔIEC^. **(f)** Relative mRNA expression of *Ptch1* and *Hhip* in the distal small intestine of WT littermates versus *Tlr2*^ΔIEC^ mice (*n* = 7 versus 6, *Ptch1:* *P* = 0.0047; *Hhip:* *P* < 0.0001). (**g-i**) Relative mRNA expression of the Hh target bone morphogenetic protein 4 (*Bmp4*) in the distal small intestine of (**g**) GF versus CONV-R mice (*n* = 8 versus 5, *P* = 0.0013), (**h**) WT mice versus *Tlr2*^*−/−*^ global knockout mice in CONV-R (*n* = 7 versus 7, *P* = 0.0123) and GF (*n* = 7 versus 7) housing conditions and (**i**) WT littermates versus *Tlr2*^ΔIEC^ mice (*n* = 7 versus 6, *P* = 0.0238). For panels **a-c**, **e–i**, *n* represents the number of biological independent mice. For the qPCR assays, *L32* was used as the housekeeping gene and the values were normalized for the mean expression of the control group. For panels **a-c**, **e–i**, Individual values are shown as dots, while mean ± s.e.m. is shown as a column and error bar (except for panel **b**, due to bimodal distribution) and unpaired Student’s *t* test was used. *P < 0.05, **P < 0.01, ****P < 0.0001. Source data

The gut microbiota exerts dramatic changes on small intestinal morphology^2–5^, but the microbiota-triggered signaling mechanisms affecting intestinal homeostasis remain elusive. Therefore, we analyzed whether the gut microbiota has an impact on the Hh pathway, a major pathway involved in gut development^21–25^. IHH protein is expressed by terminally differentiated enterocytes, whereas *Ihh* messenger RNA expression is highest in the crypt villus junctions^33^. Of note, western blot analyses on small intestinal tissue lysates of adult mice revealed that the major Hh morphogen IHH is elevated on the protein level at GF housing conditions as compared to conventionally raised (CONV-R) counterparts (Fig. 1a). Both in C57BL/6J and Swiss Webster mice, transcript levels of *Ihh* were markedly reduced in the small intestine of CONV-R mice relative to GF housing conditions (Fig. 1b,c). The influence of gut commensals on Hh signaling was further corroborated by reduced expression of the Hh target gene *Gli1* in CONV-R mice as compared to GF controls (Fig. 1b,c). As a confirmation, antibiotic-induced decimation of commensals substantially increased Hh transcripts and reversed the microbiota-dependent decrease of Hh signaling, reflecting the reversible and dynamic microbiota-dependent regulation of this morphogenetic signaling pathway (Fig. 1b). In line with these findings, monocolonization of GF mice with the common gut symbiont *Bacteroides* *thetaiotaomicron* was sufficient to dampen small intestinal *Ihh* expression (Extended Data Fig. 1a). As the presence of gut microbiota consistently attenuated the Hh signaling axis, we next performed a qPCR array analysis comparing GF mice to conventionalized mice (conventionally derived (CONV-D) GF mice colonized with the cecal microbiota from a CONV-R mouse for 2 weeks). This analysis confirmed suppression of various elements of the Hh pathway by colonization with gut microbiota, including *Ihh* and *Gli1* (Fig. 1d). Collectively, our results demonstrate a dynamic microbiota-triggered suppression of the intestinal Hh pathway.

This finding prompted us to determine by which regulatory pathway the colonization with gut commensals might trigger the suppression of Hh signaling. As GF mouse models and antibiotics-induced microbiota-depletion have revealed that the expression levels of epithelial TLRs in the ileum, in particular TLR2, TLR4 and TLR5, are tightly controlled by the gut microbiota^15,34^, we next investigated whether microbiota-induced TLR signaling could be involved in dampening intestinal Hh gradients. To this end, we re-derived global *Tlr2*-deficient (*Tlr2*^−/−^), *Tlr4*-deficient (*Tlr4*^−/−^) and *Tlr5*-deficient (*Tlr5*^−/−^) mice as GF and compared the small intestinal Hh signals with their CONV-R counterparts. We observed that CONV-R *Tlr2*^−/−^ mice exhibited increased expression of *Ihh* and *Gli1* compared to CONV-R wild-type (WT) controls, whereas the expression levels did not differ in GF *Tlr2*^−/−^ or GF WT mice (Fig. 1e,f). In contrast to *Tlr2*^−/−^ mice, the *Ihh* expression levels in the distal small intestine of *Tlr4*^−/−^ and *Tlr5*^−/−^ mice were not significantly changed by the presence of gut microbiota (Extended Data Fig. 1b,c). Thus, our results indicate that small intestinal Hh signals are primarily suppressed through microbiota-triggered TLR2 signaling.

To pinpoint whether TLR2 signaling in the gut epithelial compartment is sufficient to elicit suppression of Hh signaling, as observed in the small intestine of the global *Tlr2* knockout mouse model, we generated a *Tlr2-flox* x *Villin-Cre* mouse line (*Tlr2*^ΔIEC^) with markedly reduced *Tlr2* expression in the small intestine and a complete absence of *Tlr2* mRNA in IECs (Extended Data Fig. 1d,e)^35^. In support of our hypothesis that epithelial TLR2 regulates Hh signaling, *Ihh* expression levels were significantly increased in the small intestine and in isolated epithelial cells of tissue-specific *Tlr2*^ΔIEC^ mice relative to the *Cre*-negative *Tlr2*^fl/fl^ littermate controls, whereas transcript levels of the Hh target *Gli1* were elevated in the small intestine (Fig. 1g,h). In addition, elevated expression levels of the Hh target genes *Patched-1* (*Ptch1*) and *Hh interacting protein* (*Hhip*) further support the suppressive role of epithelial TLR2 signaling (Extended Data Fig. 1f). Conversely, stimulation of the mouse small IEC line MODE-K^36^ with the TLR2/TLR6 agonist peptidoglycan (PG) led to a marked reduction of *Ihh* transcript levels (Fig. 1i). Coherent with epithelial-to-mesenchymal signaling of the identified microbiota TLR2-regulated Hh pathway, single-molecule fluorescence in situ hybridization (sm-FISH), comparing GF to CONV-R and *Tlr2*^ΔIEC^ mice with WT-floxed littermates, showed that Hh targets *Gli1*, *Ptch1* and *Hhip* were exclusively expressed in the intravillus mesenchyme (Fig. 1j,k)^22,23^. Underscoring epithelial-to-mesenchymal signaling of the identified signaling axis^37,38^, the mRNA expression of the Hh target gene *Bmp4* (ref. ^23^), which is specifically expressed in the intravillus mesenchyme^39,40^, was likewise suppressed via microbiota-triggered epithelial TLR2 signaling (Extended Data Fig. 1g–i). Collectively, our results reveal TLR2 signaling in the intestinal epithelium as a major signaling hub, connecting the colonization with gut commensals with the adaptation of epithelial Hh signaling gradients.

## Gut epithelial NRP1 is suppressed by microbiota-driven TLR2

Based on cell culture models, NRP1 has been proposed as a central positive-feedback regulator of the Hh pathway, promoting Hh signaling via a 12-amino-acid region in the cytoplasmic domain of the receptor^27–29^. This prompted us to analyze whether the gut microbiota impacts expression levels of the Hh pathway regulator NRP1 in the epithelial lining of the distal small intestine in vivo.

**Fig. 2:**
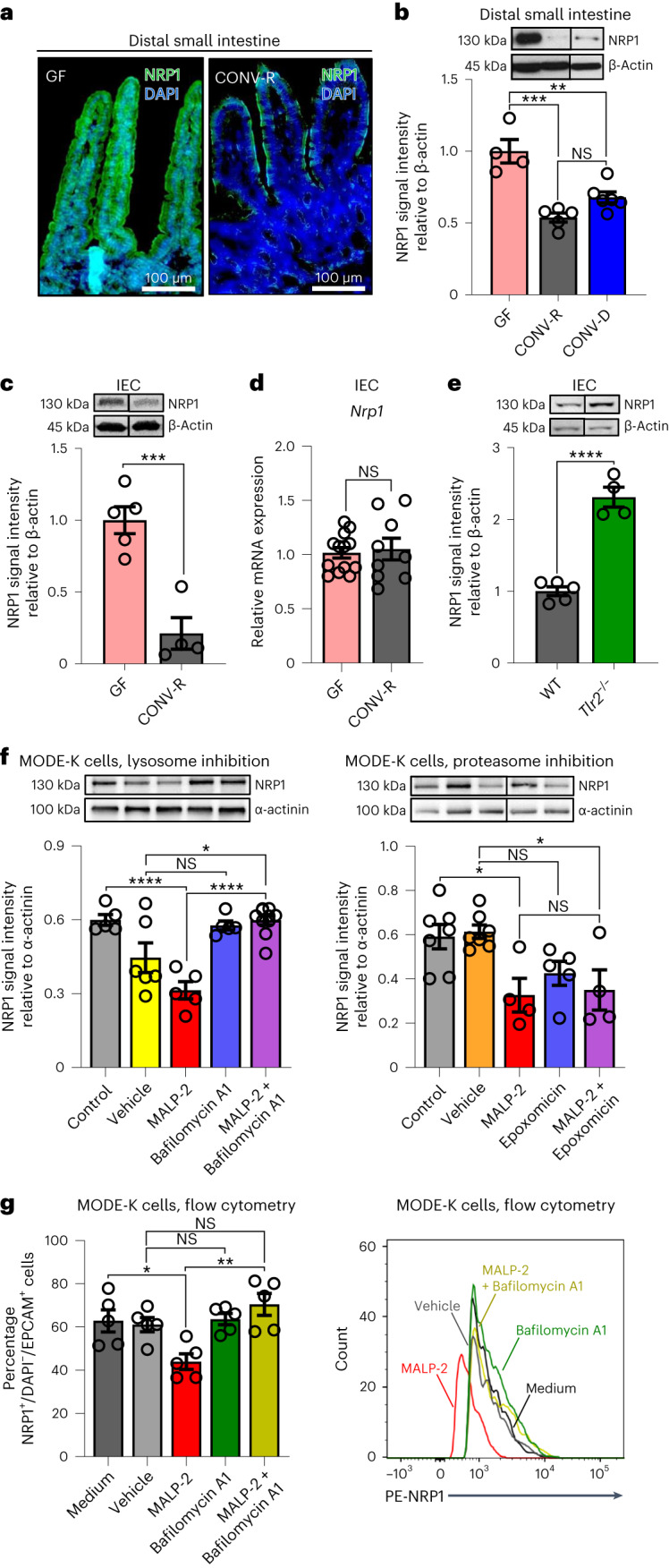
NRP1 protein levels in the gut epithelium are regulated by the gut microbiota through TLR2-mediated lysosomal degradation. **a**, Representative immunofluorescence images of NRP1 expression (green) in the distal small intestine of GF and CONV-R mice. The experiment was performed twice. Cell nuclei are counterstained with DAPI (in blue). Scale bars, 100 μm. **b**, Relative NRP1 protein levels in the distal small intestine of GF, CONV-R and CONV-D mice (*n* = 4 versus 5 versus 6; GF versus CONV-D, *P* = 0.0018; GF versus CONV-R, *P* = 0.0001). **c**–**e**, NRP1 (**c**) protein (*n* = 5 versus 4, *P* = 0.0009) and mRNA (*n* = 13 versus 9) expression (**d**) in IECs isolated from GF and CONV-R mice or WT mice (**e**) versus *Tlr2*^−/−^ in CONV-R housing conditions (*n* = 5 versus 4, *P* < 0.0001). *n* represents the number of biological independent mice (**b**–**e**). **f**, TLR2-mediated NRP1 degradation in MODE-K cells by lysosome or proteasome. NRP1 degradation by TLR2 is induced by MALP-2 (TLR2/TLR6 agonist) stimulation. Blocking of lysosomal degradation (left) is achieved by stimulation with bafilomycin A1 in 0.125% (v/v) DMSO (vehicle). Lysosome inhibition prevents TLR2/TLR6-induced NRP1 degradation (MALP-2 + bafilomycin A1) (control versus MALP-2, *P* < 0.0001; MALP-2 versus MALP-2 + bafilomycin A1, *P* < 0.0001; vehicle versus MALP-2 + bafilomycin A1, *P* = 0.0122). Blocking of proteasome (right) is performed by stimulation with epoxomicin in 0.1% (v/v) DMSO (vehicle). Proteasome inhibition does not prevent NRP1 degradation by MALP-2 (MALP-2 + epoxomicin) (control versus MALP-2, *P* = 0.0328; vehicle versus MALP-2 + epoxomicin, *P* = 0.0340). **g**, Inhibition of NRP1 degradation by lysosome shown by flow cytometry, using the same experimental conditions of **f** (medium versus MALP-2, *P* = 0.0459; MALP-2 versus MALP-2 + bafilomycin A1, *P* = 0.0011). Representative histograms are shown (right). For western blot analyses, the number of independent experiments (*n*) on cell cultures is 4–8, whereas for flow cytometry this was *n* = 5. GF and CONV-R mice were analyzed on different gels that were processed in parallel (**c**). In the qPCR assay, *L32* was used as the housekeeping gene, whereas in western blot, protein expression is relative to α-actinin or β-actin. Values are normalized for the mean expression of the controls (**b**–**f**). Individual values are displayed as dots, whereas mean ± s.e.m. is shown as a column and error bar (**b**–**g**). Statistical analyses were performed with one-way ANOVA and Tukey’s multiple comparison test (**b**,**f**,**g**), whereas for **c**–**e**, an unpaired Student’s *t*-test was used. *P < 0.05, **P < 0.01, ***P < 0.001, ****P < 0.0001. Source data

**Extended Data Fig. 2:**
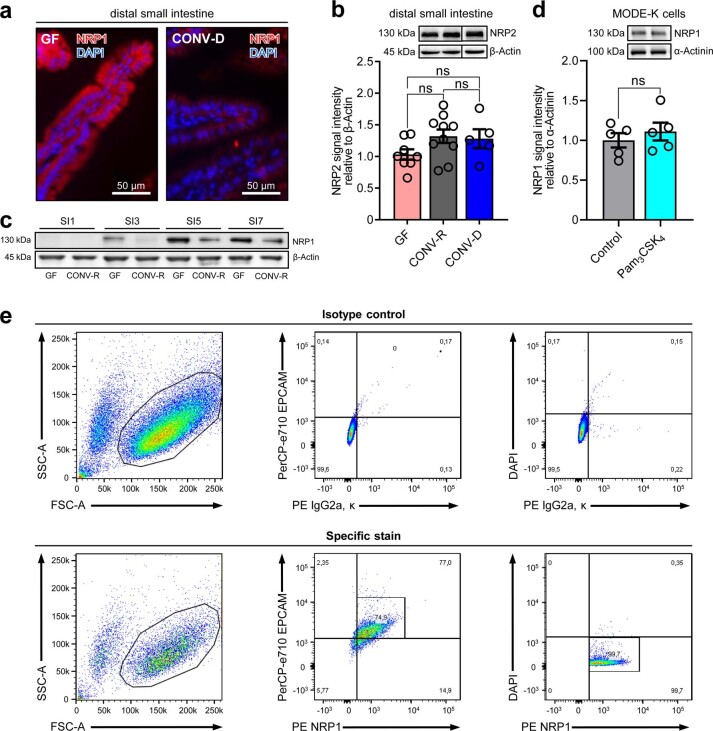
NRP1 and NRP2 expression in the gut of GF, CONV-D and CONV-R mice and MODE-K cell culture model. **(a)** Representative immunofluorescence images of NRP1 expression (in red) in the distal small intestine of GF and CONV-D mice. Cell nuclei are counterstained with DAPI (in blue). Scale bar: 50 μm. The measurements were repeated one time. **(b)** NRP2 expression in the distal small intestine of GF, CONV-R and CONV-D mice (*n* = 8 versus 10 versus 5). **(c)** NRP1 expression along the small intestine of GF mice, compared to CONV-R controls (*n* = 1 versus 1). From the proximal to the distal tract, the small intestine (SI) is divided into 8 equally sized segments. For WB analysis, segments 1, 3, 5, 7 were analyzed. (**d**) Protein expression of NRP1 after stimulation of MODE-K cells with the TLR2/1 agonist Pam_3_CSK_4_ (*n* = 5 versus 5). For panels **b-c**, n represents the number of biological independent mice, whereas for panel **d**, *n* is the number of independent experiments performed on cell cultures. For the western blot assays, protein expression is relative to α-actinin or β-actin and the values were normalized for the mean expression of the control group. For panels **b**, **d**, individual values are shown as dots, whereas mean ± s.e.m. is shown as a column and error bar. For panel **b**, statistical analyses were performed with one-way ANOVA, Tukey’s multiple comparison test. For panel **d**, Unpaired Student’s t test was used. ns: *P* > 0.05. (**e**) Gating strategy for flow cytometry analysis on MODE-K cells. In the FSC-A versus SSC-A panel, single cells are selected. Gating of EPCAM^+^ NRP1^+^ DAPI^−^ singlet specific stain (that is, living IEC expressing NRP1) are based on the isotype control (upper panel). Source data

Immunofluorescence staining of fixed-frozen small intestine sections showed high NRP1 protein expression in terminally differentiated epithelial cells of GF mice compared to CONV-R controls, indicating the microbiota-dependent regulation of this receptor protein (Fig. 2a). Epithelial NRP1 protein levels were likewise reduced in colonized mice (CONV-D) (Extended Data Fig. 2a). Immunoblot analyses confirmed reduced NRP1 protein levels in the distal small intestine of colonized mice, showing a significant reduction of NRP1 in CONV-R and CONV-D mice as compared to GF controls (Fig. 2b). In contrast to NRP1, protein levels of the homolog NRP2 in the distal small intestine were not microbiota-regulated (Extended Data Fig. 2b). Of note, a reduction in NRP1 protein levels in CONV-R as compared to GF housing conditions was consistently found along the entire length of the small intestine, from jejunum to ileum (Extended Data Fig. 2c). This pronounced reduction in NRP1 protein levels in colonized mice was further corroborated with isolated small IECs^41^, demonstrating that the presence of microbiota-suppressed epithelial NRP1 protein expression without affecting transcript levels (Fig. 2c,d). In conclusion, our results show that colonization with gut microbiota yields in reduced protein levels of the Hh regulator NRP1 in primary small intestinal epithelium by post-transcriptional mechanisms.

In accordance with the microbiota-induced suppression of the Hh pathway (Fig. 1a–f), the reduced epithelial protein levels of the Hh pathway regulator NRP1 are a result of the recognition of microbial patterns by TLR2, as NRP1 protein levels were strongly increased in primary small IECs of *Tlr2*^−/−^ mice (Fig. 2e). In line, the diacyl lipopeptide TLR2/TLR6 agonist macrophage-activating lipopeptide-2 (MALP-2) efficiently suppressed NRP1 protein levels in the epithelial MODE-K cell culture model, as demonstrated by western blot and flow cytometry analyses (Fig. 2f,g). In contrast, stimulation of MODE-K cells with the TLR2/TLR1-specific agonist Pam_3_CSK_4_, a synthetic triacylated lipopeptide, did not change NRP1 protein levels (Extended Data Fig. 2d). Next, we interrogated the mechanism by which NRP1 is degraded. Inhibitor treatments revealed that NRP1 is downregulated by a TLR2/TLR6-induced lysosomal pathway. MODE-K cells that were stimulated with MALP-2 following pre-incubation with the lysosomal inhibitor bafilomycin A1, did not show reduced NRP1 protein levels (Fig. 2f). In contrast, the MALP-2-induced reduction in NRP1 protein levels was not prevented by blockade of proteasomal degradation with the inhibitor epoxomicin (Fig. 2f). Of note, the inhibitor experiments with bafilomycin A1 on MODE-K cells were independently confirmed by flow cytometry analysis, demonstrating the blockade of MALP-2-triggered reduction of NRP1 cell surface levels via the lysosomal pathway (Fig. 2g and Extended Data Fig. 2e). Collectively, our analyses revealed that NRP1 in the small intestinal epithelium is downregulated by the commensal microbiota and TLR2 signaling through the lysosomal degradation pathway.

## NRP1 is critical for microbiota-dependent gut barrier control

**Fig. 3:**
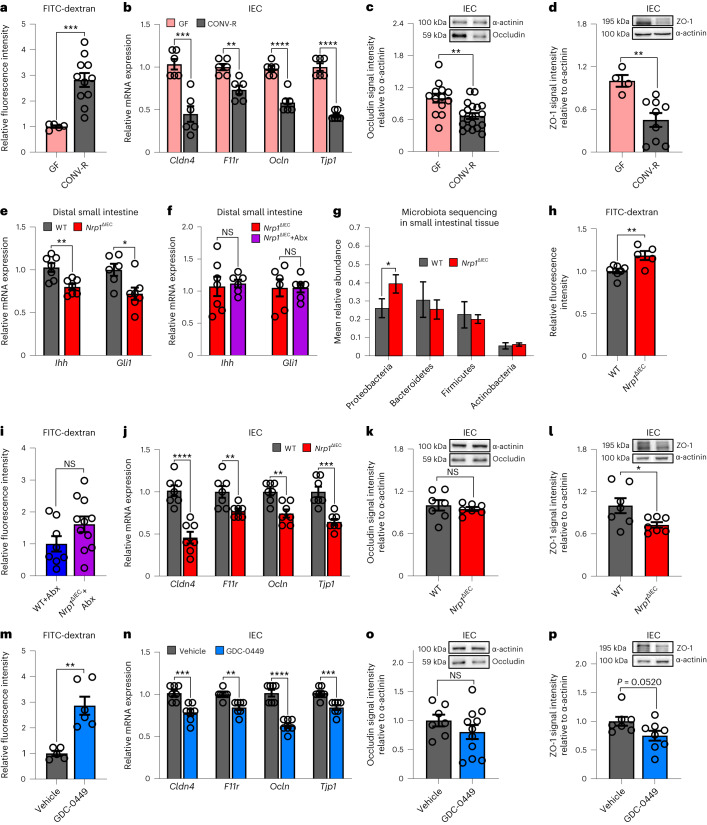
Impairment of gut barrier function by the gut microbiota, gut epithelial NRP1-deficiency and inhibition of Hedgehog signaling. **a**, FITC-dextran permeability assay on GF versus CONV-R mice (*n* = 5 versus 11, *P* = 0.0006). **b**, Relative gene expression of claudin-4 (*Cldn4*), junctional adhesion molecule-A (*F11r*), occludin (*Ocln*) and ZO-1 (*Tjp1*) in IECs from GF versus CONV-R mice (*n* = 6 versus 6; *Cldn4*: *P* = 0.0004; *F11r*: *P* = 0.0015; *Ocln*: *P* < 0.0001; *Tlp1*: *P* < 0.0001). **c**,**d**, Relative occludin (**c**) and ZO-1 (**d**) protein expression in IECs of GF versus CONV-R mice (occludin: *n* = 13 versus 19, *P* = 0.0015; ZO-1: *n* = 4 versus 9, *P* = 0.0053). **e**,**f**, Relative gene expression of *Ihh* and *Gli1* in the distal small intestine of WT littermates versus *Nrp1*^ΔIEC^ mice in CONV-R conditions (**e**) (*Ihh*: *n* = 7 versus 7, *P* = 0.0033; *Gli1*: *n* = 6 versus 7, *P* = 0.0185) and after antibiotic treatment (Abx) (**f**) (*Ihh*: *n* = 7 versus 6; *Gli1*: *n* = 6 versus 6). **g**, Gut microbiota mean relative abundance on the phylum level as determined by bacterial 16S rRNA gene sequencing of small intestinal tissue samples from *Nrp1*^ΔIEC^ mice versus *Cre*-negative WT littermates. **h**,**i**, FITC-dextran permeability assay on WT (*Cre*-negative) littermates versus *Nrp1*^ΔIEC^ in CONV-R status (*n* = 7 vs 5, *P* = 0.0074) (**h**) and after antibiotics treatment (Abx) (*n* = 8 versus 11) (**i**). **j**, Relative gene expression of *Cldn4*, *F11r*, *Ocln*, and *Tjp1* in IEC from WT littermates versus *Nrp1*^ΔIEC^ mice (*n* = 7 versus 7; *Cldn4*: *P* < 0.0001; *F11r*: *P* = 0.0099; *Ocln*: *P* = 0.0019; *Tlp1*: *P* = 0.0003). **k**,**l**, Relative occludin (**k**) and ZO-1 (**l**) protein expression in IEC from WT littermates versus *Nrp1*^ΔIEC^ mice (*n* = 7 versus 7; ZO-1: *P* = 0.0337). **m**, FITC-dextran permeability assay on vehicle controls versus GDC-0449-treated mice (*n* = 5 versus 6, *P* = 0.0012). **n**, Relative gene expression of *Cldn4*, *F11r*, *Ocln*, and *Tjp1* in IEC from controls versus GDC-0449-treated mice (*n* = 7 versus 7; *Cldn4*: *P* = 0.0010; *F11r*: *P* = 0.0011; *Ocln*: *P* < 0.0001; *Tjp1*: *P* = 0.0010). **o**,**p**, Relative occludin (**o**) and ZO-1 (**p**) protein expression in IEC from controls versus GDC-0449-treated mice (occludin: *n* = 7 versus 11; ZO-1: *n* = 7 versus 8). *n* represents the number of biologically independent mice. For the qPCR assays, *L32* was used as the housekeeping gene, whereas in western blots, protein expression is relative to α-actinin. Values were normalized for the mean of the control group. Individual values are displayed as dots, while mean ± s.e.m. is shown as a column and error bar (**a**–**f**,**h**–**p**). Individual values are not shown (**g**). For all panels, unpaired Student’s *t*-test was used. *P < 0.05, **P < 0.01, ***P < 0.001, ****P < 0.0001. Source data

The commensal gut microbiota is a key factor in the regulation of the intestinal epithelial barrier, which substantially depends on epithelial tight junction complexes^7^. By intragastric administration of FITC-dextran to GF and CONV-R mice, we demonstrated that CONV-R mice have reduced paracellular intestinal epithelial barrier function compared to their GF counterparts, with three times lower fluorescence intensity detected in the serum of GF mice (Fig. 3a). Enhanced gut barrier at GF housing conditions was further supported by increased mRNA expression of the tight junction proteins claudin-4 *(Cldn4*), junctional adhesion molecule-A (*F11r*), occludin (*Ocln*) and ZO-1 (tight junction protein-1, *Tjp1*) in isolated small IECs (Fig. 3b). In line with this, the protein levels of occludin and ZO-1 were diminished in the IECs of CONV-R mice relative to GF counterparts (Fig. 3c,d). In accordance with previous reports^7^, our results highlight that the gut microbiota weakens the gut epithelial barrier.

**Extended Data Fig. 3:**
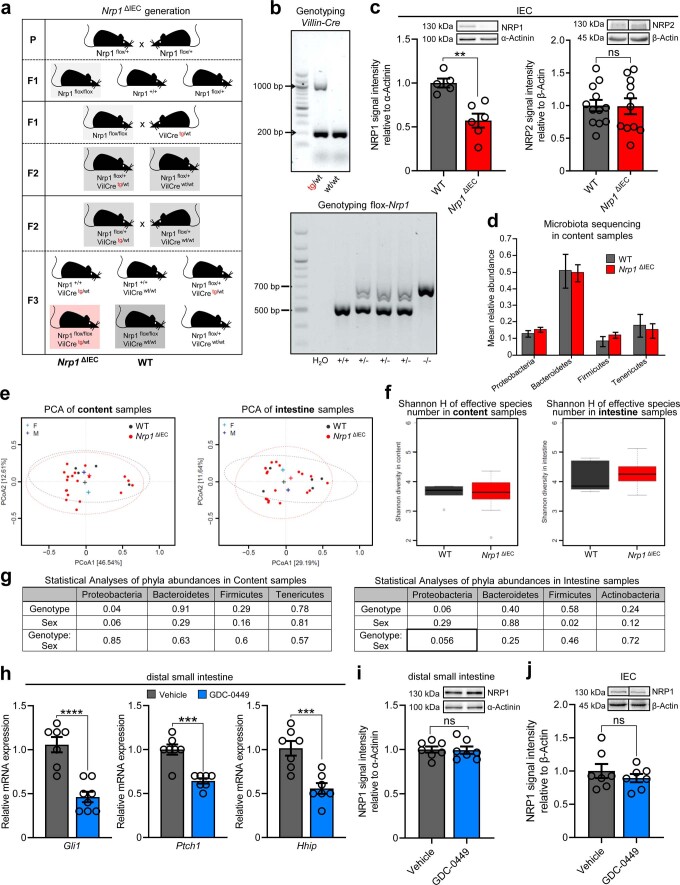
Characterization of the *Nrp1*^ΔIEC^ mouse model, efficacy of NRP1 deficiency, NPR2 expression and microbiome analysis. **(a)** Generation of the *Nrp1*^ΔIEC^ mouse line. Mice used in the breeding pairs are highlighted by colored squares. P: parental generation; F1, F2, F3: first, second, third filial generation. **(b)** Genotyping of *Villin-Cre* (upper panel) and *Nrp1 fl/fl* (lower panel). **(c)** NRP1 (*n* = 5 versus 6, *P* = 0.0019) and NRP2 (*n* = 12 versus 11) protein expression in IEC from WT littermates versus *Nrp1*^ΔIEC^ mice. **(d)** Mean relative abundance on the phylum level as determined by bacterial 16 S rRNA gene sequencing of intestinal content samples from *Nrp1*^ΔIEC^ mice and floxed WT littermates. **(e)** Principal coordinate analysis (PCoA) based on the Bray–Curtis index and **(f)** α-Diversity (Shannon index) of content samples and of intestine samples of 16 S rRNA gene microbiome sequencing from small intestine of *Nrp1*^ΔIEC^ mice versus floxed WT littermates. **(g)** Descriptive statistics (*P* values) of the bacterial 16 S rRNA gene sequencing analysis on *Nrp1*^ΔIEC^ mice versus floxed WT littermates. Differences in Proteobacteria in intestine samples are highlighted by a black box. **(h)** Relative gene expression of *Gli1*, *Ptch1* and *Hhip* in the distal small intestine of vehicle controls compared to GDC-0449-treated mice (*Gli1:* *n* = 7 versus 8, *P* < 0.0001; *Ptch1:* *n* = 7 versus 7, *P* = 0.0001; *Hhip:* *n* = 7 versus 7, *P* = 0.0008). **(i,j)** NRP1 expression in **(i)** the distal small intestine (*n* = 7 versus 7) and **(j)** isolated IEC (*n* = 7 versus 7) from vehicle controls compared to GDC-0449-treated mice. For panels **c**, **h-j**, n represents the number of biological independent mice. For the qPCR assays, *L32* was used as the housekeeping gene, whereas in western blot, protein expression is relative to α-actinin or β-actin. In panels **c** and **h-j** the values were normalized for the mean expression of the control group. For panels **c**, **h-j**, individual values are shown as dots, while mean ± s.e.m. is shown as a column and error bar. For panel **d-g**, individual values are not shown. For panel **c**, **h-j**, unpaired Student’s *t* test was used. **P < 0.01, ***P < 0.001, ****P < 0.0001. Source data

To explore functionally whether the increased intestinal permeability in CONV-R mice could be attributed to the microbiota-triggered reduction in NRP1 in the gut epithelial lining (Fig. 2a–c), we next generated a mouse line with enterocyte-specific *Nrp1* deficiency (*Nrp1*^ΔIEC^) (Extended Data Fig. 3a,b). As expected, deficiency of intestinal epithelial NRP1 did not affect NRP2 protein levels (Extended Data Fig. 3c)^28^. This model enabled us to test whether the microbiota-induced impairment of epithelial NRP1 protein levels may be involved in the weakened intestinal epithelial barrier. In accordance with the microbiota-dependent regulation of the Hh pathway (Fig. 1a–e), *Nrp1*^ΔIEC^ mice displayed significantly reduced small intestinal mRNA levels of *Ihh* and the Hh target *Gli1* but showed unaltered *Ihh* and *Gli1* transcripts when the gut microbiota was depleted by treatment with an antibiotic cocktail containing ampicillin and neomycin (Fig. 3e,f). In line with reduced epithelial NRP1 protein levels (Fig. 2a–c) and increased gut permeability due to reduced expression of tight junctional components in CONV-R mice (Fig. 3a–d), *Nrp1*^ΔIEC^ mice indeed showed impaired intestinal barrier function, as determined by increased FITC-dextran levels in the serum (Fig. 3h). Antibiotic treatment abolished these differences in gut epithelial barrier function (Fig. 3i). The barrier defect observed in *Nrp1*^ΔIEC^ mice was further substantiated by consistently reduced epithelial *Cldn4*, *F11r*, *Ocln* and *Tjp1* transcripts and significantly reduced ZO-1 protein levels, whereas occludin was unaffected (Fig. 3j–l). Notably, the impaired gut barrier function of *Nrp1*^ΔIEC^ mice was associated with an increased abundance of Proteobacteria in small intestinal tissue (Fig. 3g), an association also observed in Crohn’s disease^42^. This difference was not significant in the small intestinal content (Extended Data Fig. 3d). Moreover, principal-component analysis and the Shannon index did not show major differences between the two groups (Extended Data Fig. 3e–g). Thus, our results identify epithelial NRP1 as a regulator of microbiota-influenced gut barrier function, associated with an altered gut microbial diversity.

## Hedgehog suppression weakens the gut epithelial barrier

Since the gut microbiota suppresses Hh pathway activity via lysosomal degradation of the Hh regulator NRP1 in the intestinal epithelium (Figs. 1a–c and 2a–c), we next addressed whether the Hh pathway is involved in the regulation of gut barrier function. As mice with a targeted deletion of *Ihh* were previously shown to be embryonically lethal^25^, we opted for a pharmacological inhibition of the pathway by in vivo treatment of C57BL/6J mice with the Smoothened (Smo)-antagonist GDC-0449 (Vismodegib)^43^. In line with the microbiota-dependent reduction in Hh signaling (Fig. 1a–f and 3e,f) and impaired gut barrier function observed in *Nrp1*^ΔIEC^ mice (Fig. 3h,j,l), the inhibition of tonic Hh signaling with GDC-0449 strongly weakened the epithelial gut barrier (Fig. 3m). The efficacy of the pharmacological in vivo inhibition by GDC-0449 was confirmed by reduced small intestinal transcript levels of the Hh targets *Gli1*, *Ptch1* and *Hhip* (Extended Data Fig. 3h). Of note, GDC-0449 inhibition did not feed back to reduce NRP1 protein levels, neither in distal small intestinal tissue nor in isolated epithelial cells (Extended Data Fig. 3i,j). The GDC-0449 inhibitor treatment demonstrated that impaired gut barrier function caused by suppression of the Hh pathway is due to reduced gut epithelial mRNA expression of several epithelial junction constituents (Fig. 3n). In accordance with the gut epithelial deficiency of the positive Hh regulator NRP1, when the Hh signaling pathway was blocked, epithelial protein levels of the tight junction components occludin and ZO-1 were reduced (Fig. 3o,p). In summary, our results define Hh pathway regulation through microbiota-suppressed epithelial NRP1 as a critical epithelial permeability-regulating factor.

## Epithelial NRP1 deficiency impairs capillary network formation

**Fig. 4:**
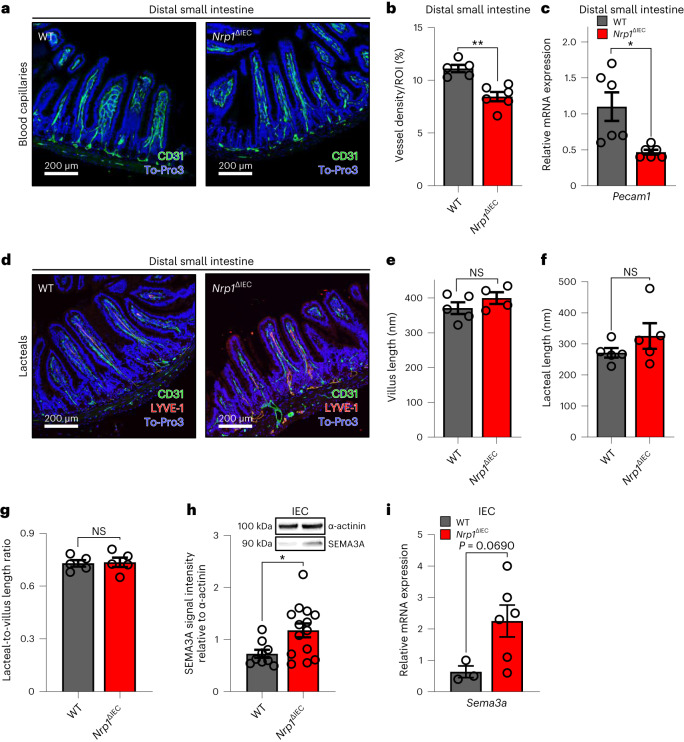
Deficiency of epithelial Nrp1 reduces density of blood capillaries without affecting lacteal length in the distal small intestine. **a**, Representative immunofluorescence images of PECAM-1 (CD31) expression (green) in the distal small intestine of mice. The analysis was repeated on *n* = 5 versus 6 mice. **b**, Quantification of CD31-positive area per villus structure, indicating vessel density (*n* = 5 versus 6, *P* = 0.0013). ROI, region of interest. **c**, Relative gene expression of *Pecam1* in the distal small intestine (*n* = 6 versus 6, *P* = 0.0103). **d**, Representative immunofluorescence images of CD31 (green) and LYVE-1 (red) in the distal small intestine of mice. The analysis was repeated on *n* = 5 versus 5 mice. **e**–**g**, Measurements of villus length (**e**), lacteal length (**f**) and lacteal-to-villus ratio (**g**) (*n* = 5 versus 5). **h**,**i**, Semaphorin 3A (SEMA3A) (**h**) protein (*n* = 9 versus 14, *P* = 0.0189) and mRNA levels (**i**) in isolated IECs (*n* = 3 versus 6). For all panels, *Nrp1*^ΔIEC^ mice are compared to floxed WT littermates (*Cre*-negative). *n* represents the number of biologically independent mice. For immunofluorescence images, cell nuclei were counterstained with To-Pro-3 iodide (blue). Scale bars, 200 μm. For each mouse, the mean measurements of 5–10 villi were taken into account and displayed as a single dot. For qPCR assays, *L32* was used as the housekeeping gene, while in western blots, protein expression is relative to α-actinin. Individual values are displayed as dots, while mean ± s.e.m. is shown as a column and error bar (**b**,**c**,**e–i**). Independent samples were analyzed by Student’s *t*-test, *P < 0.05, **P < 0.01. Source data

In the small intestine, the villus endothelial button-like junctions of lacteals, which enable the absorption of chylomicrons into the lymphatics, are stabilized through lymphatic endothelial NRP1, suppressing VEGF-A signaling^44^. As NRP1 promotes microvessel branching and tip cell guidance in the vascular endothelium^45^ and is upregulated in dysplastic epithelia forming a reservoir for the sequestration of angiogenic ligands^46^, we reasoned that epithelial NRP1 could be involved in the adaptive development of capillary networks in small intestinal villus structures^3^. Indeed, fluorescence staining for the pan-endothelial cell marker platelet endothelial cell adhesion moleculer-1 (PECAM-1, CD31) revealed a significant reduction in the density of blood capillaries in small intestinal villus structures of *Nrp1*^ΔIEC^ mice relative to *Cre*-negative WT littermate controls (Fig. 4a,b). This finding was further confirmed by the observation of reduced *Pecam1* mRNA expression in the small intestine of *Nrp1*^ΔIEC^ mice (Fig. 4c). In contrast, epithelial *Nrp1* deficiency did not compromise villus length, lacteal length or the lacteal per villus length ratio, as assessed by staining for the lymphatic marker lymphatic vessel endothelial hyaluronic acid receptor-1 (LYVE-1) (Fig. 4d–g).

**Extended Data Fig. 4:**
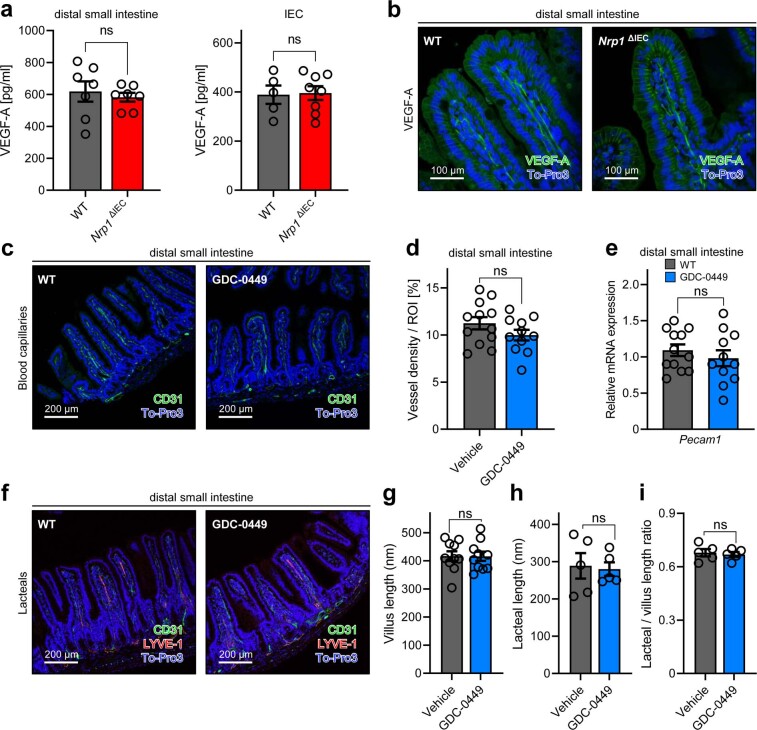
VEGF-A expression in the small intestine of the *Nrp1*^ΔIEC^ mouse line and characterization of blood capillaries and lacteals in the distal small intestine of GDC-0449-treated mice. **(a)** VEGF-A ELISA in the distal small intestine (*n* = 7 versus 7) and IECs (*n* = 5 versus 8) isolated from WT littermates versus *Nrp1*^ΔIEC^ mice. **(b)** Representative immunofluorescence images of VEGF-A expression (in green) in the distal small intestine of *Nrp1*^ΔIEC^ mice versus WT littermates. The stainings were repeated on *n* = 4 mice. For panels **c-i**, GDC-0449-treated mice are compared to vehicle controls**. (c)** Representative immunofluorescence images of PECAM-1 (CD31) expression (in green) in the distal small intestine of mice (*n* = 12 versus 11). **(d)** Quantification of CD31-positive area per villus structure (ROI: region of interest), representing vessel density (*n* = 12 versus 11). **(e)** Relative mRNA expression of *Pecam1* in the distal small intestine (*n* = 12 versus 11). *L32* was used as the housekeeping gene. **(f)** Representative immunofluorescence images of CD31 (green) and LYVE-1 (red) in the distal small intestine of mice (*n* = 10 versus 10). Measurements of **(g)** villus length (*n* = 10 versus 10), **(h)** lacteal length (*n* = 5 versus 5) and **(i)** the lacteal-to-villus ratio (*n* = 5 versus 5). For panels **a**, **d-e**, **g-i**, n represents the number of biological independent mice and individual values are shown as dots. Mean ± s.e.m. is shown as a column and error bar. For immunofluorescence images, cell nuclei are counterstained with To-Pro-3 iodide (in blue). Scale bar: 100 μm in panel **b** and 200 μm in panels **c**, **f**. For each mouse, the mean measurements of 5–10 villi were taken into account and displayed as a single dot. Unpaired Student’s *t* test, ns: *P* > 0.05. Source data

To explain this vascular phenotype, we analyzed the gut epithelial levels of the NRP1 ligand VEGF-A, assisting VEGFR-2-mediated angiogenesis and SEMA3A, an established NRP1 ligand that limits angiogenesis.^31,32,45,46,47^ Unexpectedly, VEGF-A was unaffected by intestinal epithelial *Nrp1* deficiency, as shown by ELISA of small intestinal tissue lysates and isolated epithelium, as well as by immunofluorescence staining (Extended Data Fig. 4a,b). In contrast, SEMA3A, an inhibitor of epithelial cell migration and an established vascular morphogen^45,46^, was elevated in IECs of *Nrp1*^ΔIEC^ mice, both at the mRNA and protein level (Fig. 4h,i). Hence, reduced villus vascularization in *Nrp1*^ΔIEC^ mice likely results from anti-angiogenic SEMA3A-NRP1 signaling^45,47^. In contrast to the identified role of Hh signaling in epithelial gut permeability regulation (Fig. 3m–p), in vivo inhibition of the Hh pathway with GDC-0449 did not affect villus vascularization nor lacteal length (Extended Data Fig. 4c–i). Notably, our results uncovered NRP1, expressed by small IECs, as an important regulator of gut mucosal vascularization.

## Discussion

**Fig. 5:**
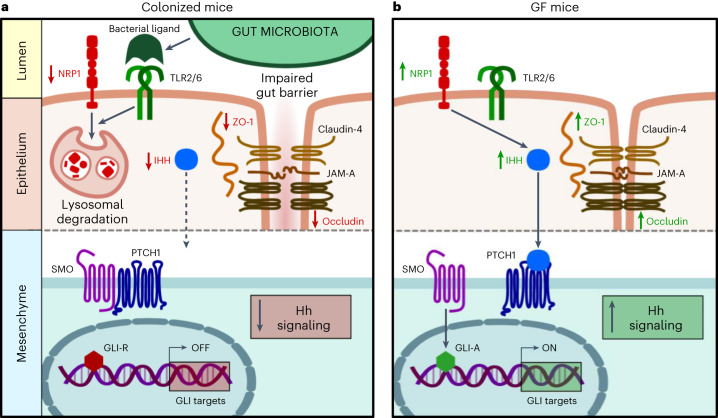
Impact of commensals on Hedgehog signaling, NRP1 and intestinal epithelial permeability. Conserved molecular patterns such as bacterial ligands from the resident gut microbiota stimulate TLRs on the IECs. In turn, TLR2/TLR6 downregulates NRP1 protein levels in the epithelial compartment through lysosomal degradation. When NRP1 is not degraded, it upregulates IHH, which signals from the epithelium to the mesenchymal compartment. **a**, In the absence of IHH downstream signaling (shown as exemplification in colonized mice), the transmembrane receptor PTCH1 suppresses the transmembrane protein SMO, resulting in a repressor form of the transcription factor GLI (GLI-R). **b**, Conversely, after IHH binding to PTCH1 (exemplified in GF mice), repression on SMO is released, yielding the GLI activator (GLI-A) transcription factor and subsequent transcription of GLI targets involved in Hh signaling (GLI targets ‘on’). In GF conditions, this pathway results in upregulation of occludin and ZO-1 protein levels, whereas in CONV-R housing conditions (**a**), the epithelial gut barrier is impaired. In the scheme, upregulated (↑) proteins are shown in green and downregulated (↓) proteins are in red.

Here, we reveal that the gut microbiota suppresses tonic Hh signaling in the small intestine, thus regulating intestinal barrier function (Fig. 5). We unveil that Hh pathway activity is primarily suppressed through microbiota-triggered TLR2/TLR6 signals in the gut epithelium and identified intestinal epithelial NRP1 as a pivotal microbiota-dependent Hh regulator, which contributes to stabilize the gut epithelial barrier. In essence, our results uncover a microbiota-driven morphogenetic signaling pathway that links intestinal innate immune receptor signaling to Hh pathway activity. This microbiota–host interaction tunes the epithelial gut barrier and thus might have broad consequences on small intestinal nutrient uptake and intestinal immune homeostasis.

While it is evident that colonization with gut commensals evokes adaptive changes in gut morphology^2–6^, it remains unclear how microbiota-derived signals become integrated into morphogenetic signaling cues at the epithelial lining of the small intestine. Our results define small intestinal epithelial TLR2 as a target of the gut microbiota that downregulates epithelial-derived *Ihh* gradients and downstream signaling in the lamina propria^21–25^. Dependent on TLR2 activation of lysosomal degradation, NRP1 on the gut epithelium is efficiently suppressed by commensals to cease the positive-feedback circuit within the Hh pathway^21,23,25,28^. Based on NIH-2T3 mouse fibroblast cell culture models stimulated with exogenous Hh ligands, recent work has demonstrated a cell-intrinsic role for NRP1, acting as a positive-feedback regulator of the Hh pathway in mesenchymal cells^28,29^. This cell-intrinsic signaling function of NRP1 critically depends on a 12-amino-acid region within the cytoplasmic domain of the receptor (amino acids 890–902) and the cytoplasmic guanosine triphosphatase-activating protein domain of multiple plexins promoting cell-intrinsic Hh signaling^29,48^. However, our in vivo analyses indicate that NRP1 expressed by the gut epithelial lining regulates microbiota-dependent expression of *Ihh*, the major small intestinal agonist situated upstream in the intestinal epithelial-to-mesenchymal Hh signaling axis, which is exclusively expressed by the gut epithelium but not the mesenchyme^23,24^. Epithelial Hh acts on its targets, such as PTCH1 and SMO in the mesenchyme. As *Bmp4* is absent in the gut epithelium but expressed at high levels in the intravillus mesenchyme^39,40,49,50^, our results confirm the epithelial-to-mesenchymal axis of the identified signaling cue by microbiota-TLR2-instructed suppression of the Hh target gene *Bmp4* (refs. ^24,37,38^). Future investigations with gnotobiotic mouse models should address whether the gut microbiota utilizes additional innate immune pathways to interfere with morphogenetic signaling.

Notably, the uncovered microbiota-induced suppression of the epithelial NRP1–Hh signaling axis^28,29^ has broad implications for the microbiota’s impact on gut barrier function^7^. Increased paracellular permeability, mRNA downregulation of tight junction constituents and a consistent reduction of epithelial ZO-1 were observed in colonized mice, in the absence of epithelial NRP1 and after the inhibition of the Hh pathway. Thus, based on experimentation with GF mice, tissue-specific knockout mouse models and inhibitor treatments, our work established a mechanistic link between host colonization status and epithelial gut barrier integrity. Furthermore, our analyses revealed that epithelial deficiency of NRP1 impairs villus blood capillary formation in the distal small intestine. However, this vascular phenotype was independent of the Hh pathway. In contrast to the established functional role of NRP1 in the vascular endothelium, the epithelial deficiency of this co-receptor did not affect the development of lacteals^44,50^. Future studies using primary organoid cultures are needed to address the barrier-regulatory molecular mechanisms influenced by distinct commensals, affecting the architecture of villus capillaries and their role in nutrient uptake. Moreover, it will be interesting to investigate how the identified epithelial microbiota-triggered TLR2–Hh signaling axis, through Hh-induced factors produced in the villus mesenchyme, affects gut epithelial barrier function. In conclusion, our work unravels the functional relevance of epithelial NRP1 as a microbiota-dependent regulatory factor of epithelial Hh signaling in the regulation of the gut epithelial barrier and mucosal vascular remodeling.

## Methods

### Animals

GF C57BL/6J Swiss Webster mice and the CONV-R controls originated from the colonies of F. Bäckhed (Wallenberg Laboratory). GF and CONV-R distal small intestinal tissues from *Tlr5*^−/−^ mice^51^ were obtained through collaboration with B. Chassaing and A. Gewirtz (Georgia State University). *Tlr2*^−/−^ mice (B6;129-Tlr2<tm1Kir>/J-M; stock 004650)^52^ were purchased from The Jackson Laboratory and *Tlr4*^−/−^ mice^53^ were provided by M. Radsak (Department of Medicine III, University Medical Center Mainz). *Tlr2*^−/−^ and *Tlr4*^−/−^ mouse strains were re-derived as GF by aseptic hysterectomy and maintained in sterile conditions as a GF mouse colony in flexible film isolator systems. The GF status of mice was tested weekly by PCR for detection of bacterial 16S rDNA and by bacterial culture of feces. *Nrp1*-flox mouse line (B6.129(SJL)-Nrp1 <tm2Ddg>/J; stock 005247)^54^ and the Villin-Cre mouse line were purchased from The Jackson Laboratory. The epithelial knockout mouse models Tlr2-flox x VilCre (*Tlr2*^ΔIEC^) and Nrp1-flox x VilCre (*Nrp1*^ΔIEC^) were generated by crossing with the B6.Cg-Tg(Vil1-cre)1000Gum/J line (stock 021504)^55^. All animals were 8–14-week-old male or female mice housed in the Translational Animal Research Center of the University Medical Center Mainz under specific-pathogen-free (CONV-R) conditions in EU type II cages with two to five cage companions with standard autoclaved laboratory diet and water ad libitum, 22 °C ± 2 °C room temperature and a 12-h light–dark cycle, 55–65% humidity. All groups of mice were age- and sex-matched and free of clinical symptoms.

For conventionalization experiments (CONV-D mice), the cecum content of two age-matched CONV-R animals (one male and one female) was re-suspended in 10 ml PBS and a 200 µl-aliquot was given to GF mice by oral gavage. The conventionalization was allowed to proceed for 2 weeks. For Abx treatment (CONV-R + Abx), an antibiotic cocktail (1.5 g l^−1^ ampicillin; 0.5 g l^−1^ neomycin) was added to the drinking water for 2 weeks. Mice were killed by CO_2_ inhalation, followed by cervical dislocation. All procedures performed on mice were approved by the local committee on legislation on protection of animals (Landesuntersuchungsamt Rheinland-Pfalz; G12-1-035; G17-1-075; G20-1-119; A18-1-005).

### GDC-0449 treatment

A total of 50 mg GDC-0449 (cat. no. S1082, Selleck Chemicals) were dissolved in 8.3 ml 0.5% (w/v) methylcellulose (cat. no. 8421.1, Carl Roth), 0.2% (v/v) Tween-80 (cat. no. 9139.1, Carl Roth) and 0.5% (v/v) dimethylsulfoxide (DMSO; cat. no. 276855, Sigma-Aldrich), thus obtaining a 6 mg ml^−1^ stock solution. Each day, for 7 d, the mice were given an aliquot of the GDC-0449 suspension or the vehicle by oral gavage (30 mg kg^−1^ body weight). At 4 h before the gavage, the drinking water was removed from the cage. On the last day, 4 h after the gavage, mice were killed and organs were extracted.

### Organ collection

After death, the abdominal cavity was excised and the small intestine was extracted, cleaned from adipose tissue and flushed from mucous and fecal content with ice-cold PBS (cat. no. BP399, Fisher BioReagents). For whole-tissue analysis, a segment of the distal small intestine was snap-frozen in liquid nitrogen or, in the case of histological analysis, was fixated in ROTIHistofix 4.5% (cat. no. 2213.3, Carl Roth) for 24 h before paraffin embedding. For preparation of frozen sections this proceeded as described elsewhere^3^.

Primary IECs were isolated as detailed elsewhere^41^. Briefly, the PBS-cleaned distal small intestine was cut open lengthwise and incubated with 5 ml 10 mM EDTA (cat no. A4892,0500, AppliChem) in PBS under agitation (250 r.p.m. for 30 min at 37 °C). After manual shaking (removal of the epithelial layer), IECs were collected (6,500 r.p.m. for 5 min at 4 °C), washed in PBS and re-suspended in the proper buffer according to total RNA or protein extraction.

### MODE-K cell culture

MODE-K cells were purchased from Inserm-U1111 (Dr Kaiserlian)^36^. In brief, cells were maintained at 37 °C in a humidified atmosphere of 5% CO_2_ with RPMI 1640 Medium, GlutaMAX (cat no. 11554516, Gibco, Thermo Fisher Scientific) supplemented with 100 mM sodium pyruvate (cat. no. 11360070), 1 M HEPES (cat. no. 15630080), 1% (v/v) non-essential amino acids (cat. no. 11140050), 50 mM 2-mercaptoethanol (cat. no. 21985023), 10% (v/v) heat-inactivated fetal bovine serum (cat. no. 10082147) (all from Thermo Fisher Scientific) and 0.2% (v/v) penicillin–streptomycin (cat. no. P4333, Sigma-Aldrich). For stimulation, MODE-K cells were seeded in six-well plates until they reached 80–90% confluence, followed by treatment in cell culture medium for 2 h with 0.125% (v/v) bafilomycin A1 (cat. no. SML1661, Sigma-Aldrich), 0.1% (v/v) epoxomicin (cat. no. 324801, Sigma-Aldrich), 0.5 µM Pam_3_CSK_4_ (cat. no. 506350, Sigma-Aldrich) or 2 µg ml^−1^ MALP-2 (cat. no. ALX-162-027-C050, Enzo Life Sciences). For the vehicle, the equivalent amount of DMSO (0.1–0.125% (v/v)) (cat. no. A3672,0050, AppliChem) was used. MODE-K cells were manually detached with a cell scraper for western blot, followed by protein extraction. For flow cytometry analysis, cells were re-suspended.

### Flow cytometry of MODE-K cells

MODE-K cells were collected and re-suspended in ice-cold 1× PBS added with 3% (v/v) fetal bovine serum (cat. no. 10082147, Thermo Fisher Scientific), then, FACS buffer. Next, MODE-K cells were pre-incubated with 1:100 (v/v) rat anti-mouse CD16/32 TruStain FcX monoclonal antibody (clone 93, cat. no. 101319, BioLegend) for 10 min on ice. After pelleting (300*g*, 10 min, 4 °C) and washing in FACS buffer, MODE-K cells were incubated with fluorescent antibodies for 30 min at 4 °C, protected from light. The following antibodies and dilutions were used: 1:20 (v/v) EPCAM (CD326)-PerCP-eFluor 710 (clone G8.8, cat. no. 46-5791, Thermo Fisher Scientific); 1:100 (v/v) NRP1 (CD304)-PE (clone 3E12, cat. no. 145203) and 1:100 (v/v) IgG2a, κ isotype control (clone RTK2758, cat. no. 400501), both from BioLegend. After washing with ice-cold PBS (300*g*, 10 min, 4 °C), cells were re-suspended in 500 µl PBS and analyzed by flow cytometry on a BD FACSCanto II instrument (BD Biosciences). Data were visualized on BD FACSDiva Software (v.6.1.3) and analyzed by FlowJo (v.10.5.2). The gating strategy was based on the isotype control.

### Immunofluorescence

Paraffin embedding of small intestinal sections was performed by the Histology Core Facility (University Medical Center Mainz). After slicing the embedded tissue in 7-μm sections using a rotatory microtome (Leica Biosystems), sections were deparaffinized in xylol (cat. no. 251769, AppliChem) and rehydrated in a graded series of ethanol and water. After antigen retrieval by steaming in 10 mM citric acid (cat. no. 5110, Carl Roth) for 20 min and 2 × 5 min washing in PBS-T (PBS + 0.05 % (v/v) Tween-20), samples were blocked with protein block solution (cat. no. X0909, Agilent) for 10 min. The following primary antibodies were used overnight at 4 °C: 1:100 (v/v) PECAM-1 rabbit monoclonal antibody (CD31, clone D8V9E, cat. no. 77699, Cell Signaling Technology), 1:100 (v/v) LYVE-1 rat monoclonal antibody (clone ALY7, cat. no. 14-0443-80, Thermo Fisher Scientific) and 1:100 VEGF-A rabbit monoclonal antibody (cat. no. ab52917, Abcam). After 3 × 5 min-washes in PBS-T, secondary antibodies were incubated for 1 h and protected from light. In particular, 1:500 (v/v) goat anti-rabbit IgG (H + L) conjugated with Alexa Fluor 488 (cat. no. 4412, Cell Signaling Technologies) was used to detect PECAM-1 and VEGF-1, whereas 1:500 (v/v) goat anti-rat IgG (H + L) conjugated with Alexa Fluor 555 (cat. no. 4417, Cell Signaling Technologies) was used against LYVE-1. Samples were washed for 3 × 5 min in PBS-T, counterstained with To-Pro3 iodide (cat. no. T3605, Thermo Fisher Scientific) for 30 min and subsequently, mounted with Faramount (cat. no. S3025, Agilent). For fluorescent staining on frozen sections from the distal small intestine of Swiss Webster mice, 6-μm cryosections were cut and stored at −80 °C. On the day of the experiment, samples were thawed at room temperature for 20 min and samples were blocked for 1 h in TBST (TBS + 0.1 % (v/v) Tween-20), added with 10% (v/v) fecal calf serum (heat-inactivated, cat. no. AL2420, Life Technologies). NRP1 rabbit monoclonal antibody (clone D62C6, cat. no. 3725, Cell Signaling Technology) was diluted 1:2,000 (v/v) in blocking buffer and added on the slides for 2.5 h at room temperature. Slides were washed for 3 × 10 min in TBST and incubated with 1:1,000 (v/v) anti-rabbit secondary antibodies (goat anti-rabbit IgG (H + L) conjugated with Alexa Fluor 488 (cat. no. 4412, Cell Signaling Technologies) or donkey anti-rabbit IgG (H + L) Alexa Fluor 555 (cat. no. A-31572, Life Technologies)) for 1 h in the dark. Slides were washed for 3 × 10 min in TBST and nuclei were counterstained with DAPI (cat. no. 32670, Sigma-Aldrich). Slides were washed again for 3 × 10 min in TBST and mounted with Faramount reagent. Images were acquired using a Zeiss LSM 710 microscope, following morphometric analysis employing CellSens Dimension (v.4.1) (Carl Zeiss).

### qRT–PCR analyses

Total RNA was isolated from whole tissue with TRIreagent (cat. no. T9424, Sigma-Aldrich) as detailed elsewhere^56^ and from IECs with the Promega kit (cat. no. Z6012, Promega), according to the manufacturer’s instructions. After purity and quality checks, mRNA was converted into complementary DNA with a High-Capacity cDNA Reverse Transcriptase kit (cat. no. 4368814, Applied Biosystems). The cDNA was diluted 1:20 (v/v) by RNase-free water (Aqua ad iniectabilia, B. Braun). Relative mRNA expression was quantified by qPCR analyses on a qTOWER3 Real-Time PCR Thermal Cycler instrument (Analytic Jena) equipped with qPCRsoft (v.4.0) software, using iTaq Universal SYBR Green Supermix (cat. no. 1725121, Bio-Rad Laboratories). Each biological sample was analyzed in triplicate, using the ribosomal protein *L32* as the housekeeping gene. For relative expression quantification, cycle threshold (Ct) values were analyzed, according to Pflaffl^57^ and normalized for the control group: GF mice, when different microbiota colonization statuses were compared, WT mice, in case of the analyses on transgenic mouse strains, and control mice, in case of GDC-0449 treatment. For each gene of interest, the sequences of the forward and reverse primers used are listed in Supplementary Table 1.

### Single-molecule fluorescence in situ hybridization

RNAscope Multiplex Fluorescent V2 assay (cat. no. PS-00003027.1, Bio-Techne) was performed on 5-μm paraffin sections (for paraffin embedding and cutting with microtome see above) according to the manufacturer’s instruction. *Gli1*, *Ptch1* and *Hhip* transcripts were ordered from the company and probed using TSA Vivid Fluorophore 650, 520 and 570, respectively. Cell nuclei were counterstained with DAPI. Distal small intestine of GF versus CONV-R and *Tlr2*^ΔIEC^ versus WT-floxed littermates were analyzed, using three mice per group. The probes were visualized with a confocal microscope (Leica TCS SP8). Data analysis was accomplished with the Leica Application Suite X (LAS EZ) software (v.3.7.5.24914).

### qPCR array

The RT2 profiler PCR array kit (cat. no. PAMM-078Z, QIAGEN) was used according to the manufacturer’s instructions. RNA was isolated from the distal small intestine of seven mice per group and adjusted to a concentration of 200 ng µl^−1^. cDNA was synthetized using the RT^2^ First Strand kit (cat. no. 330404, QIAGEN). Per group, one 96-well plate was used. The obtained data were analyzed using the online software RT2 Profiler PCR Array Data Analysis (v.3.5)^58^.

### Western blot

Small intestine tissues, isolated IECs or MODE-K cells were re-suspended in a variable volume of RIPA Buffer (cat. no. 20-188, Merck Millipore) added with protease and phosphatase inhibitor mini-tablets (cat. no. A32959, Thermo Fisher Scientific). For small intestinal specimens, mechanical lysis was performed on a TissueLyserII (QIAGEN) (2 × 2 min, 30 Hz, with a 1-min pause in between). Protein extraction was allowed to proceed for 30 min on ice. Samples were centrifuged (10,000*g*, 15 min, 4 °C) and protein quantification was performed on collected supernatants by DC Protein Assay (cat. no. 500-0116, Bio-Rad Laboratories). All samples were diluted to the same concentration and added to 5× home-made reducing Laemmli buffer. Thermic denaturation was promoted at 99 °C for 5 min. For western blot assays, proteins were separated on an electrophoretic run and transferred on a 0.45-µm PVDF membrane (cat. no. IPVH00010, Merck Millipore). The membranes were blocked in 5% (w/v) milk or BSA in TBST (TBS + 0.1% (v/v) Tween-20) and incubated overnight with the primary antibodies at 4 °C. The following antibodies were used: 1:1,000 (v/v) IHH (cat. no. ARP45230_T100, Aviva System Biology), 1:1,000 (v/v) NRP1 (cat. no. 3725S, Cell Signaling Technology), 1:1,000 (v/v) NRP2 (cat. no. 3366P, Cell Signaling Technology), 1:1,000 (v/v) occludin (cat. no. OC-3F10, Thermo Fisher Scientific), 1:1,000 (v/v) ZO-1 (cat. no. 61-7300, Thermo Fisher Scientific) and 1:1,000 (v/v) SEMA3A (cat. no. ab23393, Abcam). The 1:2,500 (v/v) α-actinin (cat. no. 3134S, Cell Signaling Technology) and 1:1,000 (v/v) β-actin (cat. no. 4970S, Cell Signaling Technology) were used as loading controls. After 3 × 10-min-washes in TBST, secondary 1:5,000 (v/v) goat anti-rabbit IgG (H + L) (cat. no. PI-1000) or 1:2,500 (v/v) horse anti-mouse IgG (H + L) (cat. no. PI-2000, both from Vector Laboratories), conjugated with HRP, were added for 90 min at room temperature. Relative protein expression was quantified by ECL (cat. no. 95538S, Cell Signaling Technology) and visualized on the FusionCapt Advance (Vilber Lourmat). Band densitometry was performed on the instrument software (v.17.01) and normalized for the control group.

### VEGF-A ELISA

Small intestine tissues or IECs were processed as described before to obtain protein lysates. VEGF-A ELISA was performed according to manufacturer instructions using the VEGF-A-Cell Lysate Mouse ELISA kit (cat. no. EMVEGFACL, Invitrogen, Thermo Fisher Scientific). Raw absorbance values were read on a Dynex Opsys MR Reader (Dynex Technologies), equipped with the Revelation Quicklink software (v.4.25). The standard curve was constructed by plotting the mean absorbance of each standard on the *y* axis against the concentration on the *x* axis and then used for the calculation of the VEGF-A concentration in the samples.

### FITC-dextran gavage

Water bottles were removed from the cages 4 h before administration of 50 mg FITC-dextran (MW 4000) (cat. no. 46944, Sigma-Aldrich) per 100 g body weight in PBS. After 4 h, the mice were anesthetized by intraperitoneal injection of a solution of 5.0 mg kg^−1^ body weight midazolam (Ratiopharm), 0.5 mg kg^−1^ body weight medetomidine (Pfizer) and 0.05 mg kg^−1^ body weight fentanyl (CuraMed Pharma) in 0.9% NaCl solution. Therefore, whole blood without anticoagulants was collected by cardiac puncture^59^ and stood overnight at 4 °C to coagulate. The next morning, serum was obtained by centrifugation (6,500 r.p.m., 10 min, 4 °C) of whole blood, aliquoted and stored at −20 °C for further analyses. The serum samples were diluted 1:1 (v/v) with PBS and measured in duplicate by spectrofluorometry (Fluoroskan Ascent FL, Thermo Fisher or SpectraMax MiniMax 300 Imaging CYtometer, Molecular Devices) using an excitation of 485 nm and an emission wavelength of 528 nm. A standard serially diluted FITC-dextran (0, 125, 250, 500, 1,000, 2,000, 4,000, 6,000 and 8,000 ng ml^−1^) was used for quantification. Fluorescence values were normalized to the control group.

### 16S rRNA gene sequencing and processing

Briefly, the hypervariable regions V1–V2 of the 16S rRNA genes were amplified following a dual-indexing approach sequencing on the Miseq Illumina platform^60^. Final sample sizes included 24 intestine tissues (18 *Nrp1*^ΔIEC^ and 6 WT) and 23 intestinal content samples (18 *Nrp1*^ΔIEC^ and 5 WT). Initial sequence processing was performed in Mothur (v.131.2)^61^ where forward and reverse reads were merged using a quality score parameter insert of 30 and screened for accurate length, base ambiguity and homopolymers. Chimera were detected and removed in Uchime with a reference-based method^62^. Subsequently, sequences were classified from the phylum to genus level using stasta^63^ and final reads were normalized to 4,100 reads per sample. Finally, operational taxonomic units at a 97% sequence similarity threshold were clustered in Mothur.

### Statistical analysis

GraphPad Prism v.9.3.1 was used for all statistical analyses and graphs except for the 16S rRNA gene-sequencing data. Statistical analyses of the metagenomics data were performed in R^64^. We assessed variations in microbiota structure and diversity across genotypes using several diversity measures, within tissues and content samples, distinctively. First, we evaluated variation in abundance of the most abundant (major) phyla and genera across genotypes using linear mixed-effects models with genotype, sex and sex:genotype included as fixed effects and breeding cage as a random effect in the ‘nlme’ R package. Best models were fitted by maximizing the restricted log-likelihood and evaluated and validated by (1) checking residuals distribution; (2) plotting fitted and residual values; and (3) inspecting residuals and explanatory variables. Additionally, we calculated different diversity measures including Shannon and Bray–Curtis, based on operational taxonomic units at a 97% sequence similarity threshold in the ‘vegan’ R package^65,66^. Comparison of Shannon measures across genotypes was assessed through linear mixed-effects models as explained above. The Bray–Curtis measure was assessed across genotypes using the ‘adonis’ function with 10^5^ permutations and principal coordinate analyses.

### Reporting summary

Further information on research design is available in the Nature Portfolio Reporting Summary linked to this article.

## Supplementary information

Reporting Summary

Supplementary Table 1 qPCR primer nucleotide sequences.

## Data Availability

The 16S rRNA gene-sequencing data are accessible in the Sequence Read Archive under accession no. PRJNA936417. Source data are provided with this paper.

## Source data

Source Data Fig. 1 Statistical source data for Fig. 1.
Source Data Fig. 1 Unprocessed western blots for Fig. 1.
Source Data Fig. 1 Micrographs for Fig. 1.
Source Data Fig. 2 Statistical source data for Fig. 2.
Source Data Fig. 2 Unprocessed western blots for Fig. 2.
Source Data Fig. 2 Micrographs for Fig. 2.
Source Data Fig. 3 Statistical source data for Fig. 3.
Source Data Fig. 3 Unprocessed western blots for Fig. 3.
Source Data Fig. 4 Statistical source data for Fig. 4.
Source Data Fig. 4 Unprocessed western blots for Fig. 4.
Source Data Fig. 4 Micrographs for Fig. 4.
Source Data Extended Data Fig. 1 Statistical source data for Extended Data Fig. 1.
Source Data Extended Data Fig. 2 Statistical source data for Extended Data Fig. 2.
Source Data Extended Data Fig. 2 Unprocessed western blots for Extended Data Fig. 2.
Source Data Extended Data Fig. 2 Micrographs for Extended Data Fig. 2.
Source Data Extended Data Fig. 3 Statistical source data for Extended Data Fig. 3.
Source Data Extended Data Fig. 3 Unprocessed western blots for Extended Data Fig. 3.
Source Data Extended Data Fig. 4 Statistical source data for Extended Data Fig. 4.
Source Data Extended Data Fig. 4 Micrographs for Extended Data Fig. 4.

## Code availability

The analyses investigating 16S rRNA sequences were carried out following the vignettes for the R package v.2.5-6 or the R package v.3.1-150, with built-in functions in the described R packages. All user-set parameters are reported in Methods.
