# Author Correction: Commensal bacteria weaken the intestinal barrier by suppressing epithelial neuropilin-1 and Hedgehog signaling

**DOI:** 10.1038/s42255-023-00901-z

**Published:** 2023-09-11

**Authors:** Giulia Pontarollo, Bettina Kollar, Amrit Mann, My Phung Khuu, Klytaimnistra Kiouptsi, Franziska Bayer, Inês Brandão, Valeriya V. Zinina, Jennifer Hahlbrock, Frano Malinarich, Maximilian Mimmler, Sudhanshu Bhushan, Federico Marini, Wolfram Ruf, Meriem Belheouane, John F. Baines, Kristina Endres, Scott M. Reba, Verena K. Raker, Carsten Deppermann, Christoph Welsch, Markus Bosmann, Natalia Soshnikova, Benoit Chassaing, Mattias Bergentall, Felix Sommer, Fredrik Bäckhed, Christoph Reinhardt

**Affiliations:** 1 Center for Thrombosis and Hemostasis (CTH), University Medical Center Mainz, Johannes Gutenberg-University Mainz, Mainz, Germany; 2 Department of Chemistry, Biochemistry, Johannes Gutenberg-University Mainz, Mainz, Germany; 3 German Center for Cardiovascular Research (DZHK), Partner Site RhineMain, Mainz, Germany; 4 Institute of Molecular Medicine, University Medical Center Mainz, Johannes Gutenberg-University Mainz, Mainz, Germany; 5 Institute of Anatomy and Cell Biology, Unit of Reproductive Biology, Justus-Liebig-University of Giessen, Giessen, Germany; 6 Institute of Medical Biostatistics, Epidemiology and Informatics (IMBEI), University Medical Center Mainz, Johannes Gutenberg-University Mainz, Mainz, Germany; 7 Institute for Experimental Medicine, Kiel University and Max Planck Institute for Evolutionary Biology, Plön, Germany; 8 Department of Psychiatry and Psychotherapy, University Medical Center Mainz, Johannes Gutenberg-University Mainz, Mainz, Germany; 9 Department of Medicine, Case Western Reserve University and University Hospitals Cleveland Medical Center, Cleveland, OH USA; 10 Department of Internal Medicine 1, Goethe University Hospital Frankfurt, Frankfurt am Main, Germany; 11 Pulmonary Center, Department of Medicine, Boston University School of Medicine, Boston, MA USA; 12 INSERM U1016, Team ‘Mucosal microbiota in chronic inflammatory diseases’, CNRS UMR 8104, Université de Paris, Paris, France; 13 Department of Molecular and Clinical Medicine, Wallenberg Laboratory, Institute of Medicine, University of Gothenburg, Gothenburg, Sweden; 14 Institute of Clinical Molecular Biology, Christian-Albrechts-University, Kiel, Germany; 15 Novo Nordisk Foundation Center for Basic Metabolic Research, Section for Metabolic Receptology and Enteroendocrinology, Faculty of Health Sciences, University of Copenhagen, Copenhagen, Denmark; 16 Department of Clinical Physiology, Region Västra Götland, Sahlgrenska University Hospital, Gothenburg, Sweden

**Keywords:** Toll-like receptors, Microbiome, Metabolism, Gastrointestinal system

Correction to: *Nature Metabolism* 10.1038/s42255-023-00828-5. Published online 6 July 2023.

In the version of this article originally published, authors Wolfram Ruf and Christoph Reinhardt (both affiliated with the Center for Thrombosis and Hemostasis (CTH), University Medical Center Mainz, Johannes Gutenberg-University Mainz, Mainz, Germany and the German Center for Cardiovascular Research (DZHK), Partner Site RhineMain, Mainz, Germany) were mistakenly shown as being affiliated with the Department of Chemistry, Biochemistry, Johannes Gutenberg-University Mainz instead of the DZHK. The error has been corrected in the HTML and PDF versions of the article.

