# Supplementary material for: Commensal bacteria weaken the intestinal barrier by suppressing epithelial neuropilin-1 and Hedgehog signaling
Source: Nat Metab. 2023 Jul 6;5(7):1174–87. doi: 10.1038/s42255-023-00828-5 (PMC10365997; doi:10.1038/s42255-023-00828-5)
Supplement: Supplementary file 1 — Reporting Summary [file 42255_2023_828_MOESM1_ESM.pdf]

nature portfolio

Corresponding author(s): Christoph Reinhardt

Last updated by author(s): Mar 31, 2023

Reporting Summary

Nature Portfolio wishes to improve the reproducibility of the work that we publish. This form provides structure for consistency and transparency in reporting. For further information on Nature Portfolio policies, see our Editorial Policies and the Editorial Policy Checklist.

Statistics

For all statistical analyses, confirm that the following items are present in the figure legend, table legend, main text, or Methods section.

n/a	Confirmed	
☐	☒	The exact sample size (*n*) for each experimental group/condition, given as a discrete number and unit of measurement
☐	☒	A statement on whether measurements were taken from distinct samples or whether the same sample was measured repeatedly
☐	☒	The statistical test(s) used AND whether they are one- or two-sided<br>*Only common tests should be described solely by name; describe more complex techniques in the Methods section.*
☒	☐	A description of all covariates tested
☒	☐	A description of any assumptions or corrections, such as tests of normality and adjustment for multiple comparisons
☐	☒	A full description of the statistical parameters including central tendency (e.g. means) or other basic estimates (e.g. regression coefficient) AND variation (e.g. standard deviation) or associated estimates of uncertainty (e.g. confidence intervals)
☐	☒	For null hypothesis testing, the test statistic (e.g. *F*, *t*, *r*) with confidence intervals, effect sizes, degrees of freedom and *P* value noted<br>*Give P values as exact values whenever suitable.*
☒	☐	For Bayesian analysis, information on the choice of priors and Markov chain Monte Carlo settings
☒	☐	For hierarchical and complex designs, identification of the appropriate level for tests and full reporting of outcomes
☒	☐	Estimates of effect sizes (e.g. Cohen's *d*, Pearson's *r*), indicating how they were calculated

Our web collection on statistics for biologists contains articles on many of the points above.

Software and code

Policy information about availability of computer code

Data collection	- qPCR: qTOWER³ Real-Time PCR Thermal Cycler<br>- Western Blot: FusionCapt Advance (Vilber Lourmat)<br>- Flow cytometry data visualization: BD FACSDiva Software (version 6.1.3)<br>- Immunofluorescence: Zeiss LSM 710 microscope<br>- sm-FISH: Leica TCS SP8 Wetzlar confocal microscope<br>- 16S rRNA amplification: Miseq Illumina platform<br>- 16S rRNA sequencing: Mothur (version 1 31.2)<br>- FITC-Dextran gavage: Fluoroskan Ascent FL (Thermo Fisher) or SpectraMax MiniMax 300 Imaging CYtometer (Molecular Devices)<br>- ELISA: Dynex Opsys MR Reader (Dynex Technologies)

Data analysis	For all the graphs and statistical analyses: GraphPad 9.3.1<br>Exclusion of outliers: GraphPad outlier calculator - online software<br><br>- qPCR: qTOWER³ Real-Time PCR Thermal Cycler - instrument software qPCRsoft (version 4.0)<br>- qPCR array: RT2 Profiler PCR Array Data Analysis (version 3.5) - online software<br>- Western Blot: FusionCapt Advance (Vilber Lourmat) - instrument software (version 17.01)<br>- Flow cytometry: FlowJo Software (version 10.5.2)<br>- Immunofluorescence: CellSens Dimension - instrument software (version 4.1)<br>- sm-FISH: Leica Application Suite X (LAS EZ) software (version 3.7.5.24914)<br>- 16S rRNA sequencing: statistical analyses performed in R<br>- ELISA: Revelation Quicklink - instrument software (version 4.25).

For manuscripts utilizing custom algorithms or software that are central to the research but not yet described in published literature, software must be made available to editors and reviewers. We strongly encourage code deposition in a community repository (e.g. GitHub). See the Nature Portfolio guidelines for submitting code & software for further information.

Data

Policy information about availability of data

All manuscripts must include a data availability statement. This statement should provide the following information, where applicable:

- Accession codes, unique identifiers, or web links for publicly available datasets
- A description of any restrictions on data availability
- For clinical datasets or third party data, please ensure that the statement adheres to our policy

All data generated or analysed during this study are included in this published article (and its supplementary information files). The datasets generated and/or analysed during the current study are available from the corresponding author on reasonable request. 16S rRNA gene sequencing data are accessible in Sequence Read Archive (SRA) under PRJNA936417.

Human research participants

Policy information about studies involving human research participants and Sex and Gender in Research.

Reporting on sex and gender	N/A
Population characteristics	N/A
Recruitment	N/A
Ethics oversight	N/A

Note that full information on the approval of the study protocol must also be provided in the manuscript.

Field-specific reporting

Please select the one below that is the best fit for your research. If you are not sure, read the appropriate sections before making your selection.

☒ Life sciences ☐ Behavioural & social sciences ☐ Ecological, evolutionary & environmental sciences

For a reference copy of the document with all sections, see nature.com/documents/nr-reporting-summary-flat.pdf

Life sciences study design

All studies must disclose on these points even when the disclosure is negative.

Sample size	For each experiment, no statistical methods were used to predetermine sample size. Instead, we chose samples size (at least 4 biological replicates) based on previous experiments performed with the same method.
Data exclusions	Exclusion of data was based on the free 'outlier calculator' software by GraphPad, choosing Alpha = 0.05 as the significant level.<br>For GDC-0449 treatment, we excluded animals that, according to the expression level of Gli1 (Hh-downstream signaling target), did not respond to the treatment (i.e. calculated as outliers).
Replication	qPCR: for each biological replicate (=1 mouse), 3 technical replicates (3 for gene of interest + 3 for housekeeping gene) were performed.<br>qPCR array: the expression bars represent the ratio of the mean expression of 7 biological replicates per group.<br>Western Blot, FITC-Dextran 4000 measurements, VEGF-ELISA: each biological replicate (represented by a dot) was repeated only once.<br>Immunofluorescence measurements: measurements of CD31+ area, villus length and lacteal length were performed on 5-10 villi per mouse. Each dot represents the mean of these measurements.<br>MODE-K stimulation and WB/FACS analyses: each dot represents a technical replicate.<br>Microbiota sequencing: the bars represent the mean of 23-24 mice in total.

sm-FISH: 3 biological replicates were used in each group, 1 slide per mouse

For all these experiments, all attempts at replication were successful.

Randomization	For GDC-0449 treatment the mice were randomly allocated, in order to have sex- and age-matched groups. We confirmed effectiveness of treatment by analyzing the suppression of the Hedgehog-target Gli1.<br>For the other mouse experiments, mice were randomly allocated in the groups, based on verified genotype (wildtype vs. knockout littermates) or colonization status (germ-free, conventionally-raised). For antibiotics treatments and mono-colonization with Bacteroides thetaiotaomicron, the groups of mice were randomly allocated, in order to have sex- and age-matched groups.
Blinding	Organ harvesting from knockout mice vs. WT littermates was blinded. Conversely, organ collection from treated animals vs controls was not, as the animals were allocated in different cages due to experiment set up, and to enable daily scoring of mice.<br>MODE-K stimulation, flow cytometry analyses, qPCR, Western Blot, FITC-dextran gavage and ELISA were not blinded. Here, blinding was not relevant for the analysis, since all samples were taken into account and the read outs were detected in an automated fashion.<br>On the other hand, immunofluorescence staining and evaluation was blinded.

Reporting for specific materials, systems and methods

We require information from authors about some types of materials, experimental systems and methods used in many studies. Here, indicate whether each material, system or method listed is relevant to your study. If you are not sure if a list item applies to your research, read the appropriate section before selecting a response.

Materials & experimental systems

n/a	Involved in the study
☐	☒ Antibodies
☐	☒ Eukaryotic cell lines
☒	☐ Palaeontology and archaeology
☐	☒ Animals and other organisms
☒	☐ Clinical data
☒	☐ Dual use research of concern

Methods

n/a	Involved in the study
☒	☐ ChIP-seq
☐	☒ Flow cytometry
☒	☐ MRI-based neuroimaging

Antibodies

Antibodies used

PRIMARY ANTIBODIES, NON-FLUORESCENT :
- IHH Rabbit pAb (Cat.# ARP45230_T100, Aviva System Biology)
- CD31 (PECAM-1), clone D8V9E XP Rabbit mAb (Cat #77699, Cell Signaling Technology)
- LYVE-1, clone ALY7 Rat mAb (Cat #14-0443-82, Thermo Fisher Scientific)
- VEGF-A, clone EP1176Y Rabbit mAb (Cat #ab52917, Abcam)
- Neuropilin-1 (NRP1), clone D62C6 Rabbit mAb (Cat #3725, Cell Signaling Technology)
- Neuropilin-2 (NRP2), clone D39A5 Rabbit mAb (Cat #3366, Cell Signaling Technology)
- Occludin, clone OC-3F10 mouse mAb (Cat #33-1500, Thermo Fisher Scientific)
- ZO1 Rabbit pAb (Cat #61-7300, Thermo Fisher Scientific)
- Semaphorin 3A (SEMA3A) Rabbit pAb (Cat #ab23393, Abcam)
- α-Actinin Rabbit pAb (Cat #3134S, Cell Signaling Technology)
- β-Actin, clone 13E5, Rabbit mAb (Cat #4970S, Cell Signaling Technology)
- anti-Mouse CD16/32 TruStain FcX, clone 93, Rat mAb (Cat #101319, Biolegend)

PRIMARY ANTIBODIES, FLUORESCENT:
- EpCAM (CD326)-PerCP-eFluor 710, clone G8.8 (Cat #46-5791, Thermo Fisher Scientific)
- NRP1 (CD304)-PE, clone 3E12 (Cat #145203, Biolegend)
- IgG2a, κ Isotype Control-PE, clone RTK2758 (Cat #400501, Biolegend)

SECONDARY ANTIBODIES, HRP-CONJUGATED:
- Goat anti-rabbit IgG (H+L), HRP-conjugated (Cat #PI-1000, Vector Laboratories)
- Horse anti-mouse IgG (H+L), HRP-conjugated (Cat #PI-2000, Vector Laboratories)

SECONDARY ANTIBODIES, FLUORESCENT:
- Goat anti-rabbit IgG (H+L), F(ab')2 Fragment Alexa Fluor 488 (Cat #4412, Cell Signaling Technology)
- Goat anti-rat IgG (H+L) Alexa Fluor 555 (Cat #4417, Cell Signaling Technology)
- Goat anti-rabbit IgG (H+L) conjugated with FITC (Cat #AS011, abcam)
- Donkey anti-rabbit IgG (H+L) Alexa Fluor 555 (Cat #A-31572, Life Technologies)

Validation

Aviva Abs: enhanced validation by WB, SPR and YCHAROS.
- IHH Rabbit pAb (Cat.# ARP45230_T100). Tested species reactivity: human, mouse.

Cell Signaling Technology Abs: Species reactivity is determined by testing in at least one approved application (e.g., western blot).
- CD31 (PECAM-1), clone D8V9E XP Rabbit mAb (Cat #77699). Tested species reactivity: mouse. Validated for WB, IHC Leica Bond and IHC (paraffin)
- Neuropilin-1 (NRP1), clone D62C6 Rabbit mAb (Cat #3725). Tested species reactivity: human, mouse, rat. Validated for WB and IP.

- Neuropilin-2 (NRP2), clone D39A5 Rabbit mAb (Cat #3366). Tested species reactivity: mouse, rat. Validated for WB, IP, IHC (paraffin), IF (frozen).
- α-Actinin Rabbit pAb (Cat #3134S). Tested species reactivity: human, mouse, rat, hamster, monkey. Validated for WB, IF and IHC.
- β-Actin, clone 13E5, Rabbit mAb (Cat #4970S). Tested species reactivity: human, mouse, rat, monkey, bovine, pig. Validated for WB, simple Western, IHC (paraffin), IF (frozen), IF, flow cytometry (fixed/permeabilized).

Thermo Fisher Scientific Abs: Verified by Cell treatment to ensure that the antibodies bind to the antigen stated.
- LYVE-1, clone ALY7 Rat mAb (Cat #14-0443-82). Tested species reactivity: mouse. Validated for IHC, IHC (PFA fixed) IHC (frozen), ICC/IF, flow cytometry.
- Occludin, clone OC-3F10 mouse mAb (Cat #33-1500). Tested species reactivity: dog, human, mouse, rat. Validated for WB, IHC, IHC (paraffin), IHC (frozen), ICC/IF, flow cytometry, ELISA, IP, in situ PLA.
- ZO1 Rabbit pAb (Cat #61-7300). Tested species reactivity: dog, guinea pig, human, mouse, rat. Validated for WB, IHC, IHC (paraffin, PFA fixed, frozen, free floating), ICC/IF, flow cytometry, ELISA, IP, ChIP assay.
- EpCAM (CD326)-PerCP-eFluor 710, clone G8.8 (Cat #46-5791). Tested species reactivity: mouse. Validated for flow cytometry.

Abcam Abs: as stated from the website, 'extensive validation'.
- VEGF-A, clone EP1176Y Rabbit mAb (Cat #ab52917). Tested species reactivity: mouse, human. Validated for IHC (paraffin), ICC/IF, flow cytometry.
- Semaphorin 3A (SEMA3A) Rabbit pAb (Cat #ab23393). Tested species reactivity: rat, human, recombinant fragment. Validated for IHC (paraffin), WB.

Biolegend Abs: Specificity testing of 1-3 target cell types with either single- or multi-color analysis (including positive and negative cell types). Once specificity is confirmed, each new lot must perform with similar intensity to the in-date reference lot. Brightness (MFI) is evaluated from both positive and negative populations.
Each lot product is validated by QC testing with a series of titration dilutions.
- anti-Mouse CD16/32 TruStain FcX, clone 93, Rat mAb (Cat #101319). Tested species reactivity: mouse. Validated for flow cytometry.
- NRP1 (CD304)-PE, clone 3E12 (Cat #145203). Tested species reactivity: mouse. Validated for flow cytometry.
- IgG2a, κ Isotype Control-PE, clone RTK2758 (Cat #400501). Tested species reactivity: mouse. Validated for flow cytometry.

Eukaryotic cell lines

Policy information about cell lines and Sex and Gender in Research

Cell line source(s)	MODE-K cells were purchased from Inserm-U1111 (Dr. Kaiserlian, Lyon, France)
Authentication	Original MODE-K cells were purchased from and verified by the original working group [Vidal, K., Grosjean, I., Evillard, J.P., Gespach, C. & Kaiserlian, D. J. Immunol. Methods. 166, 63-73 (1993)].
Mycoplasma contamination	MODE-K cells used were free of mycoplasma contamination.
Commonly misidentified lines (See ICLAC register)	No commonly misidentified cell lines were used in the study.

Animals and other research organisms

Policy information about studies involving animals; ARRIVE guidelines recommended for reporting animal research, and Sex and Gender in Research

Laboratory animals	For all the experiments, animals were age- and sex-matched. All animals were 8-14 weeks old. Both male and female were used.<br><br>Strains:<br>- C57BL/6J WT mice<br>- Swiss Webster mice<br>- Tlr2-/- global knockout mice, C57BL/6J background<br>- Tlr4-/- global knockout mice, C57BL/6J background<br>- Tlr5-/- global knockout mice, C57BL/6J background<br>- Tlr2-flox x VilCre, C57BL/6J background<br>- Nrp1-flox x VilCre, C57BL/6J background
Wild animals	No wild animals were used in the study.
Reporting on sex	All animals were sex-matched.
Field-collected samples	No field-collected samples were used in the study.
Ethics oversight	All procedures performed on mice were approved by the local committee on 27 legislation on protection of animals (Landesuntersuchungsamt Rheinland-Pfalz, Koblenz, Germany; 28 G12-1-035; G17-1-075; G20-1-119; A18-1-005).

Note that full information on the approval of the study protocol must also be provided in the manuscript.

Flow Cytometry

Plots

Confirm that:

☒ The axis labels state the marker and fluorochrome used (e.g. CD4-FITC).

☒ The axis scales are clearly visible. Include numbers along axes only for bottom left plot of group (a 'group' is an analysis of identical markers).

☒ All plots are contour plots with outliers or pseudocolor plots.

☒ A numerical value for number of cells or percentage (with statistics) is provided.

Methodology

Sample preparation	MODE-K cells were maintained in the medium at 37°C in a humidified atmosphere of 5% CO2.<br>For stimulation, MODE-K cells were seeded in 6-well plates until they reached 80–90 % confluence, following by treatment in cell culture medium for 2 h with 0.125% (v/v) bafilomycin A1, 0.1% (v/v) epoxomicin, 0.5 µM Pam3CSK4, or 2 µg/ml MALP-2. For the vehicle, the equivalent amount of DMSO (0.1-0.125%) was used.<br><br>For flow cytometry analysis, cells were re-suspended in PBS added with 3% (v/v) FCS (FACS buffer).<br>Cells were pre-incubated with 1:100 (v/v) CD16/32 TruStain FcX mAb for 10 min on ice, pelleted and washed in FACS buffer. Then, MODE-K cells were incubated with the fluorescent Abs (i.e. EpCAM-PerCP, NRP1-PE or alternatively IgG2A, k isotype control-PE) for 30 min at 4 degrees in the dark. After pelleting and washing, cells were re-suspended in PBS and analysed by flow cytometry.
Instrument	BD FACSCanto II instrument<br>Model #338962 (3 Lasers, 8 colors)
Software	Data visualization: BD FACSDiva Software<br>Data analysis: FlowJo Software
Cell population abundance	N/A: cells were not sorted.
Gating strategy	1) MODE-K cells were visualized on a FSC-A vs SSC-A gate, to exclude debris and apoptotic cells. Population of single cells was selected.<br>2) Epithelial cells (EPCAM+) expressing NRP1 (NPR1+) were selected with an isotype control.<br>3) With the same isotype control, living cells (i.e., DAPI-) were selected.

☒ Tick this box to confirm that a figure exemplifying the gating strategy is provided in the Supplementary Information.
