# Supplementary material for: Commensal bacteria weaken the intestinal barrier by suppressing epithelial neuropilin-1 and Hedgehog signaling
Source: Nat Metab. 2023 Jul 6;5(7):1174–87. doi: 10.1038/s42255-023-00828-5 (PMC10365997; doi:10.1038/s42255-023-00828-5)
Supplement: Supplementary file 2 — qPCR primer nucleotide sequences. [file 42255_2023_828_MOESM2_ESM.pdf]

Supplementary Table 1. qPCR primer nucleotide sequences

Gene Name	Primers	5' – 3' Nucleotide Sequences
Bone Morphogenetic Protein-4	*Bmp4* for	TTCCTGGTAACCGAATGCTGA
	Bmp4 rev	CCTGAATCTCGGCGACTTTTT
Claudin-4	*Cldn4* for	TGGAGGACGAGACCGTCAA
	Cldn4 rev	CACGGGCACCATAATCAGCA
Junctional Adhesion Molecule-A	*F11r* for	AGTGGAAGTTCGTCCAAGGC
	F11r rev	ACTCGGTCCGCATAGGGAG
Glioma-Associated Oncogene-1	*Gli1* for	TACCATGAGCCCTTCTTTAGGA
	Gli1 rev	GCATCATTGAACCCCGAGTAG
Hedgehog Interacting Protein	*Hhip* for	CAGGTCTTCTTCAAACAACTGCT
	Hhip rev	TGCTTTCTCGGGAAGTCTGGA
Indian Hedgehog	*Ihh* for	GACGAGGAGAACACGGGTG
	Ihh rev	GCGGCCCTCATAGTGTAAAGA
60S Ribosomal Protein L32	*L32* for	CCTCTGGTGAAGCCCAAGATC
	L32 rev	TCTGGGTTTCCGCCAGTTT
Neuropilin-1	*Nrp1* for	GACAAATGTGGCGGGACCATA
	Nrp1 rev	TGGATTAGCCATTCACACTTCTC
Occludin	*Ocln* for	TTGAAAGTCCACCTCCTTACAGA
	Ocln rev	CCGGATAAAAAGAGTACGCTGG
Platelet and Endothelial Cell Adhesion Molecule-1	*Pecam1* for	CTTCATCCACTGGGGCTATC
	Pecam1 rev	CTGCCAGTCCGAAAATGGAAC
Patched Homolog-1	*Ptch1* for	GCCTTCGCTGTGGGATTAAAG
	Ptch1 rev	CTTCTCCTATCTTCTGACGGGT
Zonula Occludens-1	*Tjp1* for	GAGCGGGCTACCTTACTGAAC
	Tjp1 rev	GTCATCTCTTTCCGAGGCATTAG
Toll-like Receptor-2	*Tlr2* for	ACAATAGAGGGAGACGCCTTT
	Tlr2 rev	AGTGTCTGGTAAGGATTTCCCAT
Semaphorin-3A	*Sema3A for*	ACTGGGGCTTTCCATCCAATC
	Sema3A rev	CTCTTCCCACGACCGTTTTCA
