# Supplementary figures and images for: Commensal bacteria weaken the intestinal barrier by suppressing epithelial neuropilin-1 and Hedgehog signaling
Source: Nat Metab. 2023 Jul 6;5(7):1174–87. doi: 10.1038/s42255-023-00828-5 (PMC10365997; doi:10.1038/s42255-023-00828-5)

Figure 1a

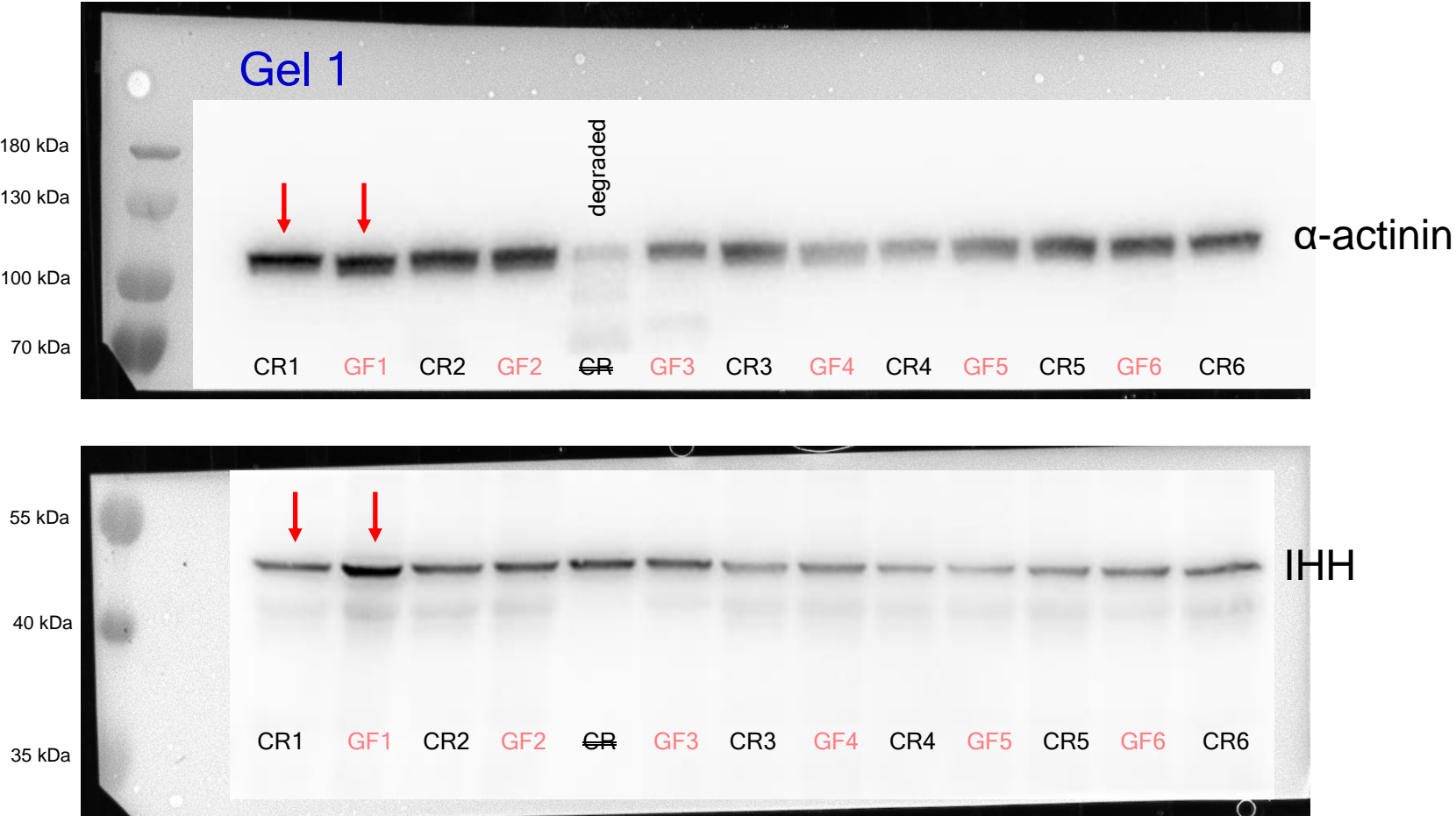

Supplement: Source Data Fig. 1 — Unprocessed western blots for Fig. 1. [file 42255_2023_828_MOESM4_ESM.pdf]

Figure 2b

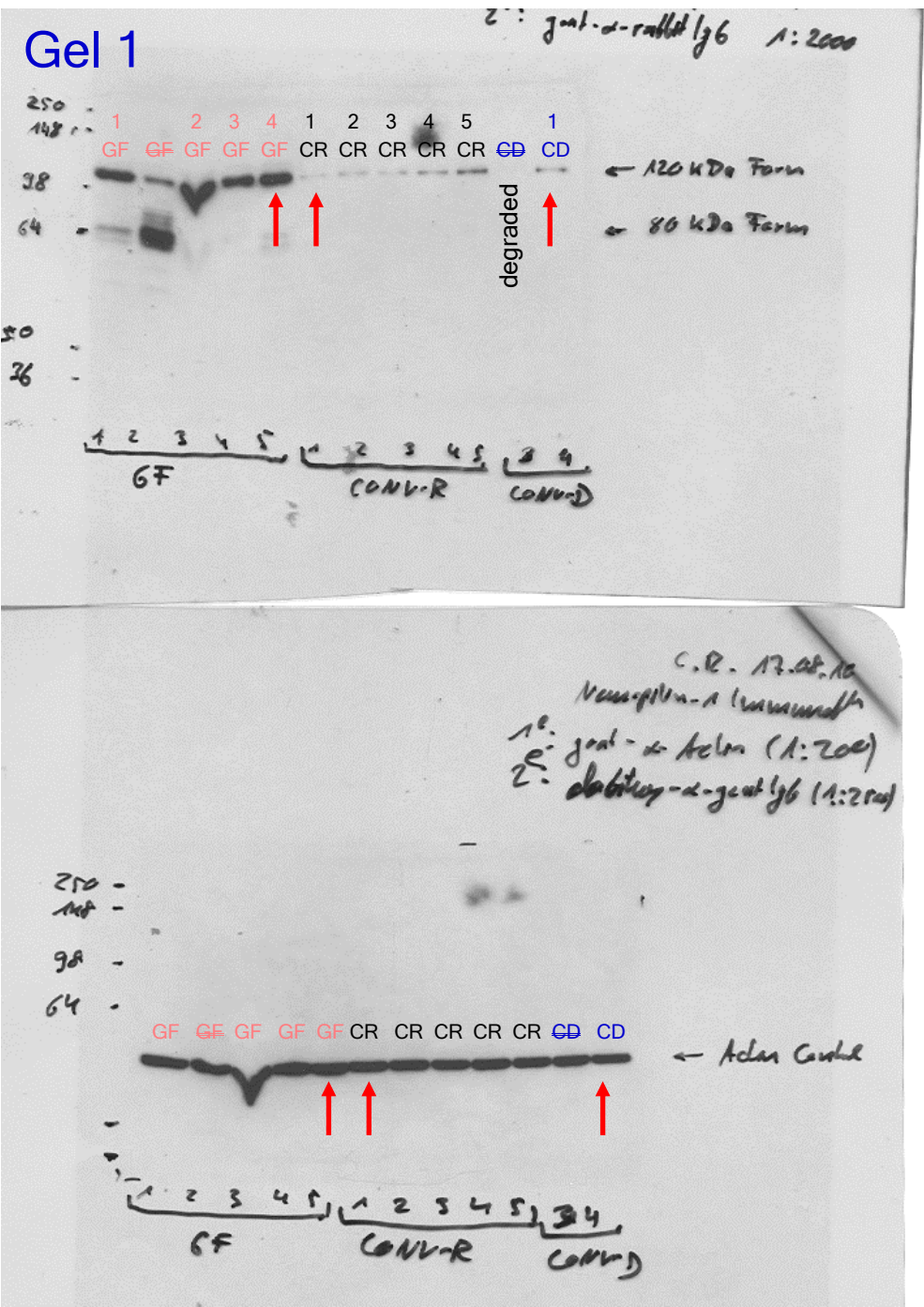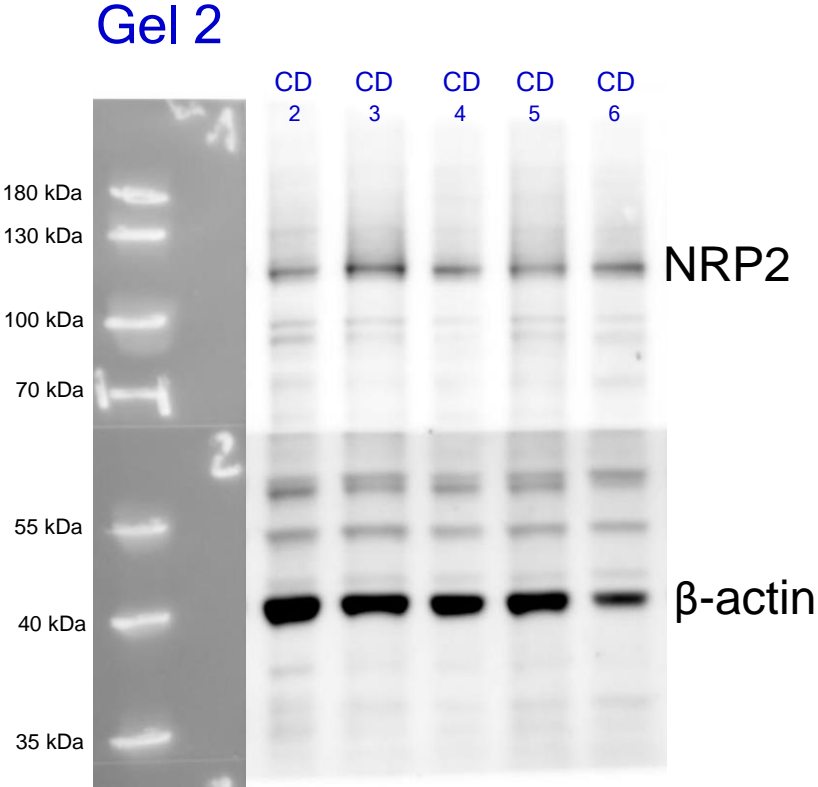

Figure 2c

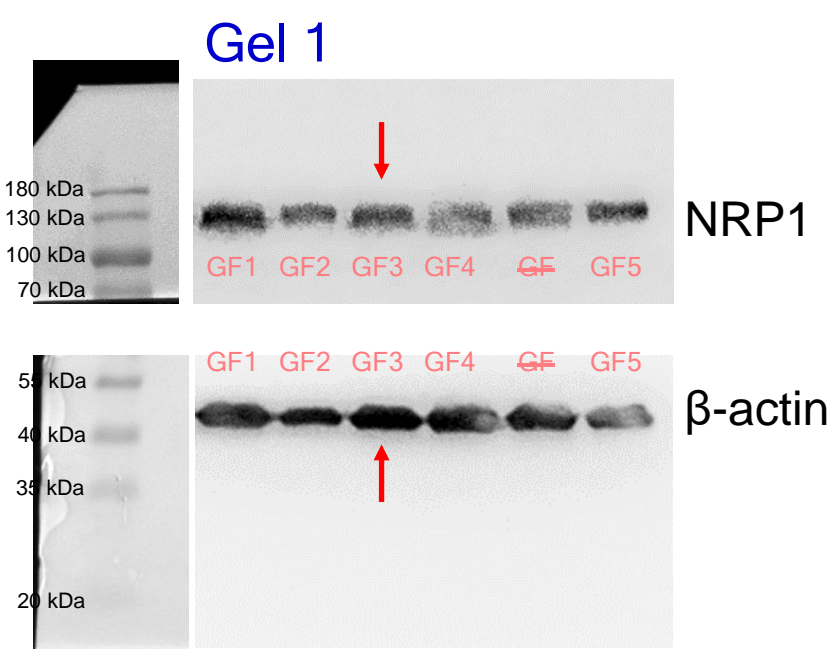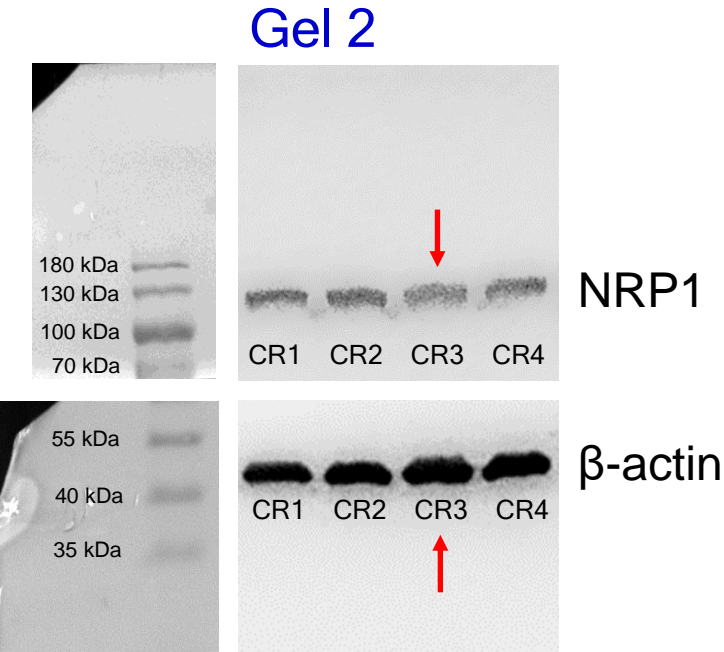

Figure 2e

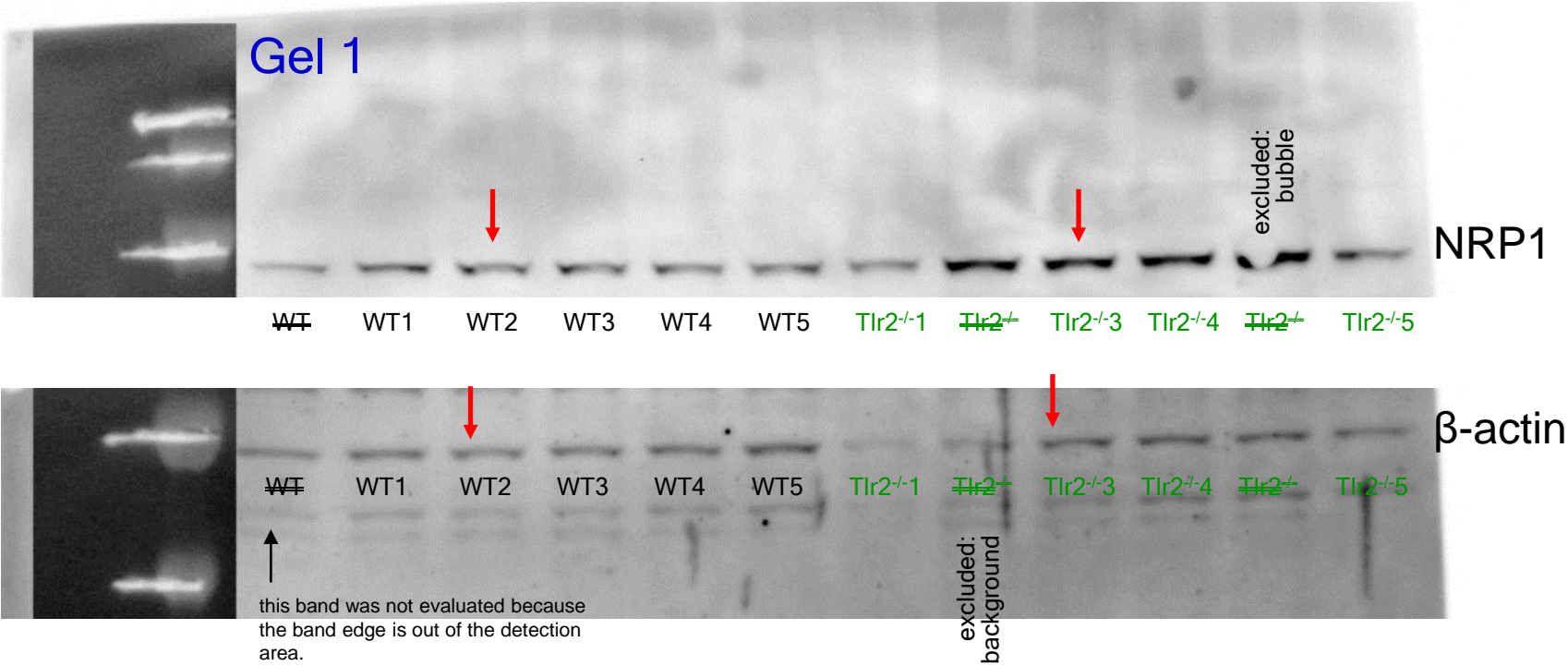

Figure 2f

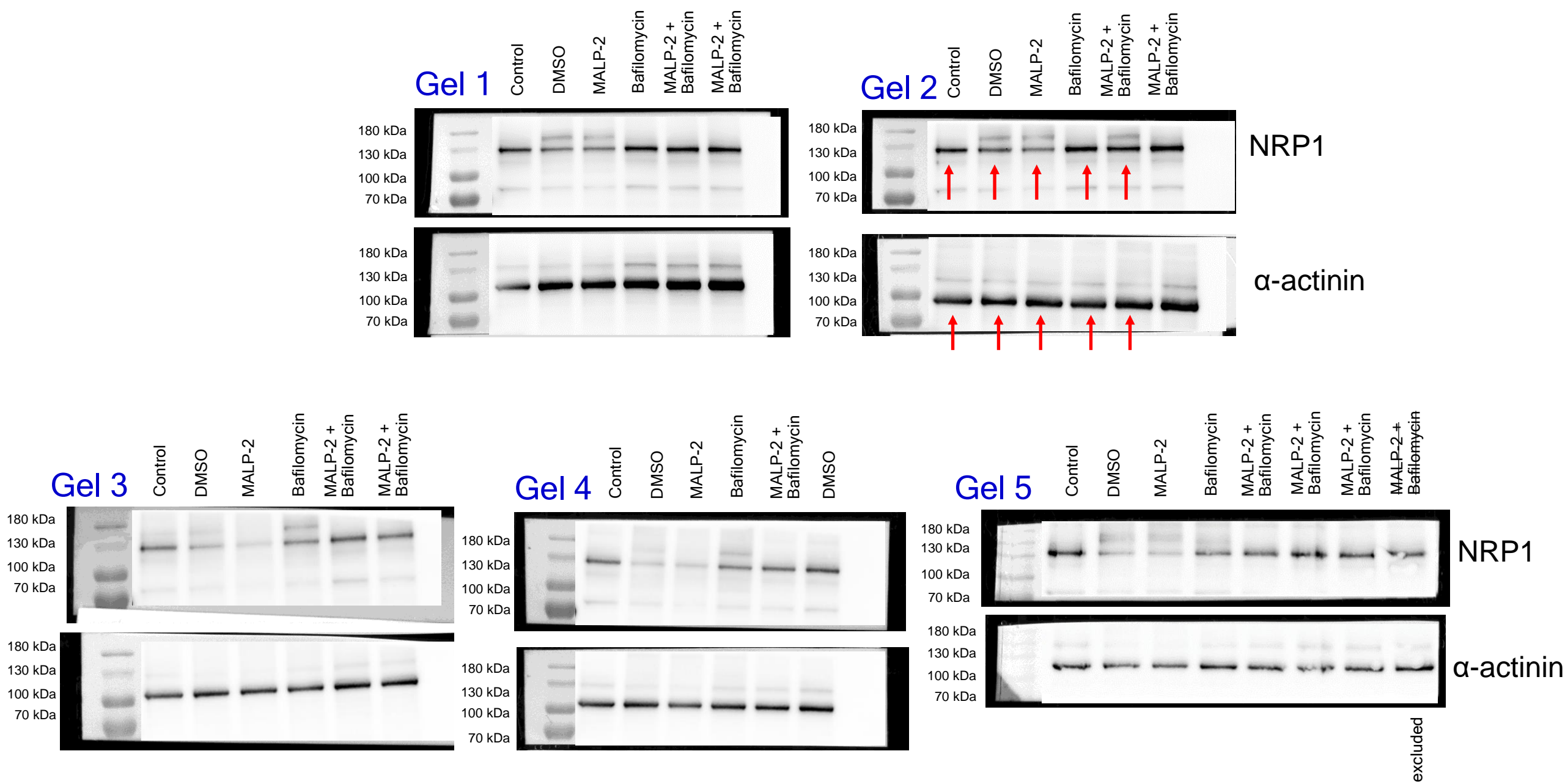

Figure 2f

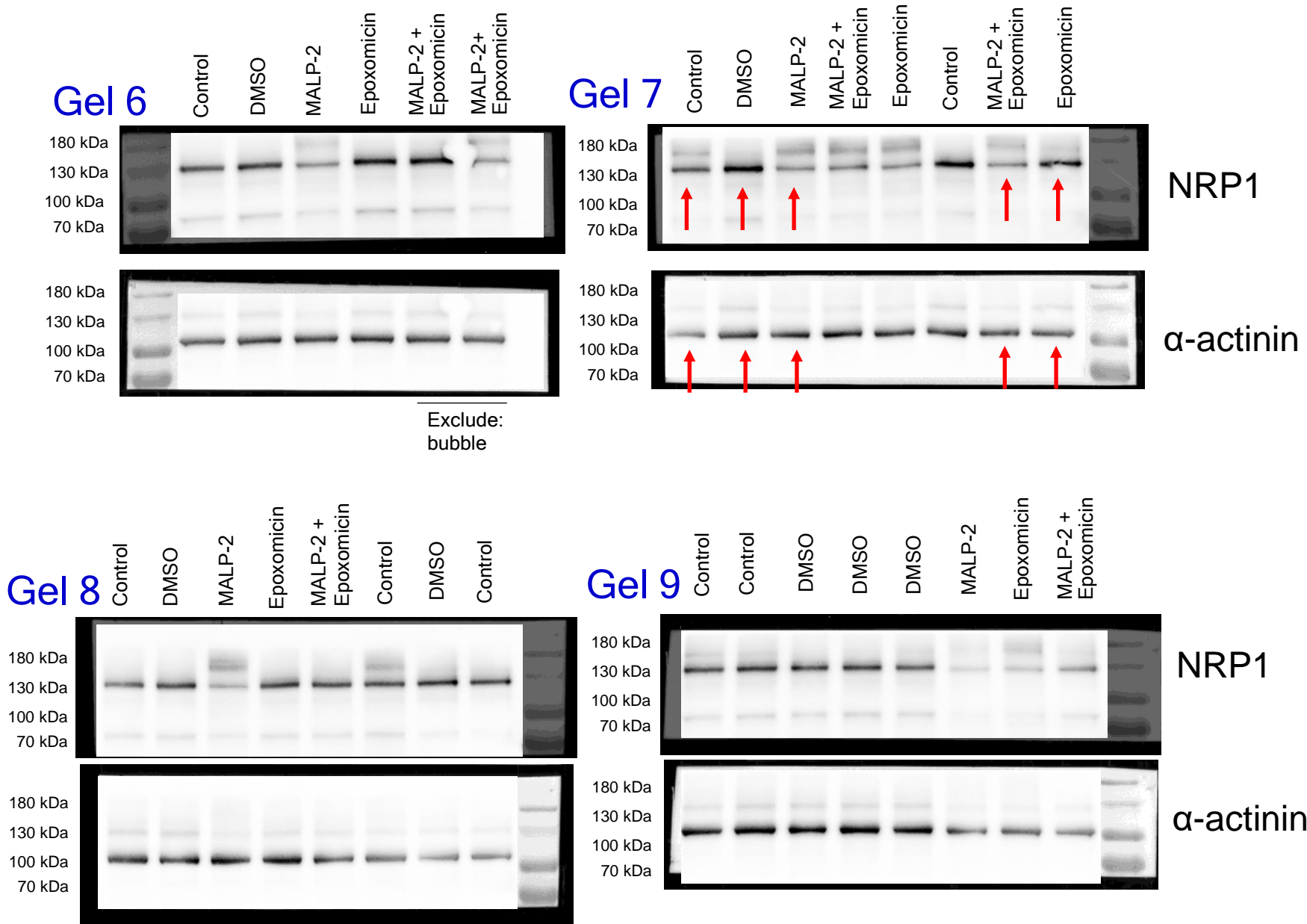

Supplement: Source Data Fig. 2 — Unprocessed western blots for Fig. 2. [file 42255_2023_828_MOESM7_ESM.pdf]

Figure 2a

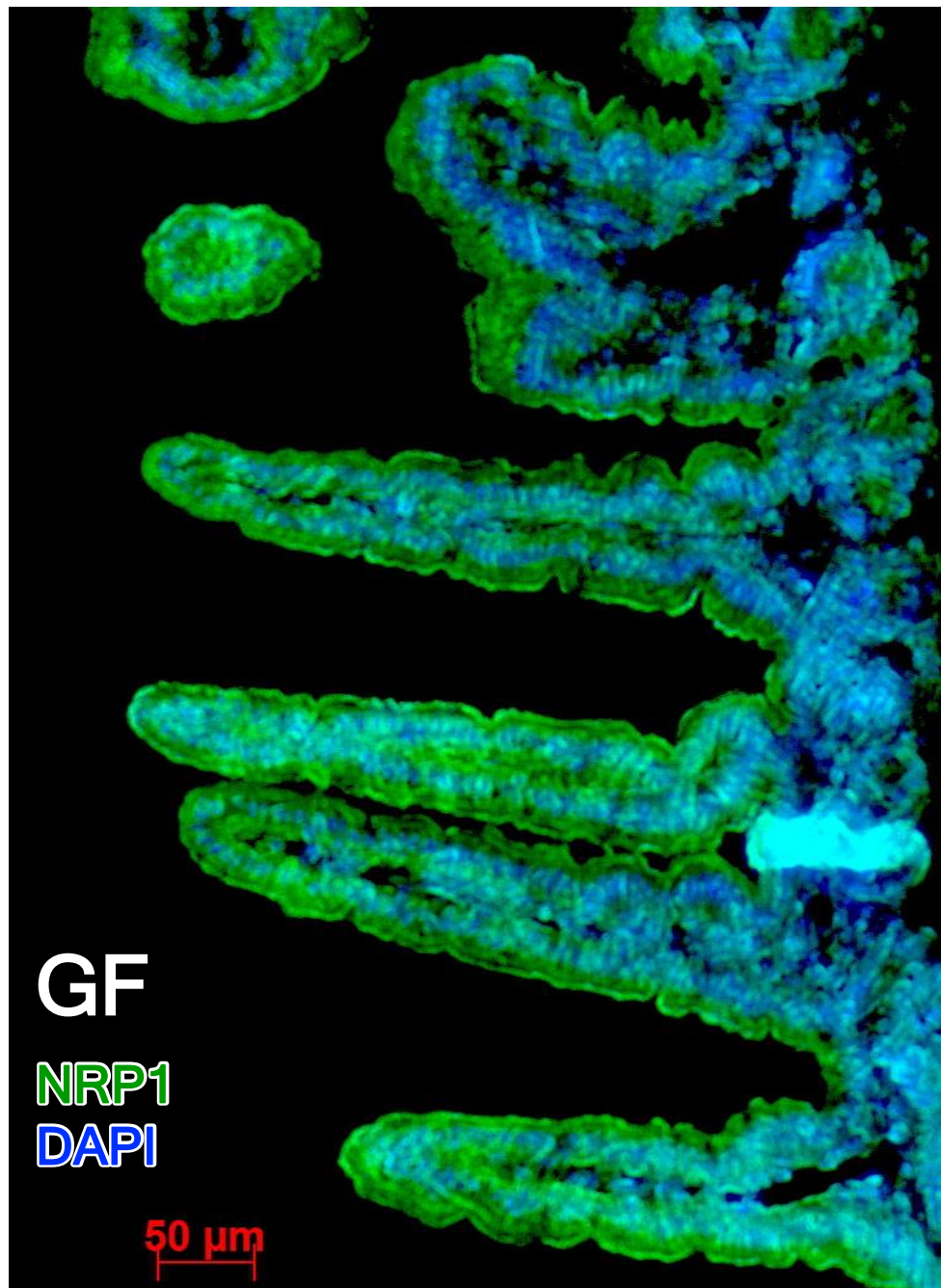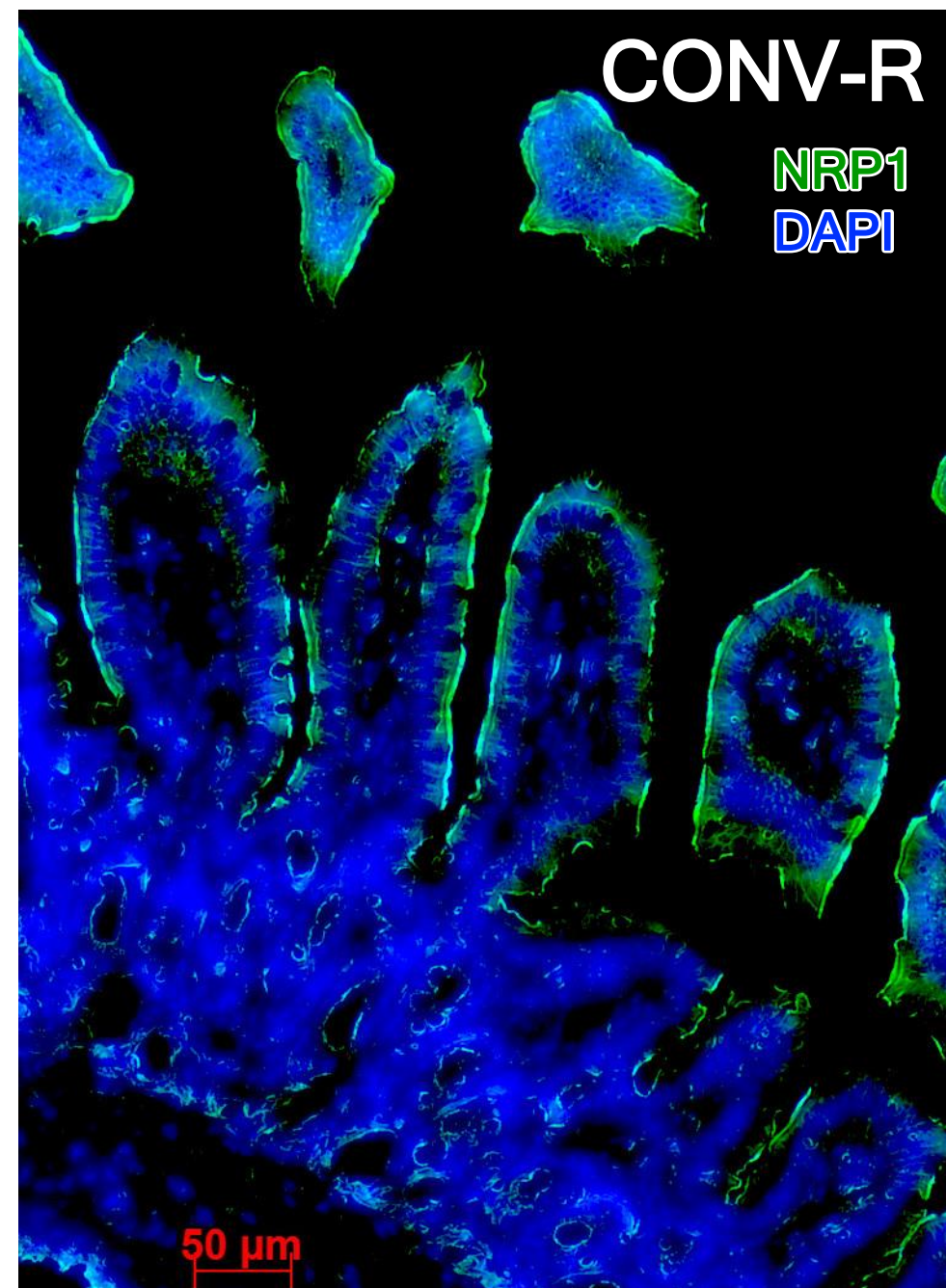

Supplement: Source Data Fig. 2 — Micrographs for Fig. 2. [file 42255_2023_828_MOESM8_ESM.pdf]

Figure 4h

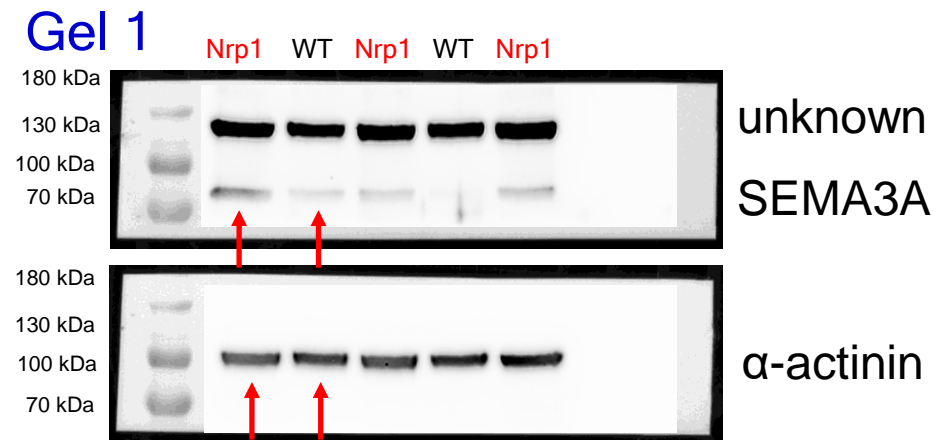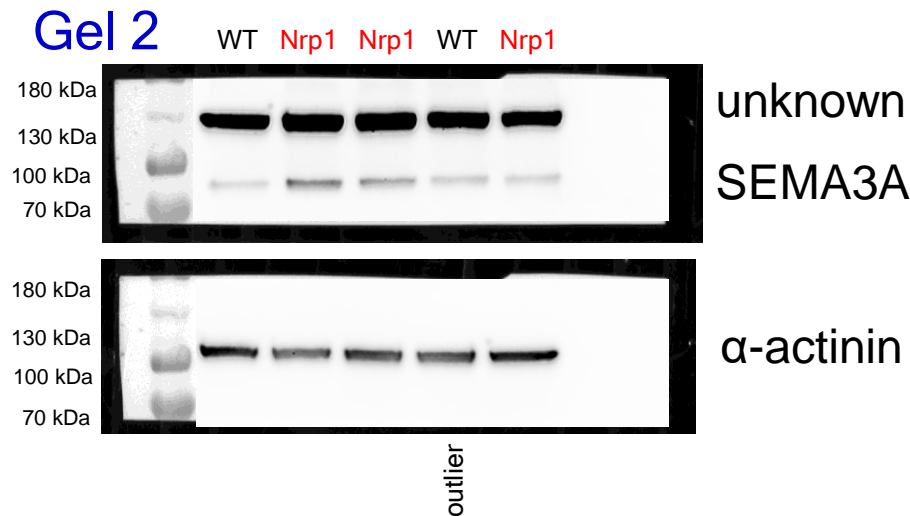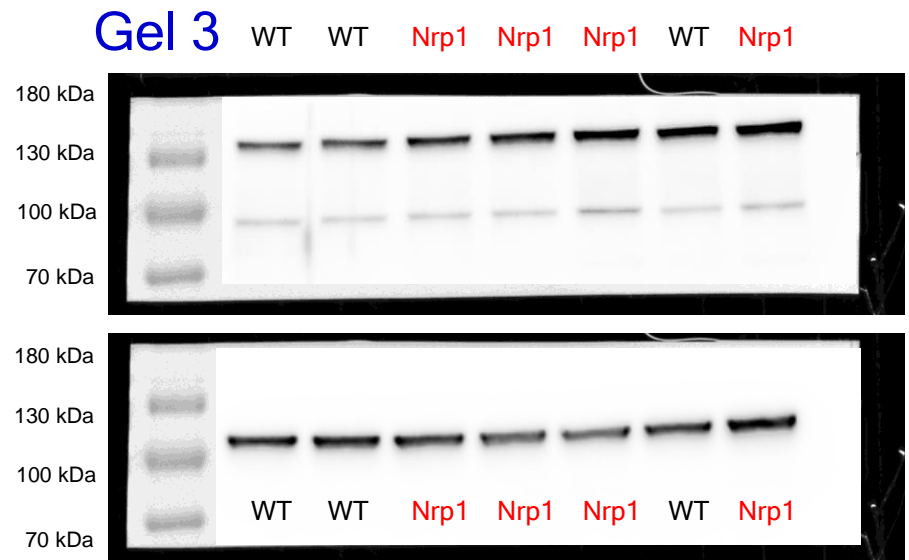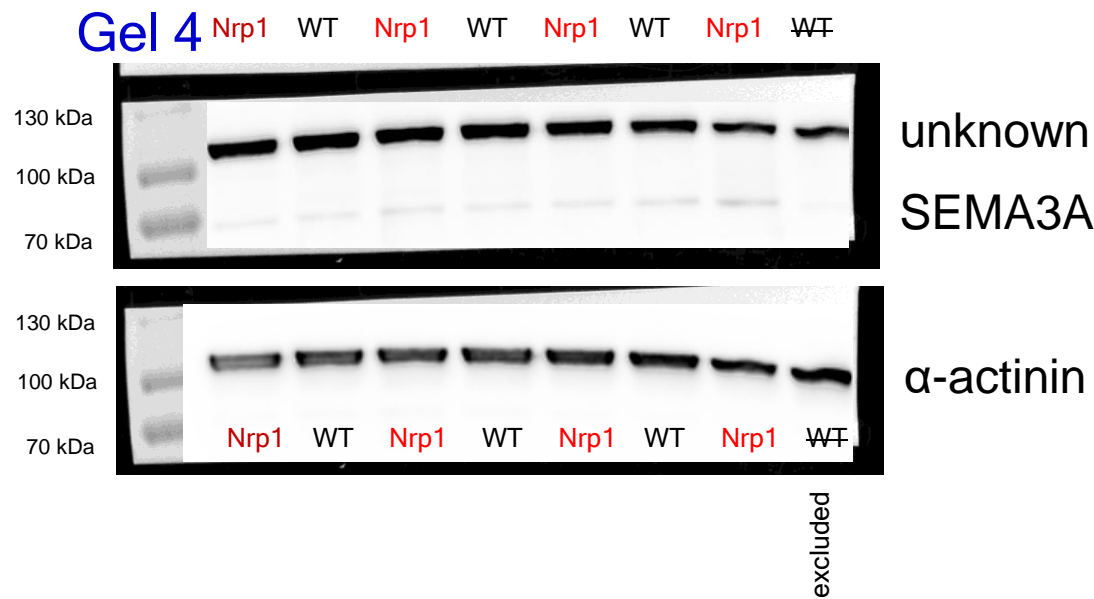

Supplement: Source Data Fig. 4 — Unprocessed western blots for Fig. 4. [file 42255_2023_828_MOESM12_ESM.pdf]

Extended Data Figure 2b

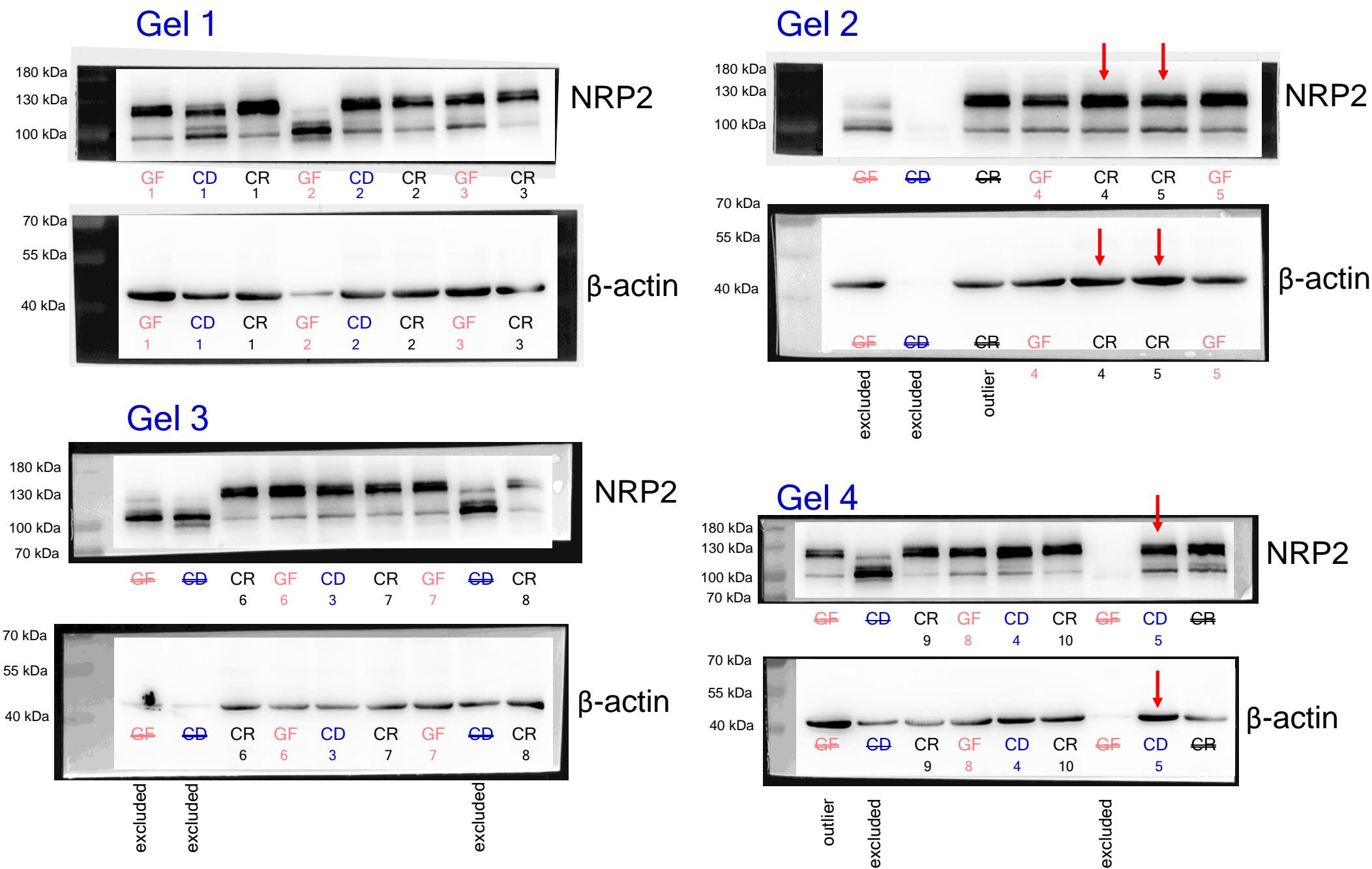

Extended Data Figure 2c

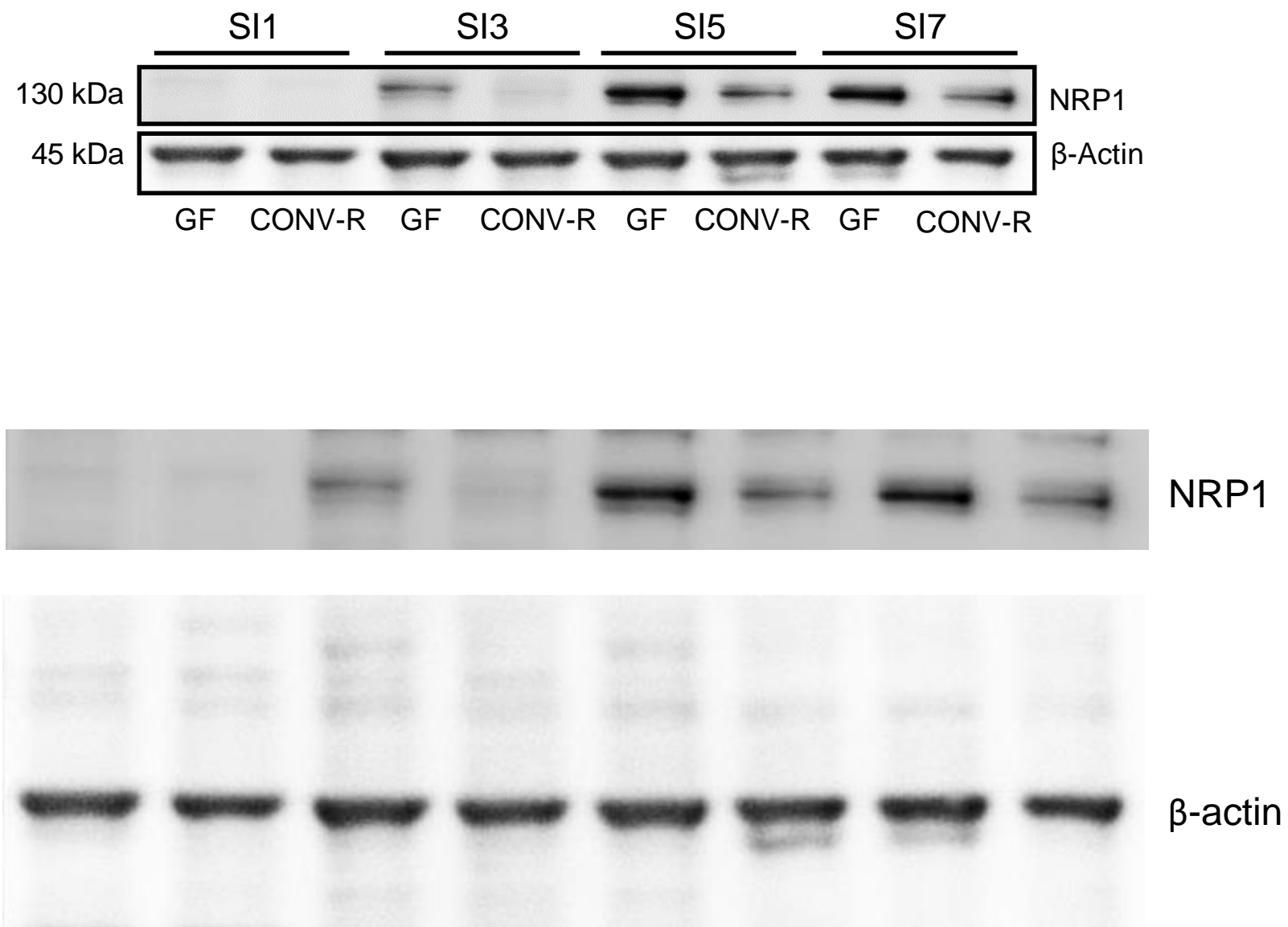

Extended Data Figure 2d

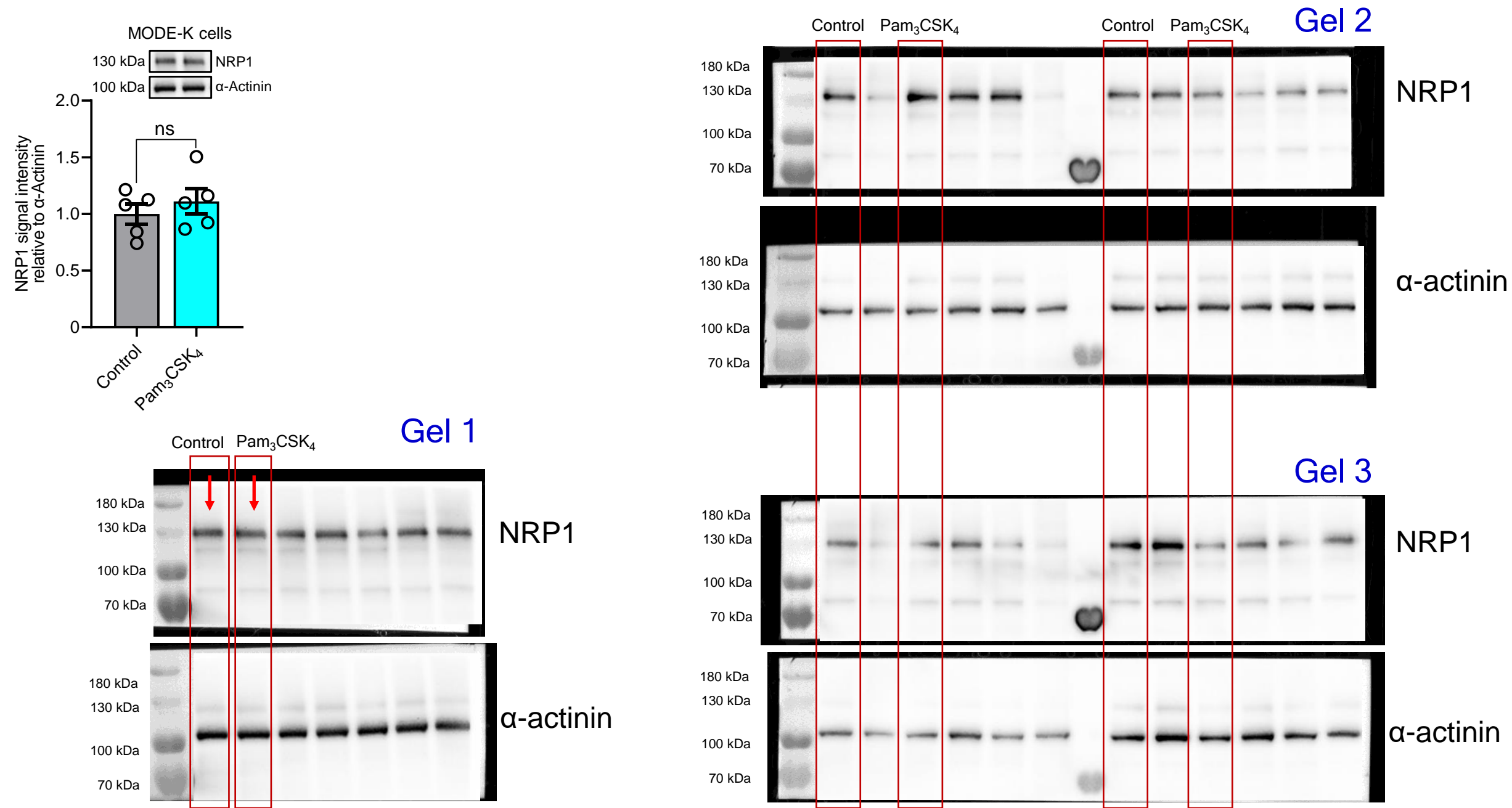

Supplement: Source Data Extended Data Fig. 2 — Unprocessed western blots for Extended Data Fig. 2. [file 42255_2023_828_MOESM16_ESM.pdf]

Extended Data Figure 2a

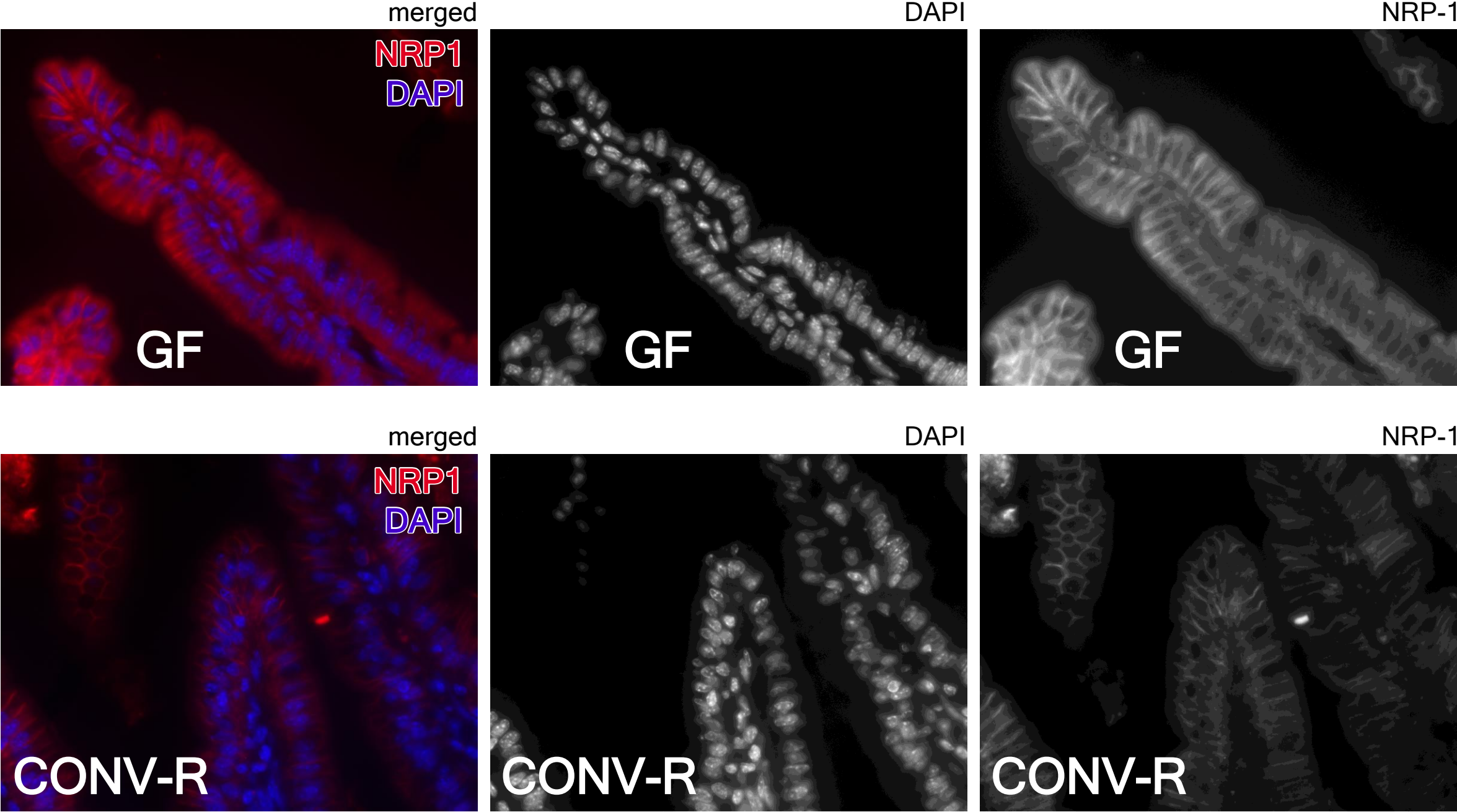

Supplement: Source Data Extended Data Fig. 2 — Micrographs for Extended Data Fig. 2. [file 42255_2023_828_MOESM17_ESM.pdf]
