# Supplementary material for: Commensal bacteria weaken the intestinal barrier by suppressing epithelial neuropilin-1 and Hedgehog signaling
Source: Nat Metab. 2023 Jul 6;5(7):1174–87. doi: 10.1038/s42255-023-00828-5 (PMC10365997; doi:10.1038/s42255-023-00828-5)

Figure 1j

Representative images and quantification were taken from GF mouse 1.

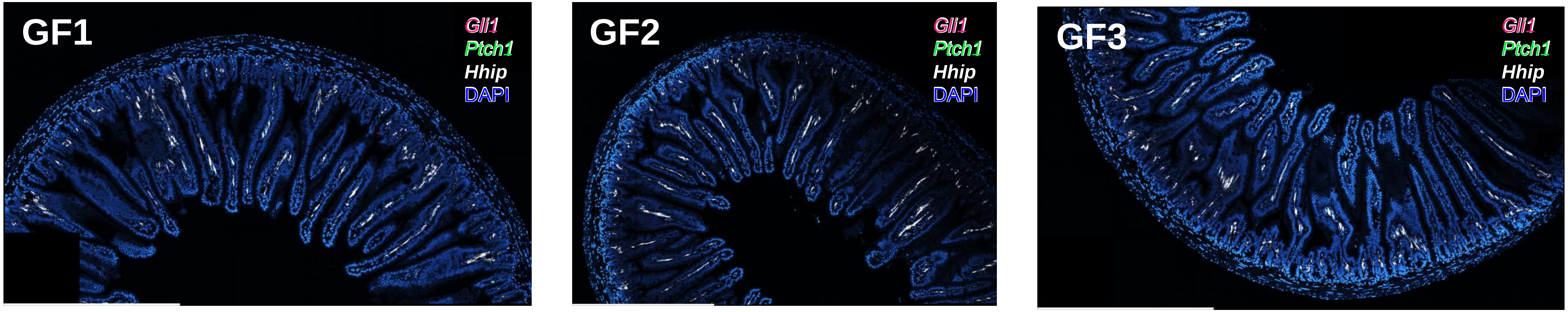

Representative images and quantification were taken from CONV-R mouse 1.

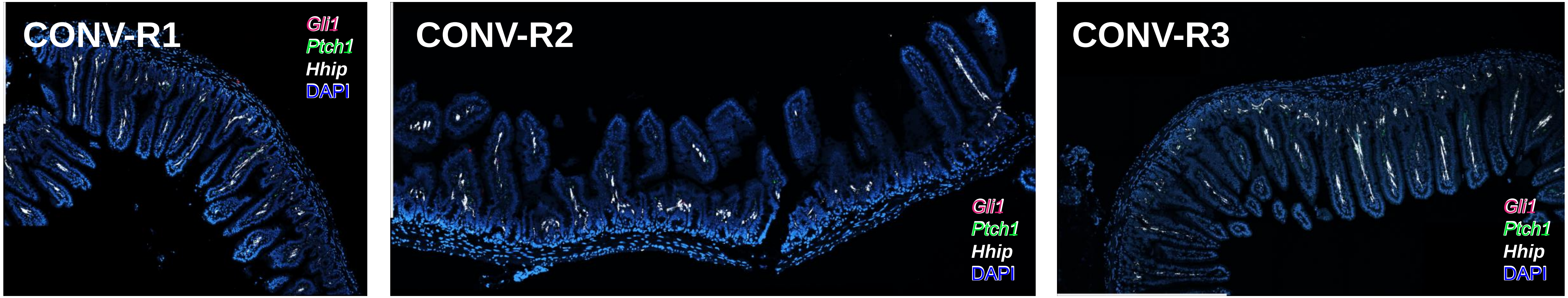

Figure 1k

Representative images and quantification were taken from WT mouse 3.

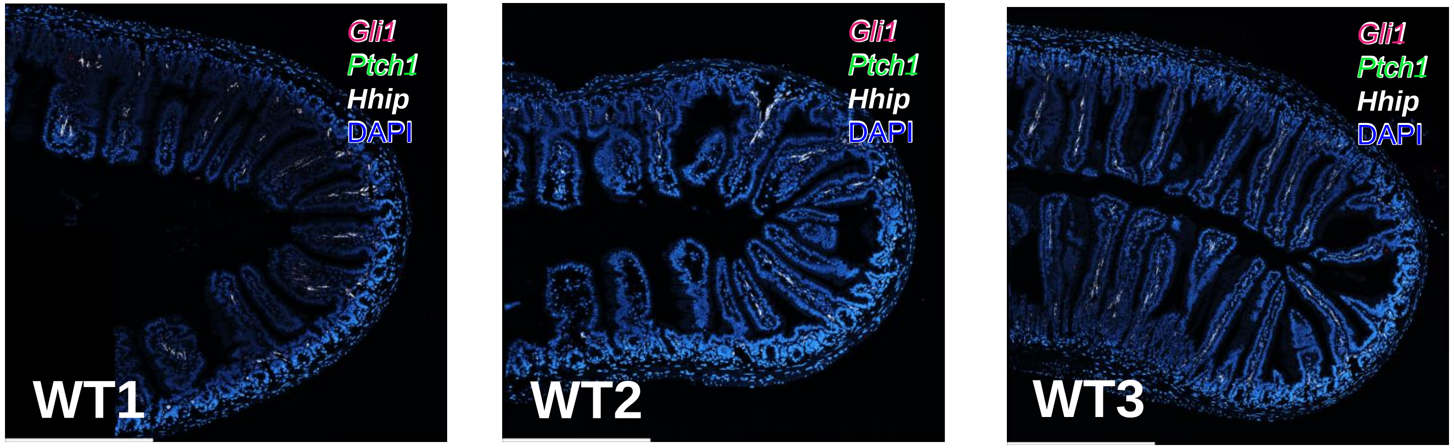

Representative images and quantification were taken from *Tlr2*$^{\Delta IEC}$ mouse 3.

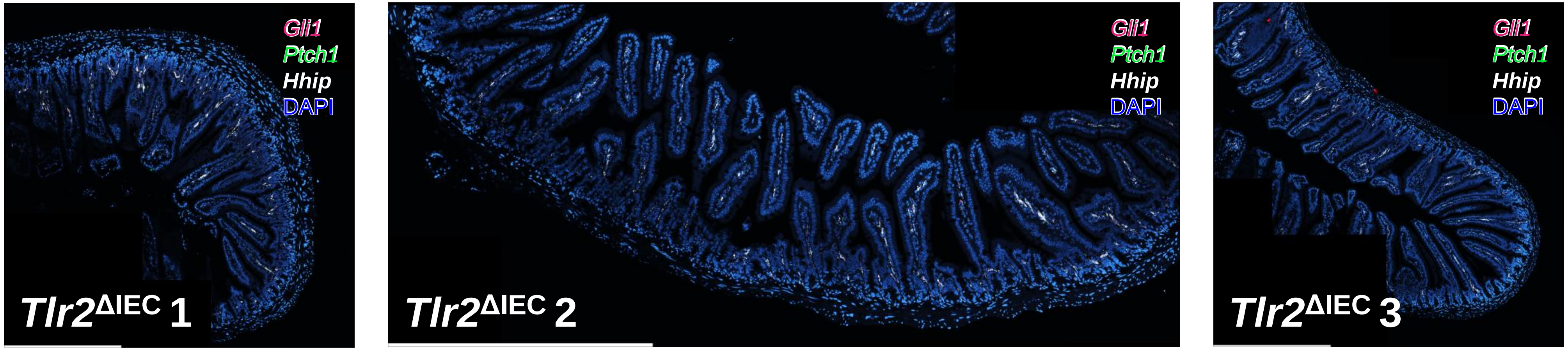

Supplement: Source Data Fig. 1 — Micrographs for Fig. 1. [file 42255_2023_828_MOESM5_ESM.pdf]
