# Supplementary material for: Commensal bacteria weaken the intestinal barrier by suppressing epithelial neuropilin-1 and Hedgehog signaling
Source: Nat Metab. 2023 Jul 6;5(7):1174–87. doi: 10.1038/s42255-023-00828-5 (PMC10365997; doi:10.1038/s42255-023-00828-5)
Supplement: Source Data Fig. 3 — Unprocessed western blots for Fig. 3. [file 42255_2023_828_MOESM10_ESM.pdf]

Figure 3c - part I

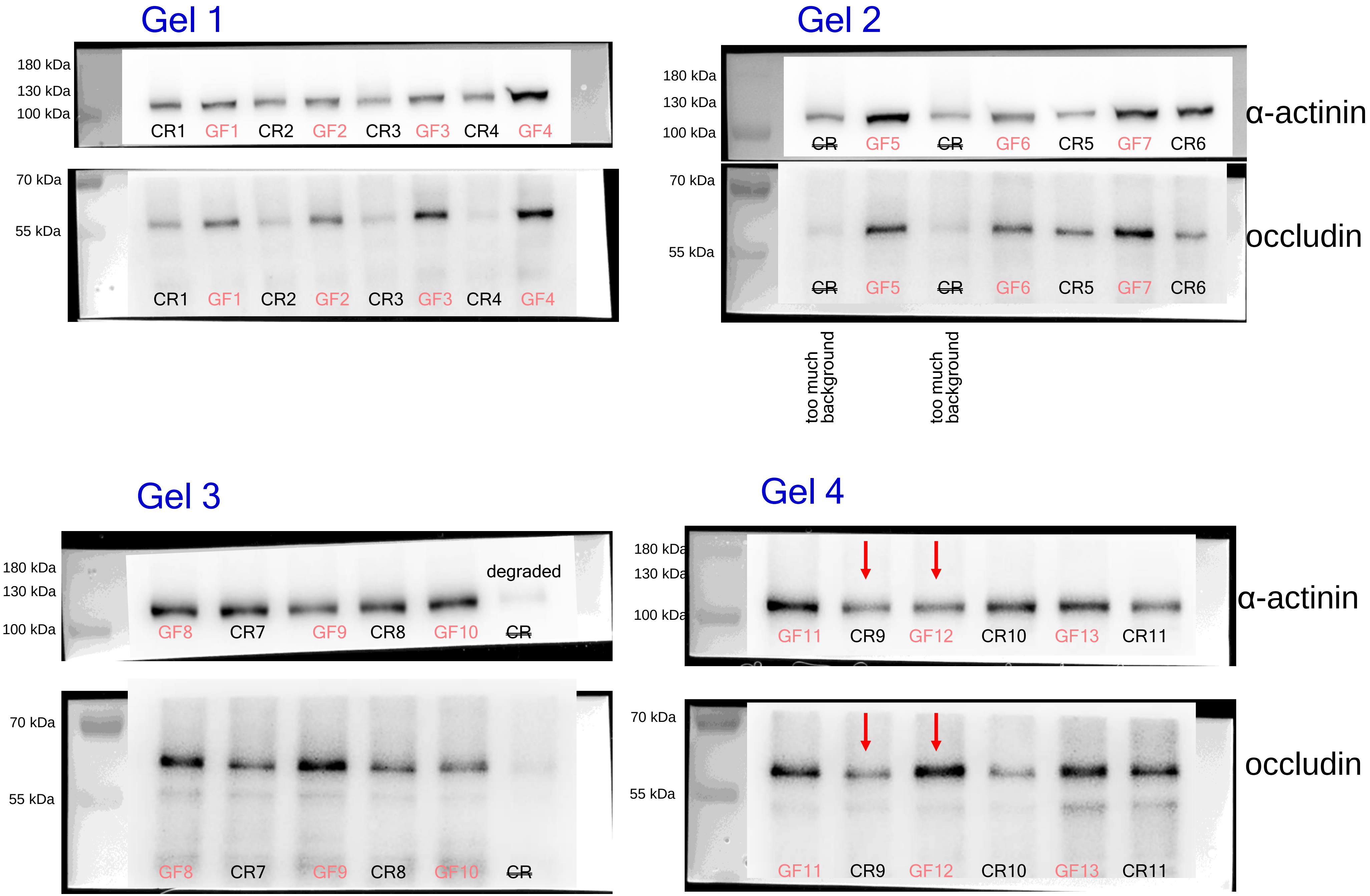

Figure 3c – part II

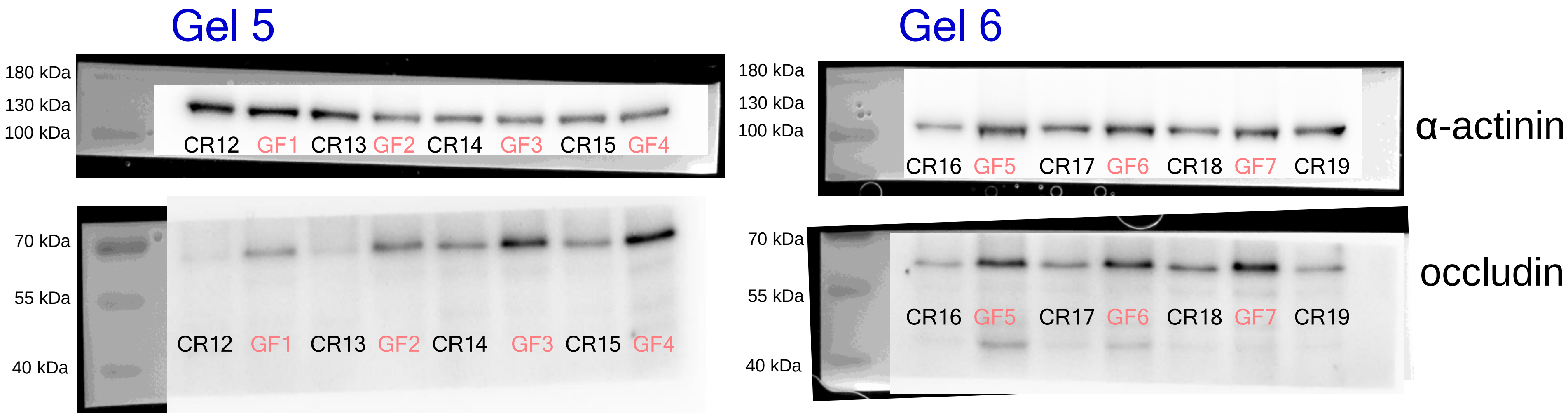

these GF mice are excluded from the graphs
because they are the same biological samples of the Gel 1 and 2.

They were included only to normalize the values of CONV-R mice.

Figure 3d

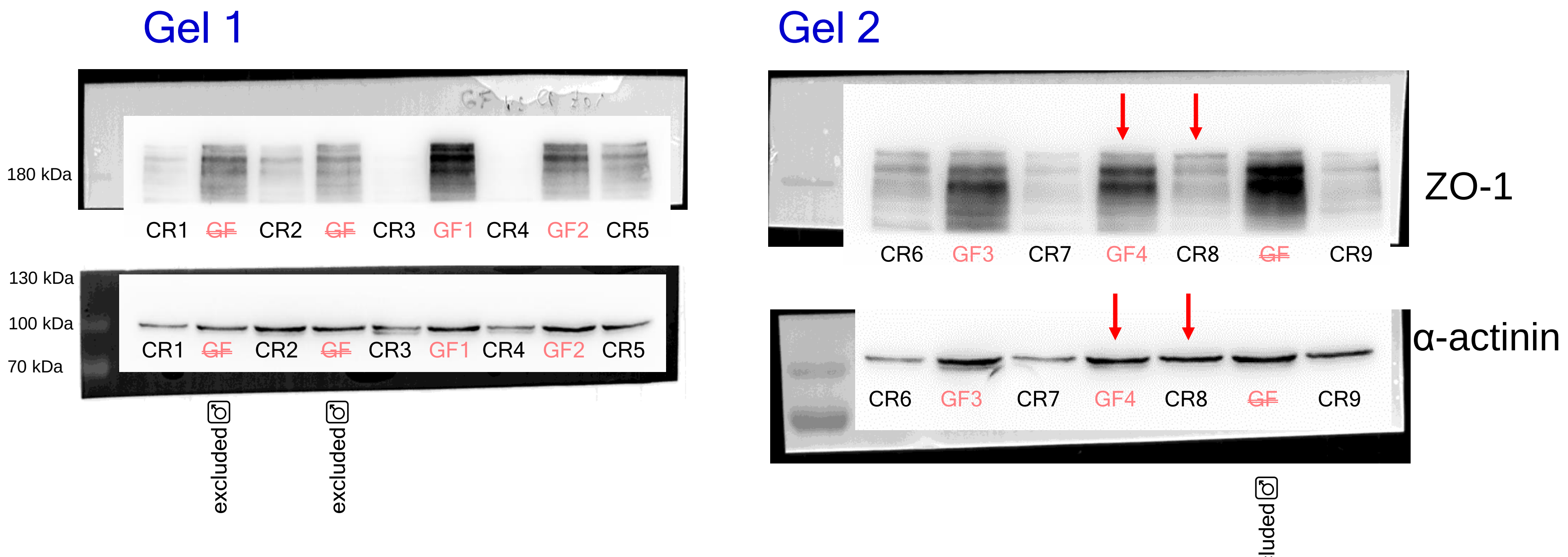

Since all the CONV-R mice are females, to have a sex-balanced group, GF male mice were excluded.

Figure 3k

Gel 1

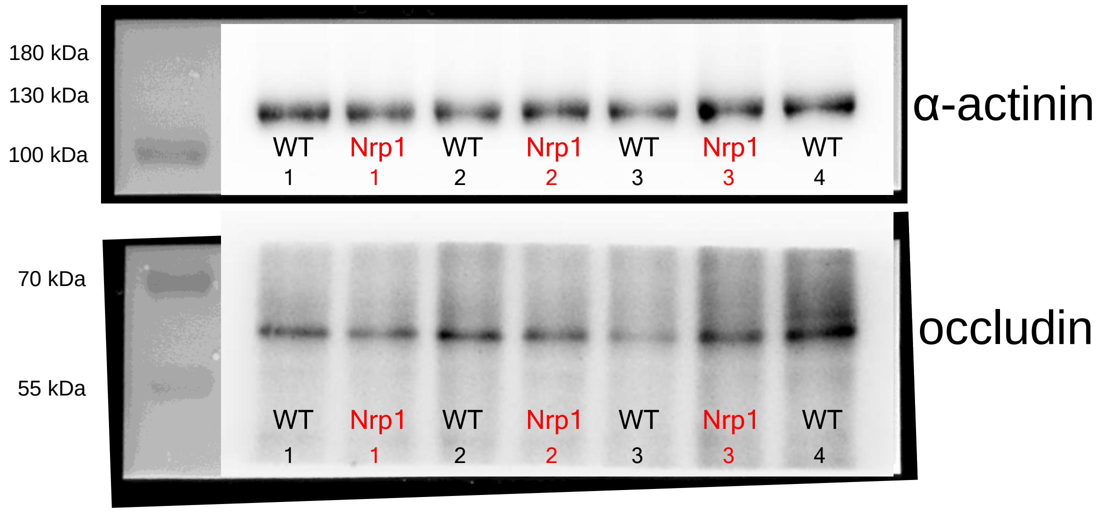

Gel 2

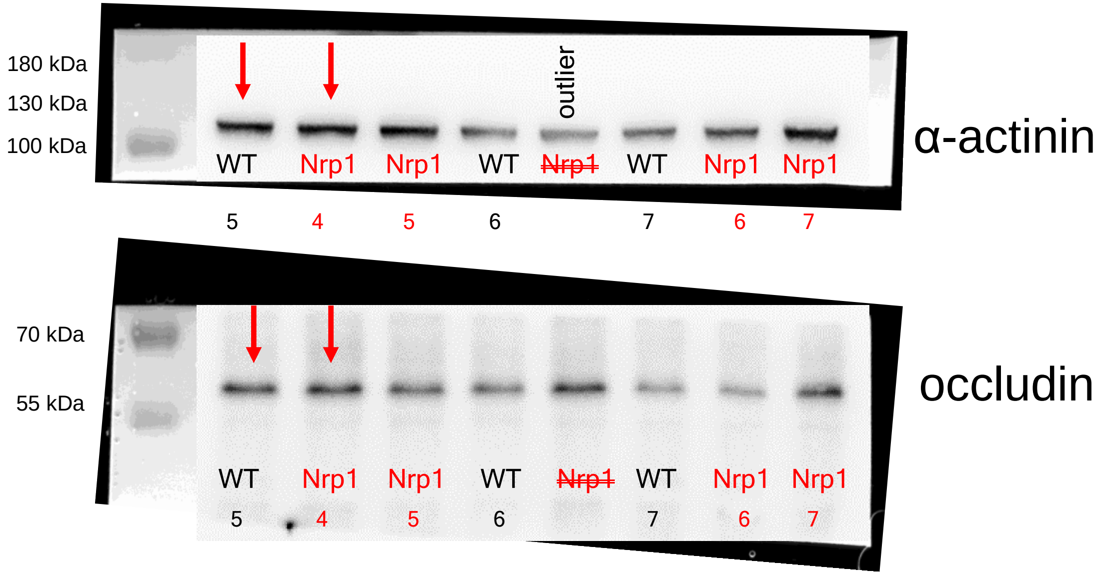

Since ZO-1 and occludin have different MW, the same gel was used for Figure 3k (Gel2, occludin) and Figure 3l (Gel2, ZO-1)

Figure 3l

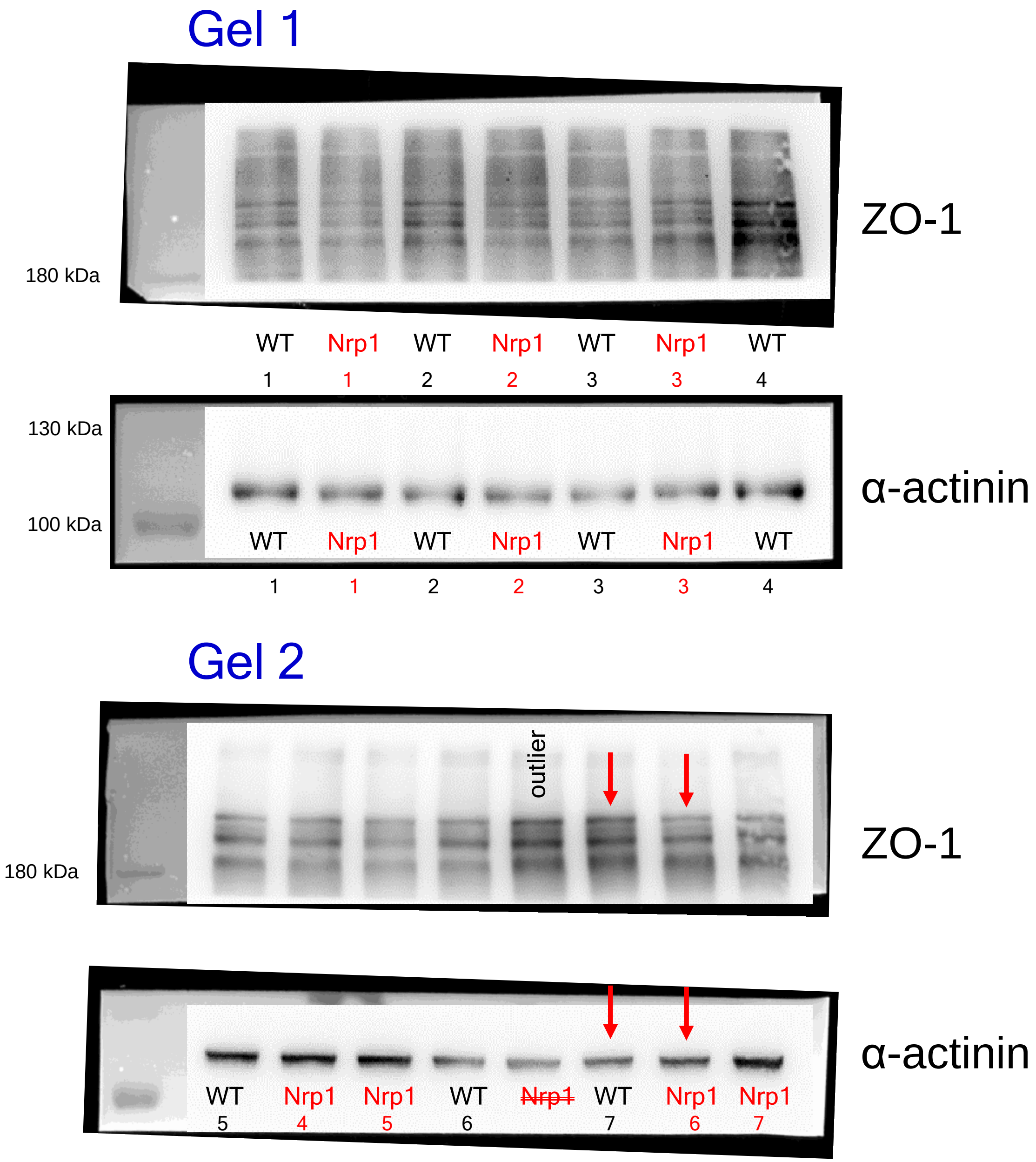

Since ZO-1 and occludin have different MW, the same gel was used for Figure 3k (Gel2, occludin) and Figure 3l (Gel2, ZO-1)

Figure 3o

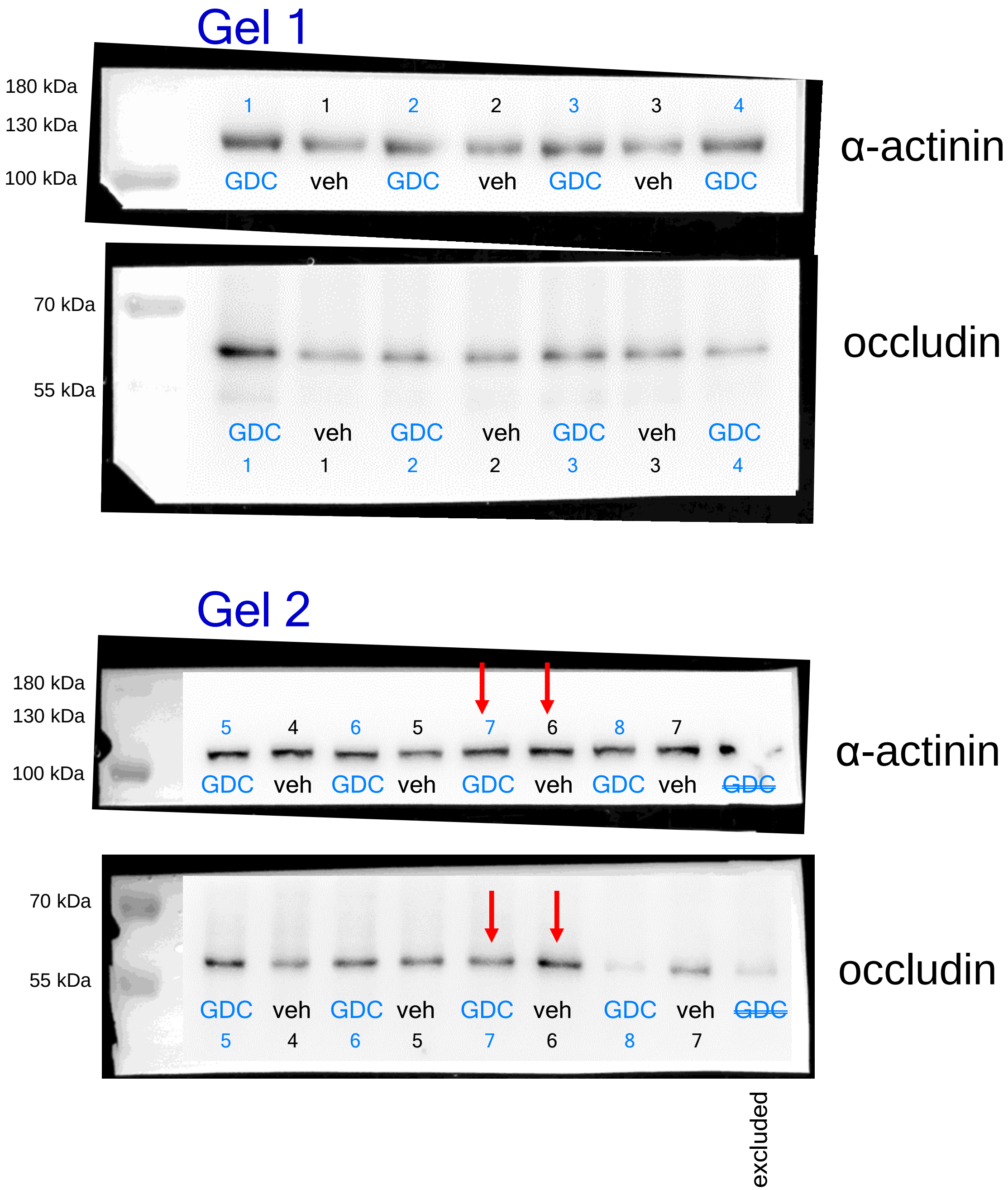

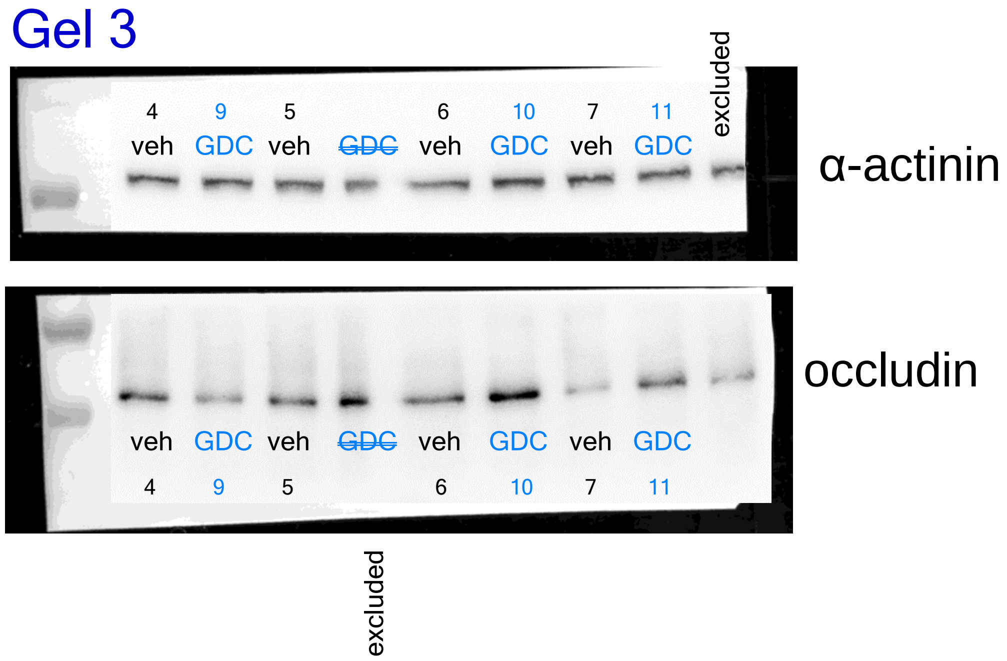

The vehicle from Gel 3 are the same as Gel 2.
Therefore, they are used only for normalization
and not in the quantification.

Figure 3p

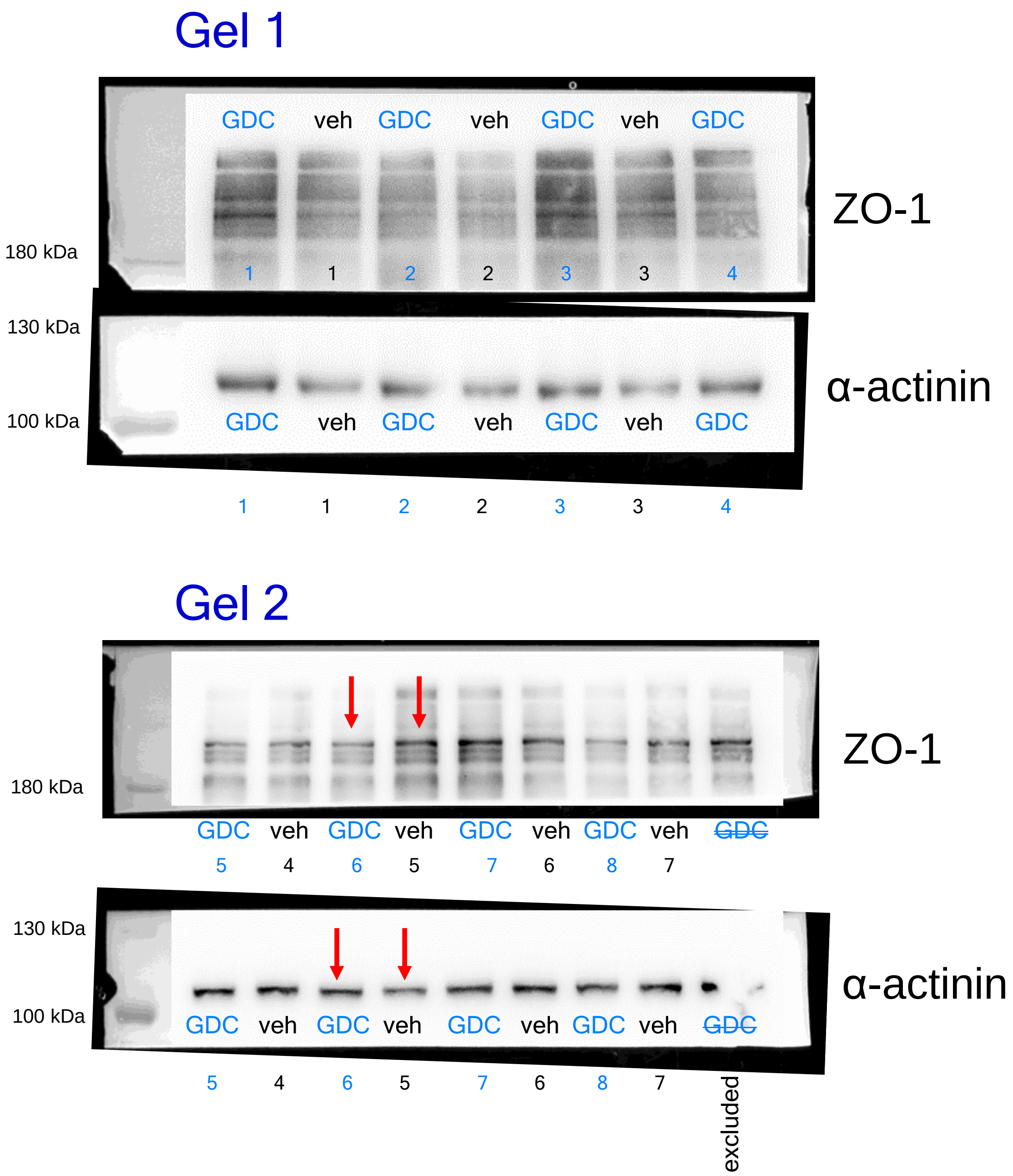

Gel 1
GDC veh GDC veh GDC veh GDC
ZO-1
1 1 2 2 3 3 4
180 kDa
130 kDa
100 kDa
GDC veh GDC veh GDC veh GDC
α-actinin
1 1 2 2 3 3 4
Gel 2
ZO-1
180 kDa
GDC veh GDC veh GDC veh GDC veh GDC
5 4 6 5 7 6 8 7
130 kDa
α-actinin
100 kDa
GDC veh GDC veh GDC veh GDC veh GDC
5 4 6 5 7 6 8 7
excluded
