# Supplementary material for: Commensal bacteria weaken the intestinal barrier by suppressing epithelial neuropilin-1 and Hedgehog signaling
Source: Nat Metab. 2023 Jul 6;5(7):1174–87. doi: 10.1038/s42255-023-00828-5 (PMC10365997; doi:10.1038/s42255-023-00828-5)
Supplement: Source Data Fig. 4 — Micrographs for Fig. 4. [file 42255_2023_828_MOESM13_ESM.pdf]

Figure 4a - b

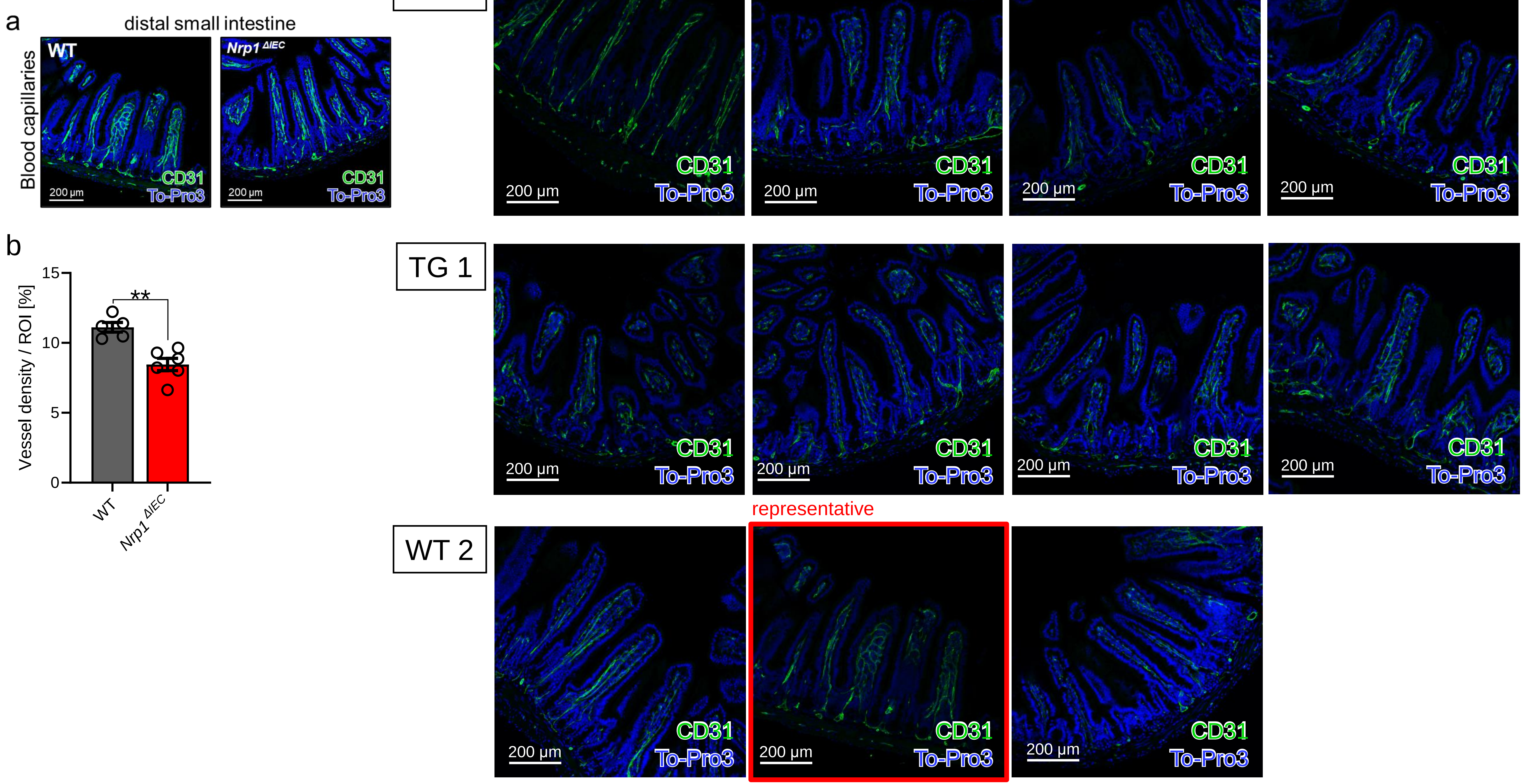
a
distal small intestine
Blood capillaries
WT
Nrp1 ΔIEC
CD31
To-Pro3
200 µm
b
Vessel density / ROI [%]
15
10
5
0
**
WT
Nrp1 ΔIEC
WT 1
TG 1
WT 2
representative

Figure 4a - b

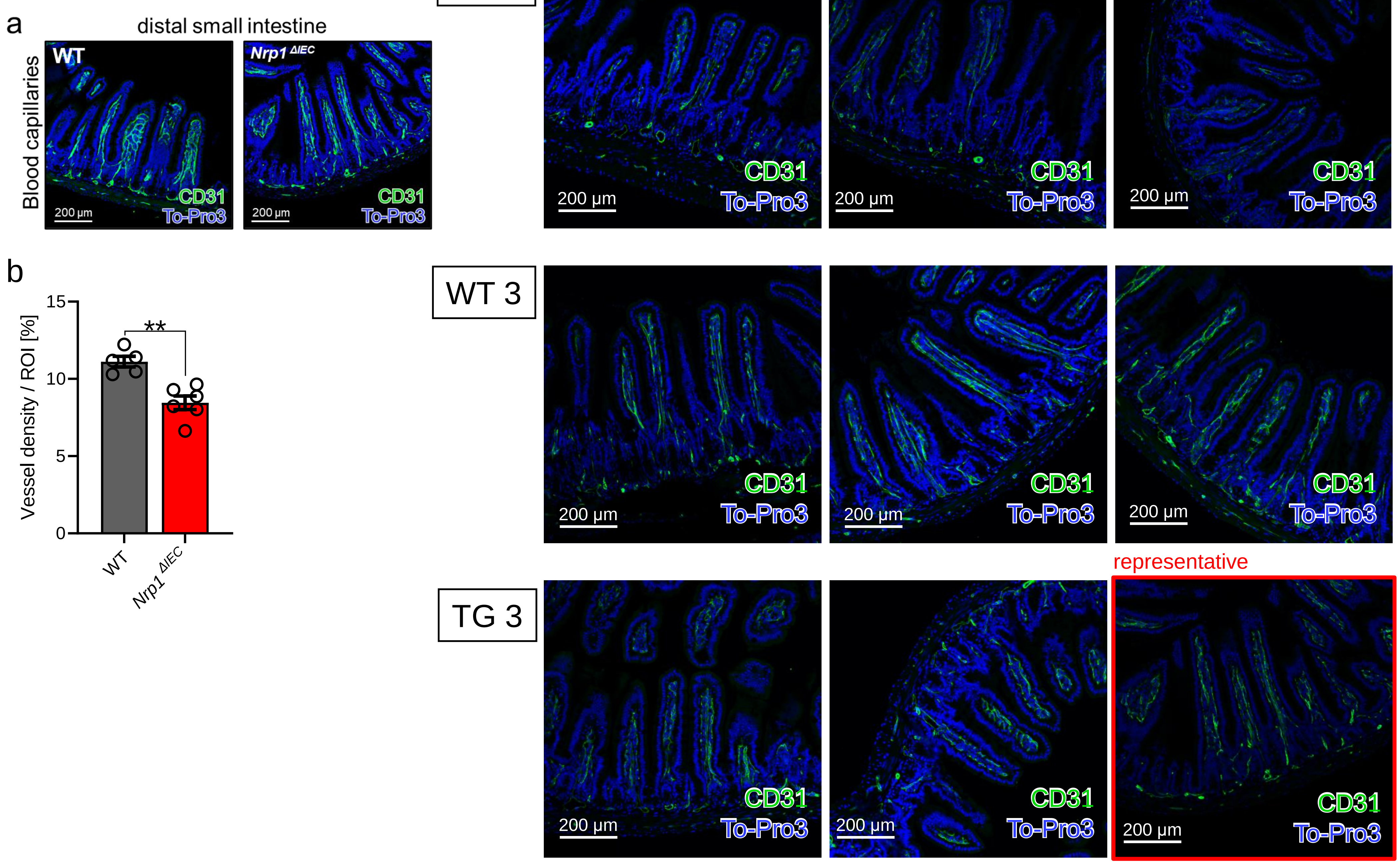
a
distal small intestine
Blood capillaries
WT
Nrp1 ΔIEC
CD31
To-Pro3
200 µm
b
Vessel density / ROI [%]
15
10
5
0
**
WT
Nrp1 ΔIEC
TG 2
WT 3
TG 3
representative
CD31
To-Pro3
200 µm

Figure 4a - b

a

distal small intestine

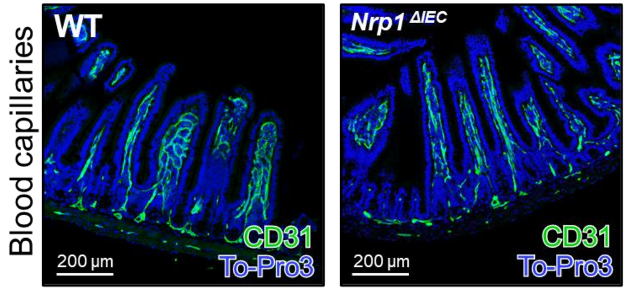

b

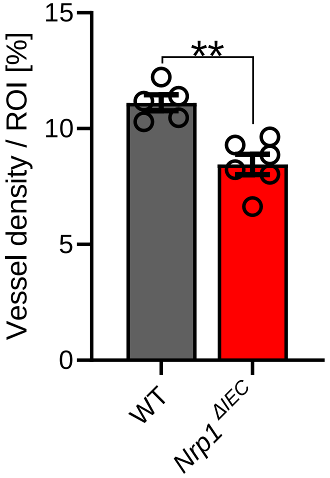

TG 4

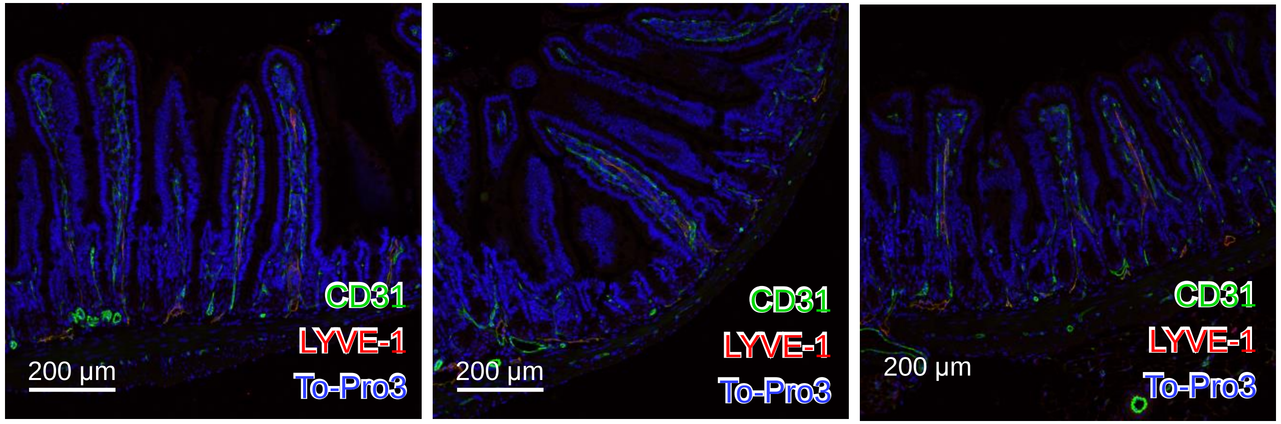

~~WT 4~~

outlier

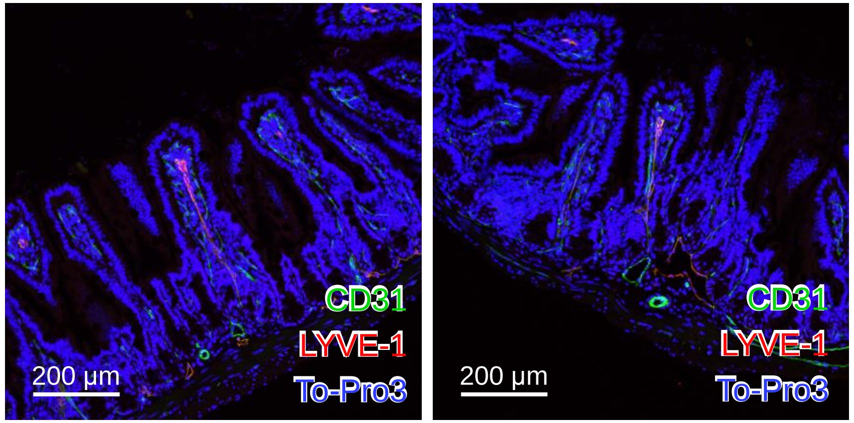

TG 5

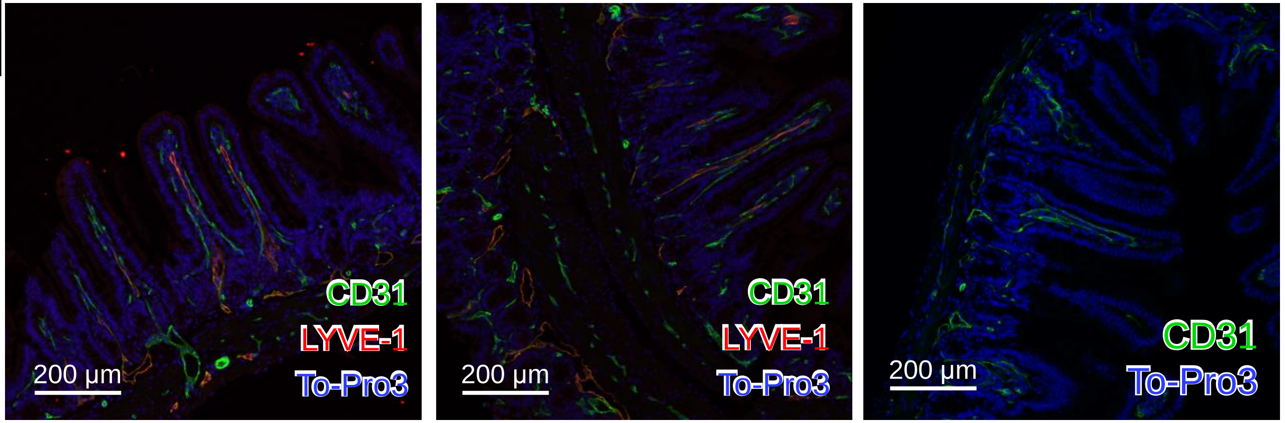

Figure 4a - b

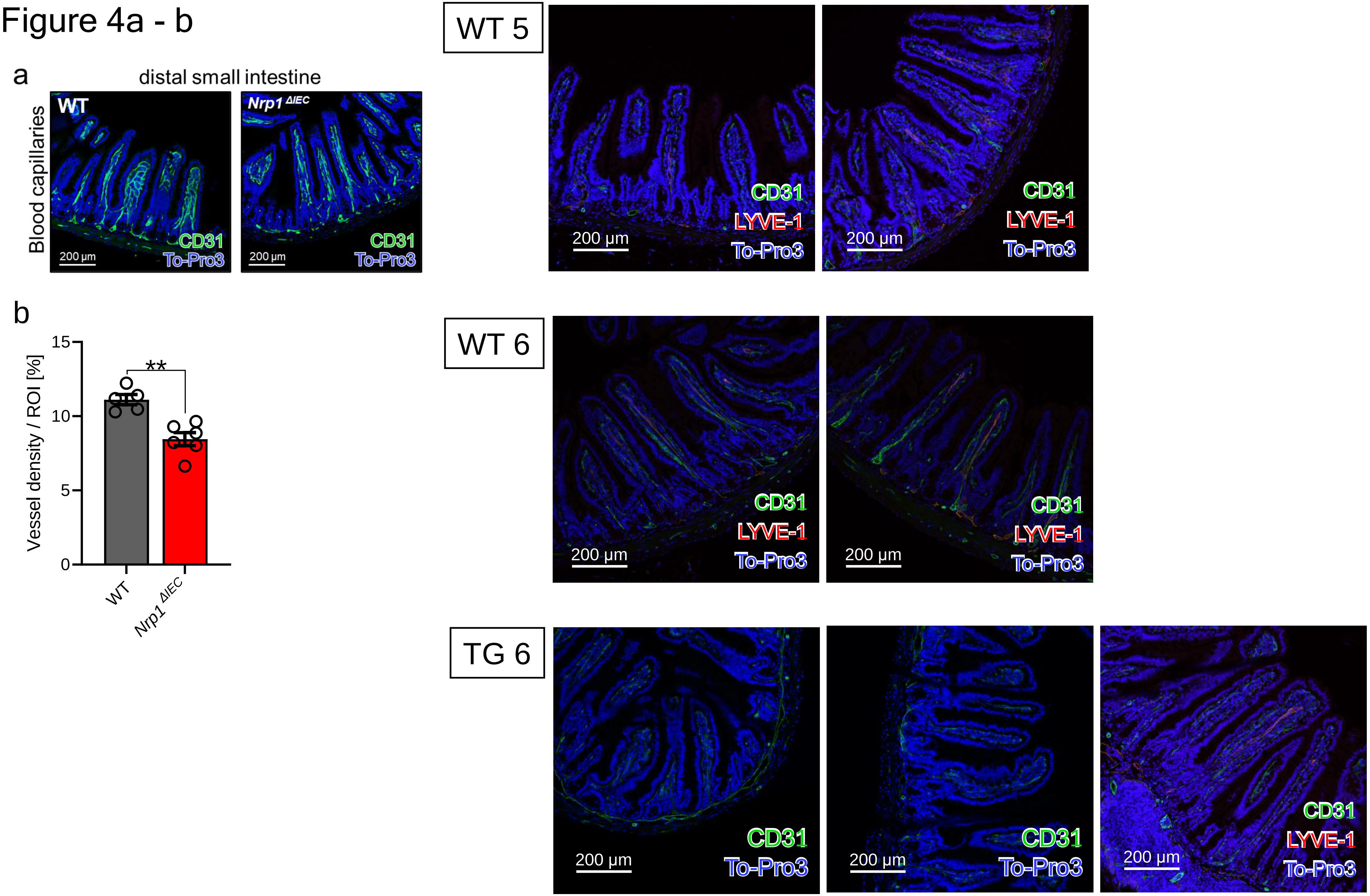

a
distal small intestine
Blood capillaries
WT
Nrp1 ΔIEC
CD31
To-Pro3
200 µm
b
Vessel density / ROI [%]
15
10
5
0
**
WT
Nrp1 ΔIEC
WT 5
WT 6
TG 6
CD31
LYVE-1
To-Pro3
200 µm

Figure 4d - f

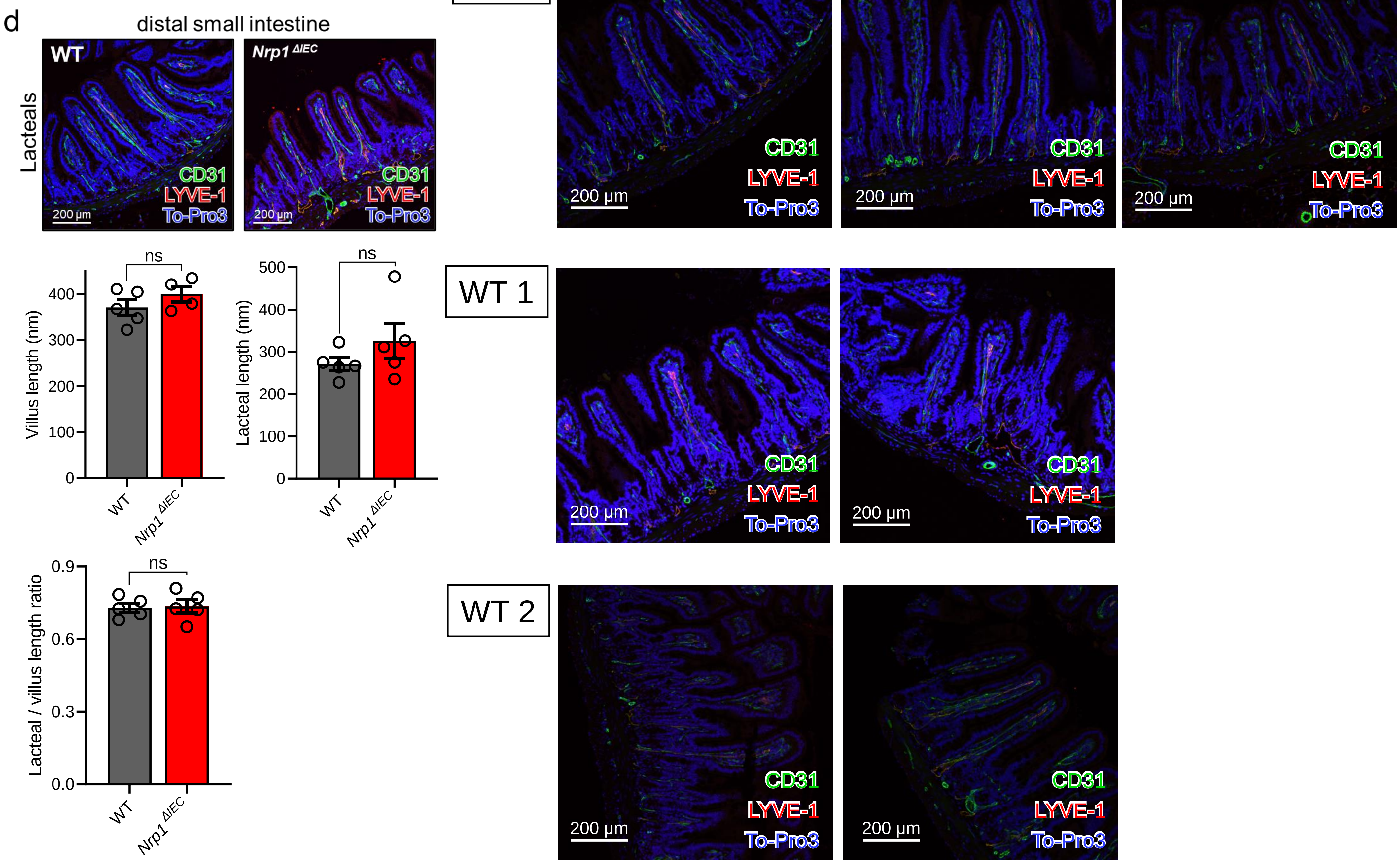
d
distal small intestine
WT
Nrp1 ΔIEC
Lacteals
CD31
LYVE-1
To-Pro3
200 µm
ns
Villus length (nm)
Lacteal length (nm)
Lacteal / villus length ratio
WT
Nrp1 ΔIEC
TG 1
WT 1
WT 2

Figure 4d - f

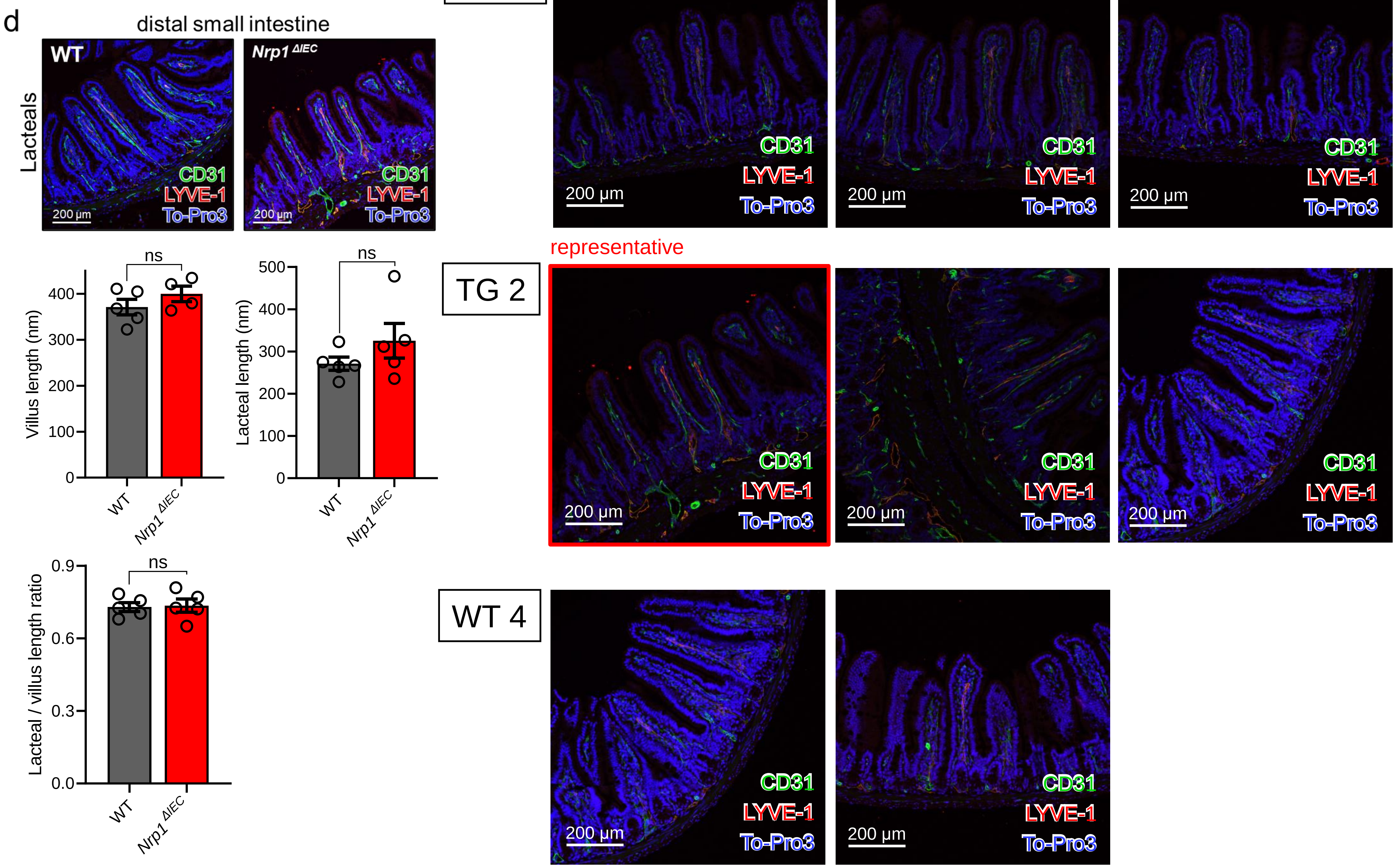
d
distal small intestine
WT
Nrp1 ΔIEC
Lacteals
CD31
LYVE-1
To-Pro3
200 µm
ns
Villus length (nm)
Lacteal length (nm)
Lacteal / villus length ratio
WT
Nrp1 ΔIEC
WT 3
TG 2
WT 4
representative

Figure 4d - f

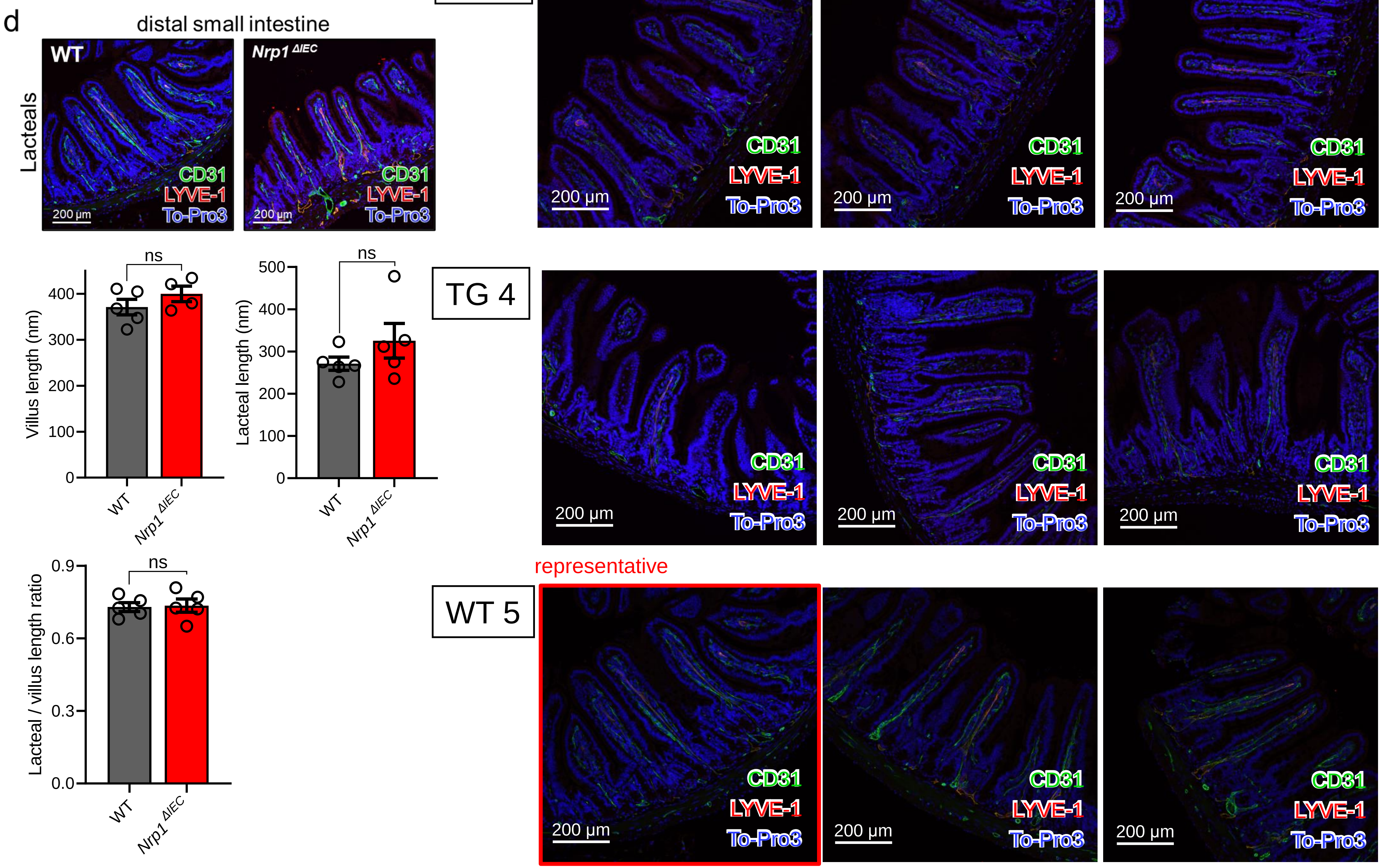
d
distal small intestine
WT
Nrp1 ΔIEC
Lacteals
CD31
LYVE-1
To-Pro3
200 µm
ns
Villus length (nm)
0
100
200
300
400
Lacteal length (nm)
500
WT
Nrp1 ΔIEC
Lacteal / villus length ratio
0.0
0.3
0.6
0.9
TG 3
TG 4
WT 5
representative

Figure 4d - f

d

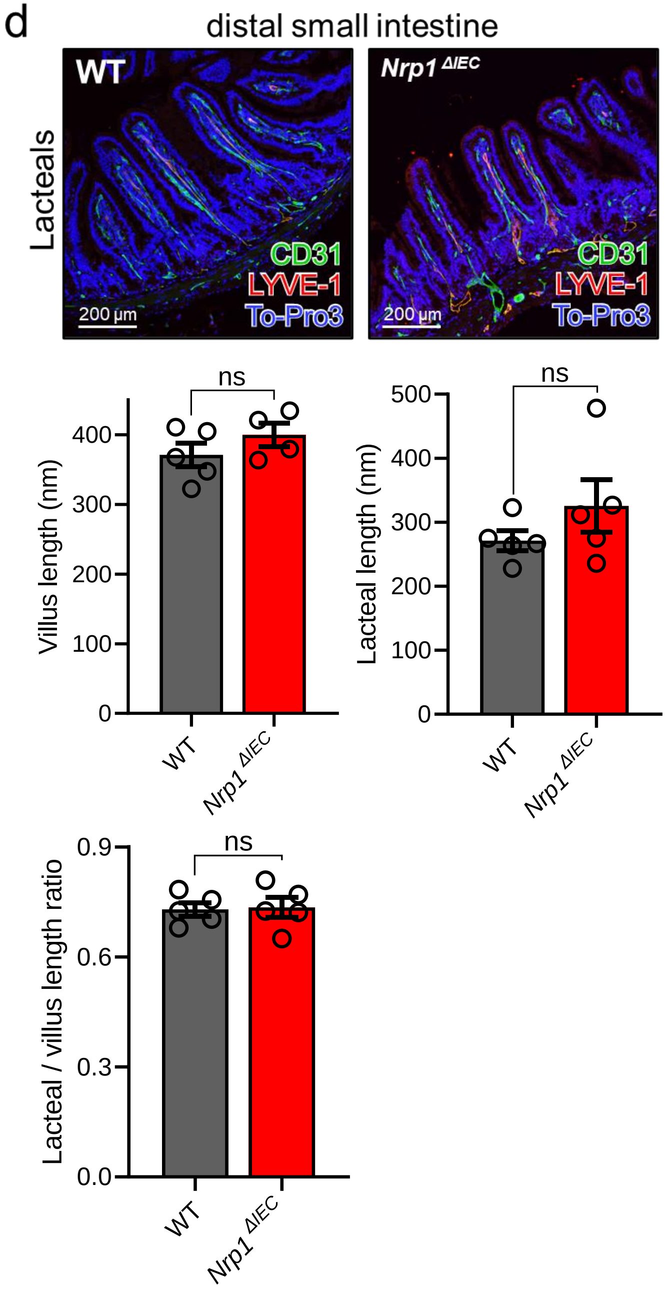

TG 5

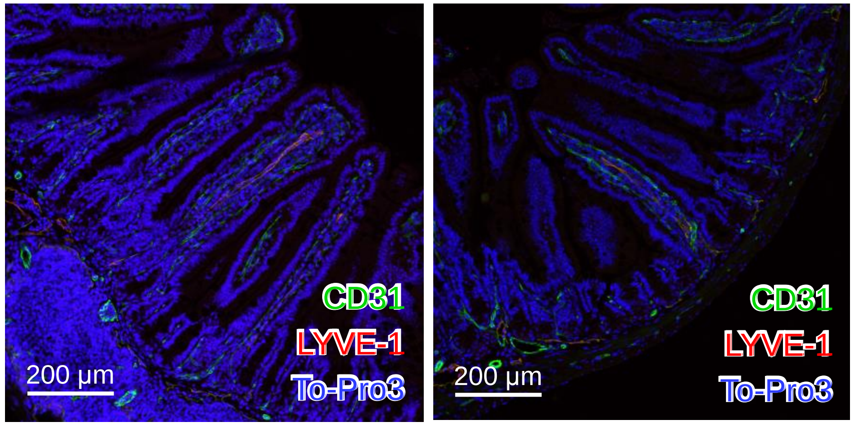

TG5 was excluded from the villus length panel as outlier
