# Supplementary material for: Commensal bacteria weaken the intestinal barrier by suppressing epithelial neuropilin-1 and Hedgehog signaling
Source: Nat Metab. 2023 Jul 6;5(7):1174–87. doi: 10.1038/s42255-023-00828-5 (PMC10365997; doi:10.1038/s42255-023-00828-5)

Supplementary Figure 3c

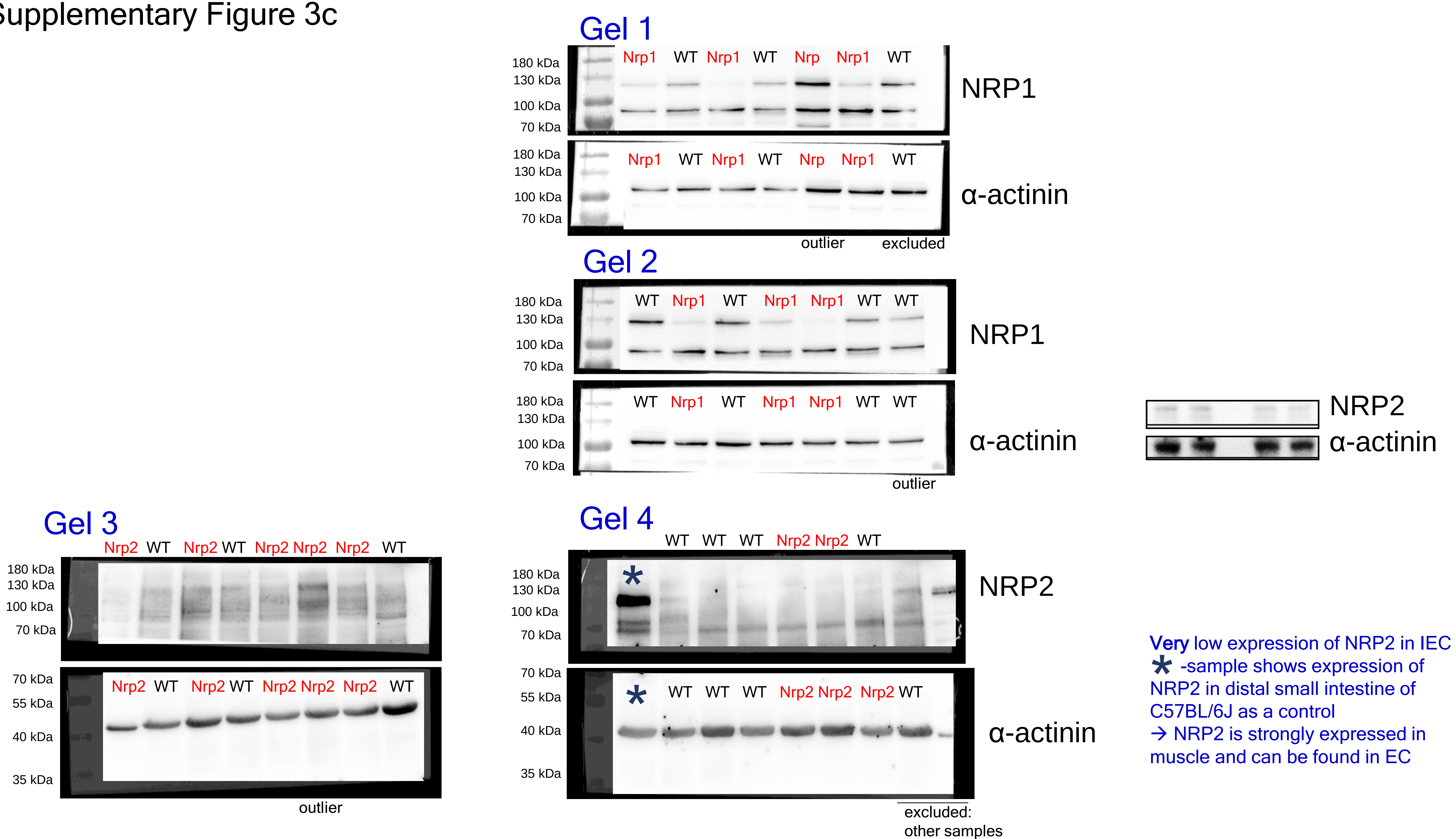

Supplementary Figure 3i

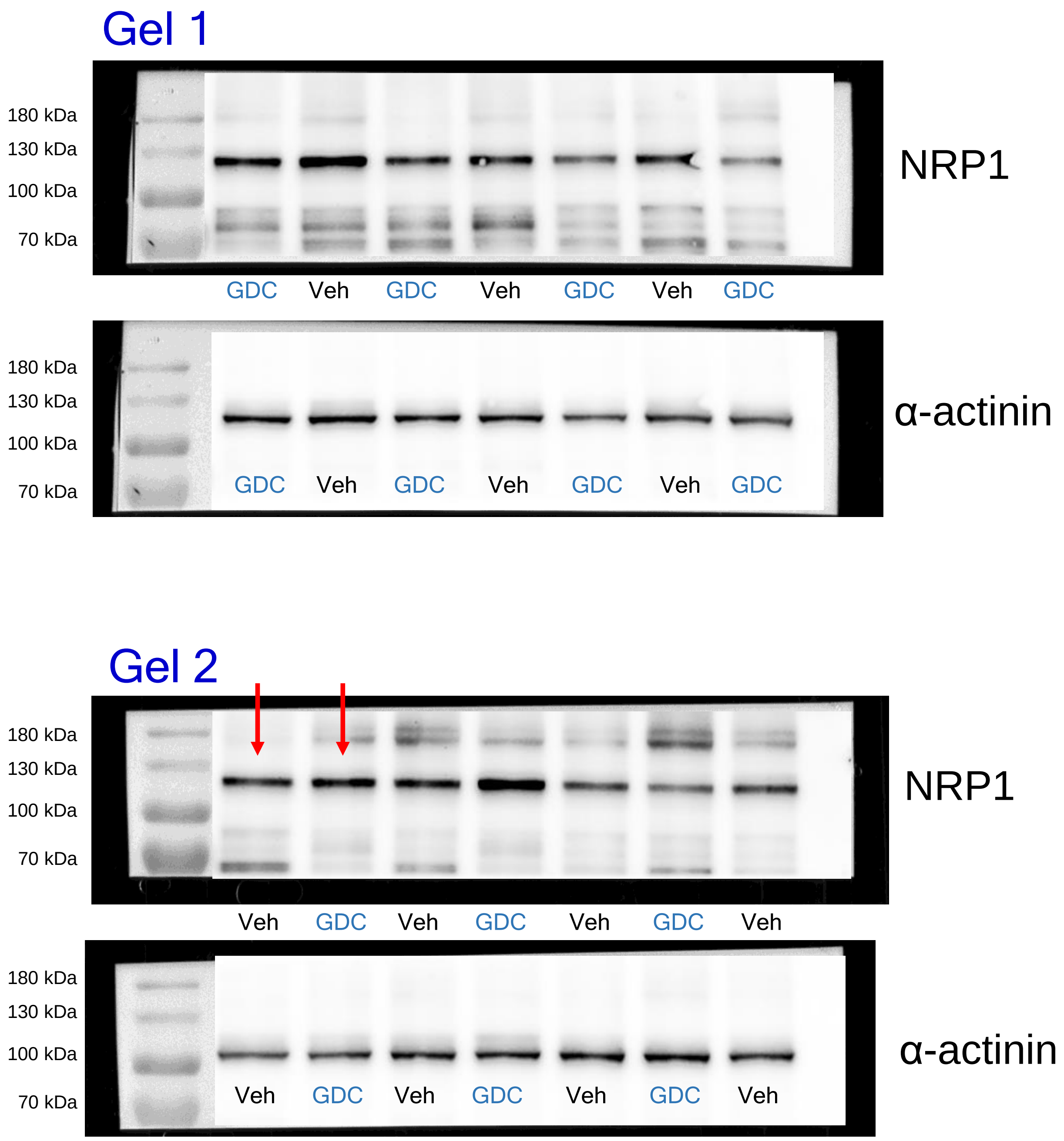

Supplementary Figure 3j

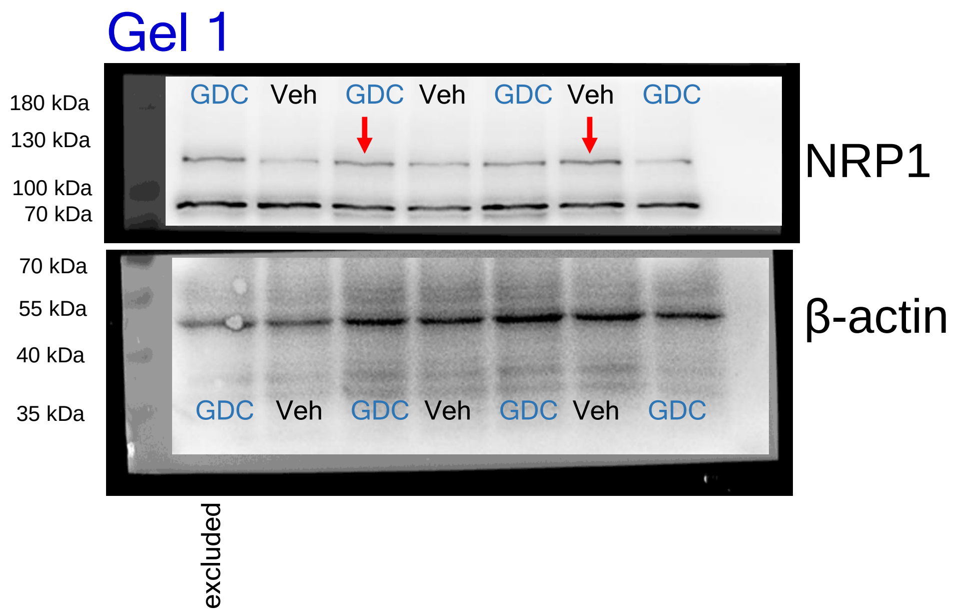

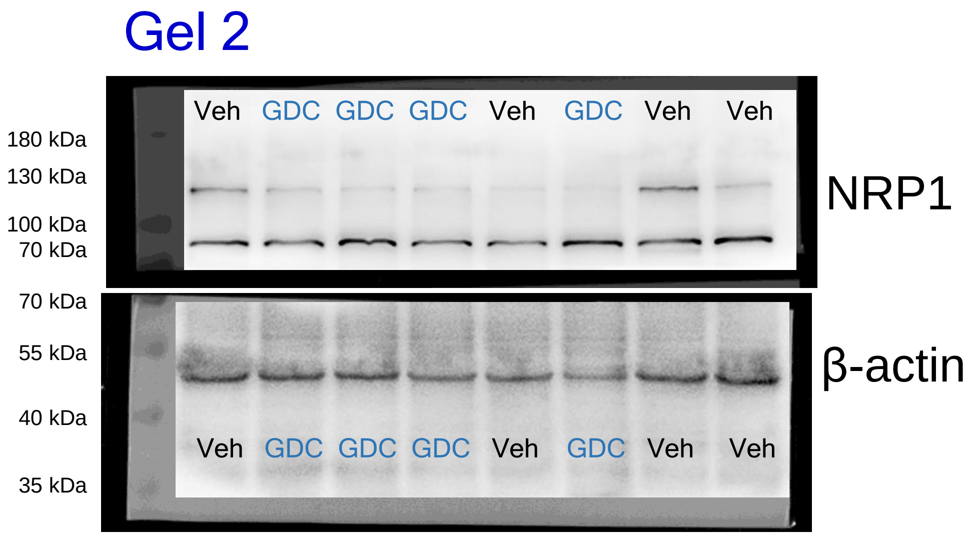

Supplement: Source Data Extended Data Fig. 3 — Unprocessed western blots for Extended Data Fig. 3. [file 42255_2023_828_MOESM19_ESM.pdf]
