# Supplementary material for: Commensal bacteria weaken the intestinal barrier by suppressing epithelial neuropilin-1 and Hedgehog signaling
Source: Nat Metab. 2023 Jul 6;5(7):1174–87. doi: 10.1038/s42255-023-00828-5 (PMC10365997; doi:10.1038/s42255-023-00828-5)

Extended Data Figure 4b

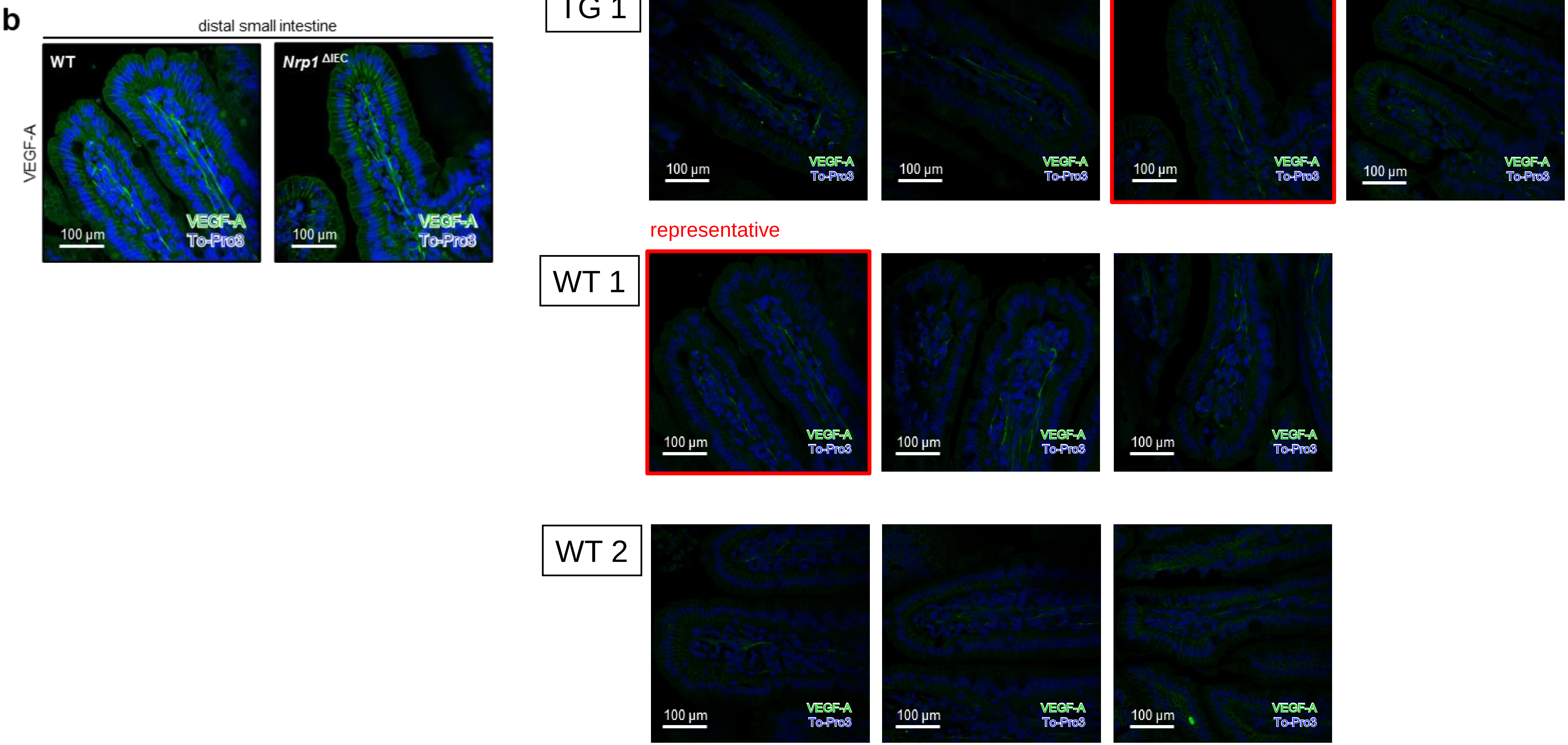

Extended Data Figure 4b

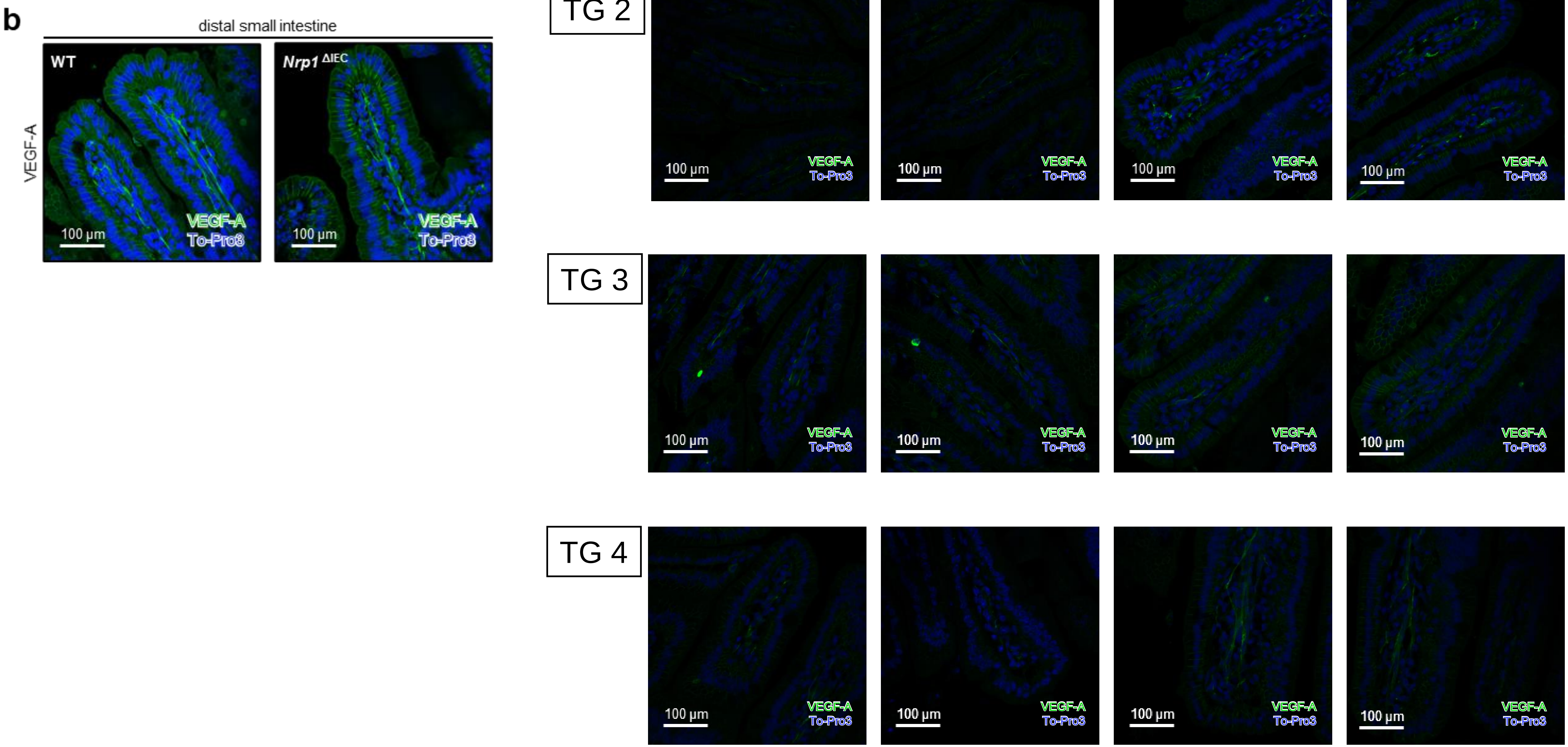

Extended Data Figure 4b

b

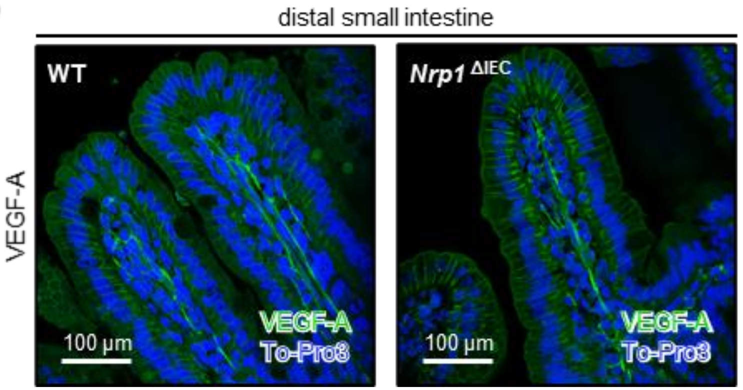

WT 3

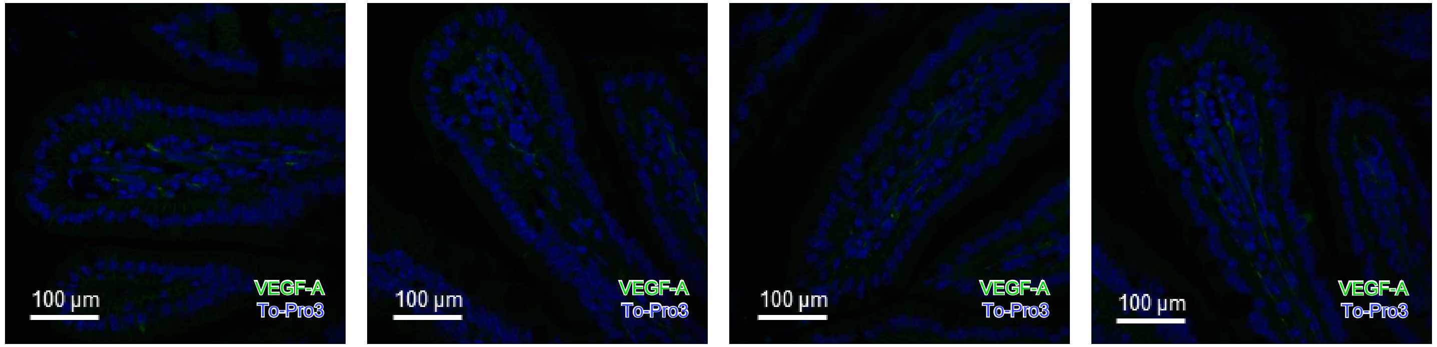

WT 4

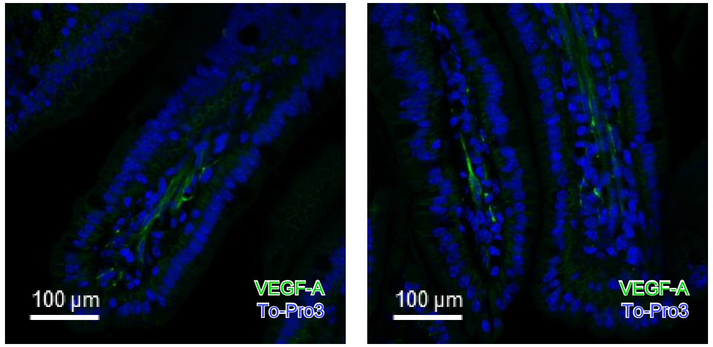

Extended Data Figure 4c

c

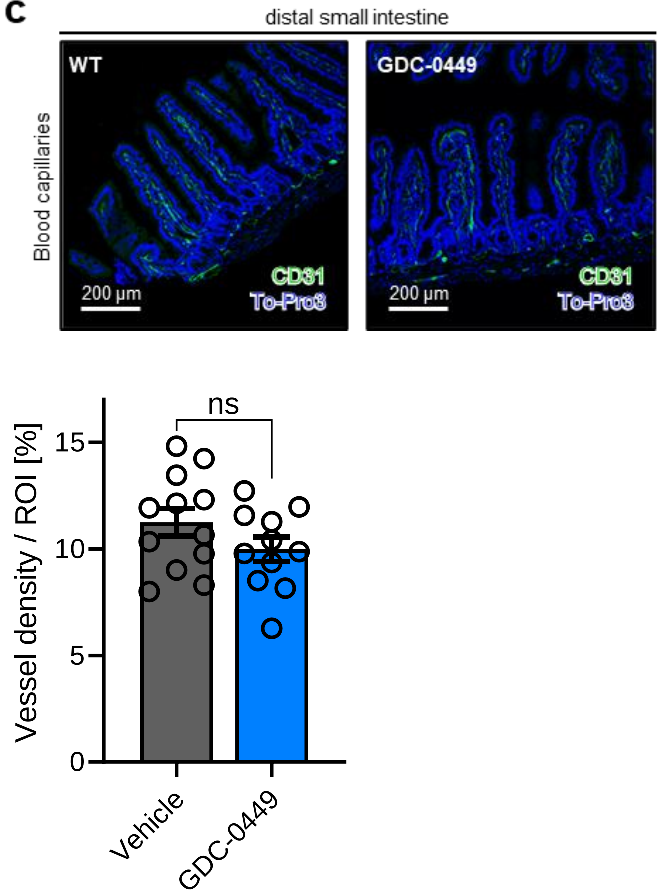

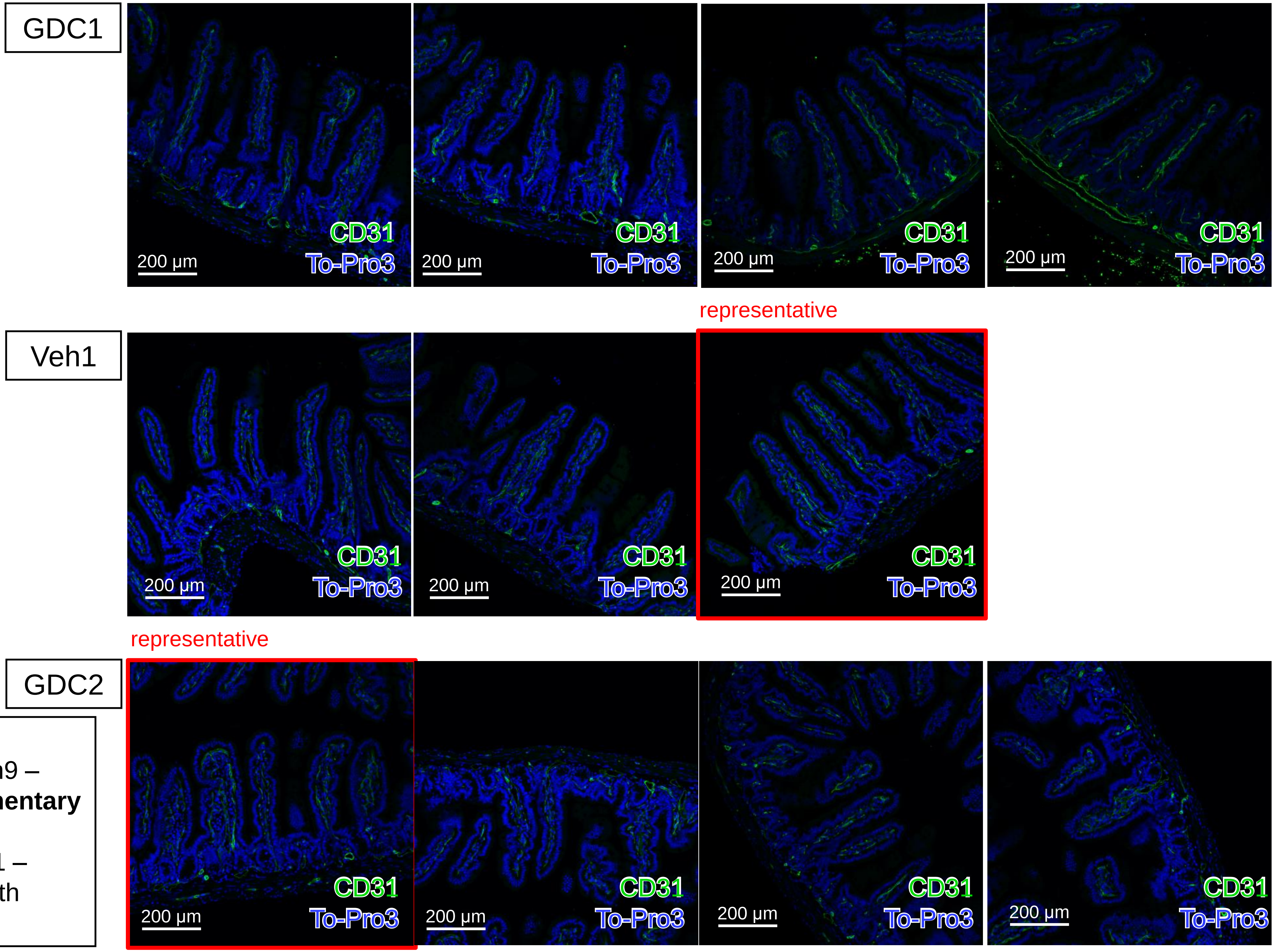

<u>Note</u>

1. Images of GDC7 – GDC11 and Veh9 – Veh13 were also used for **Supplementary Figure 4f – i**
2. Images of GDC1 – GDC5 and Veh1 – Veh5 were also used for Villus length analysis in **Supplementary 4g**

Extended Data Figure 4c

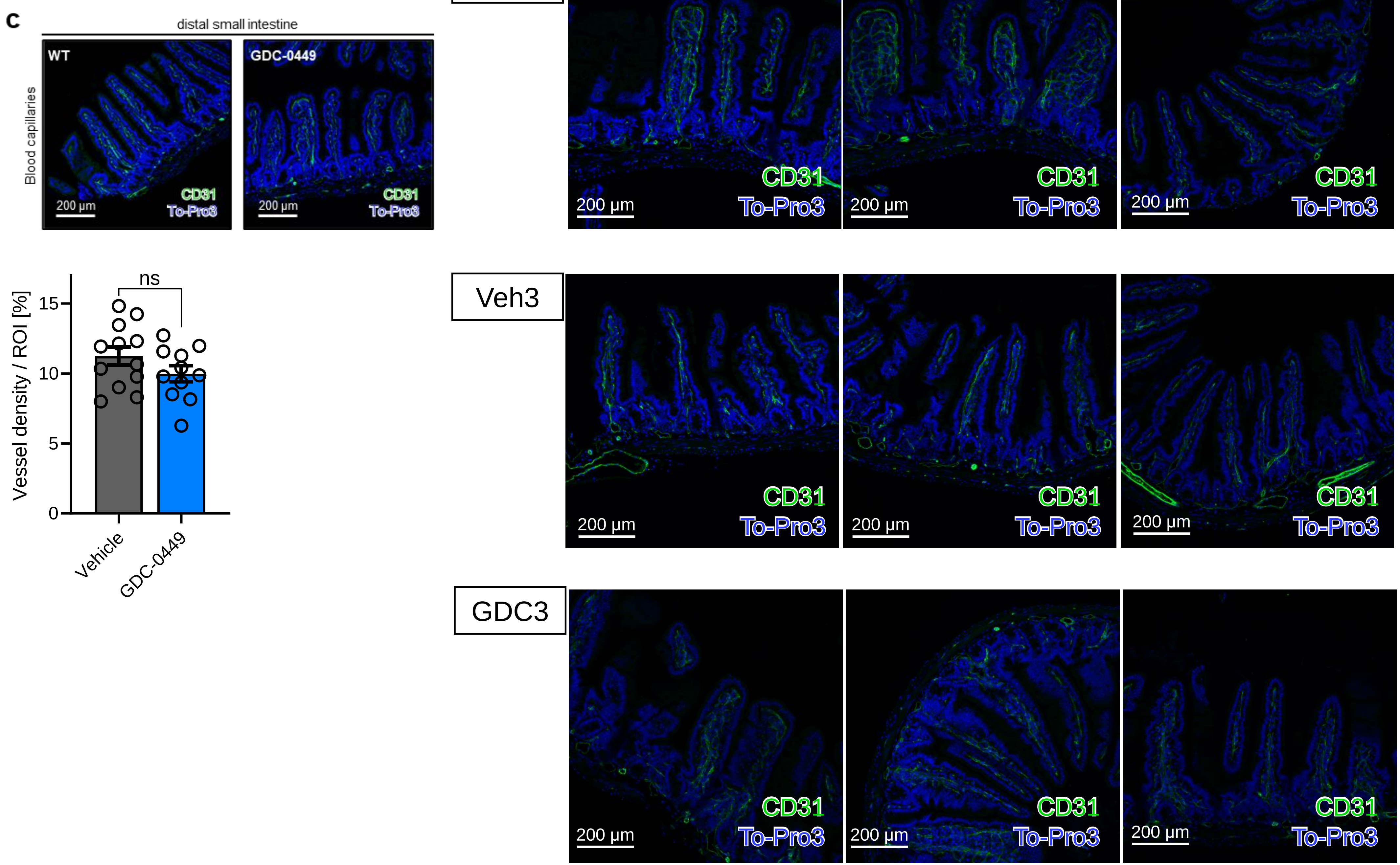

Extended Data Figure 4c

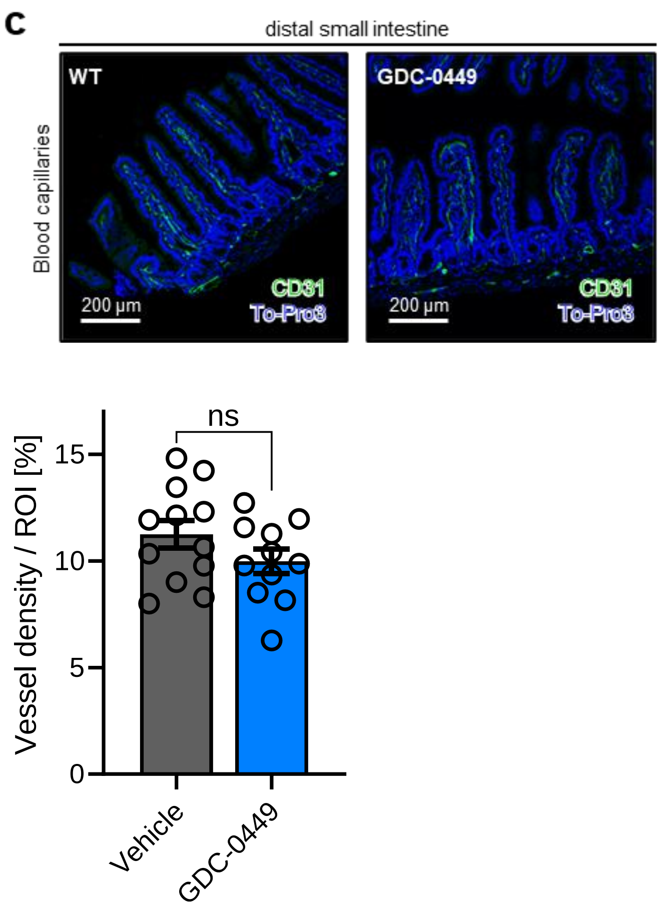

GDC4

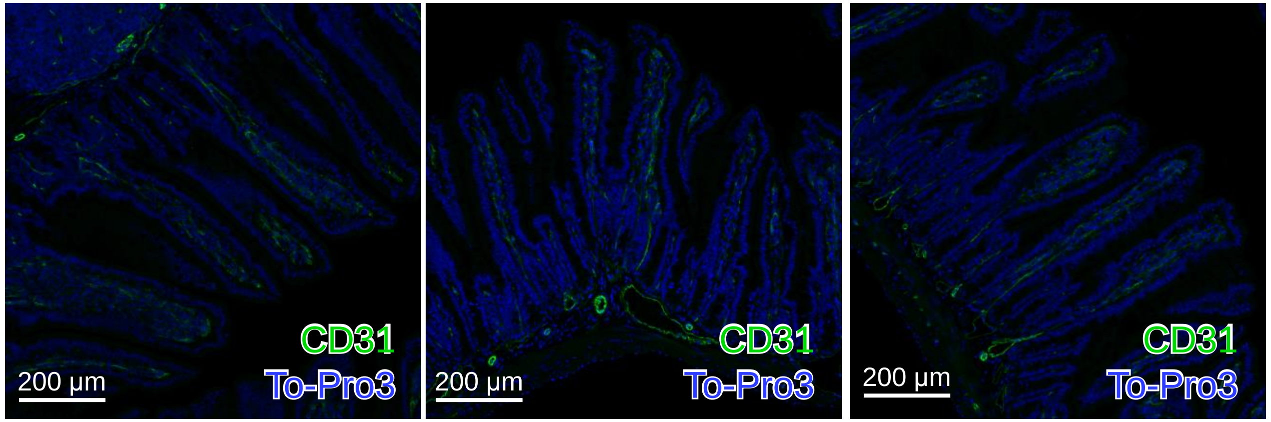

Veh4

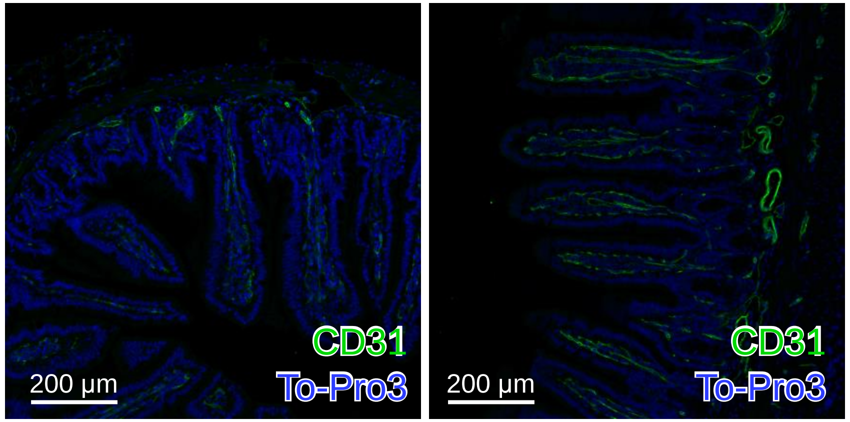

GDC5

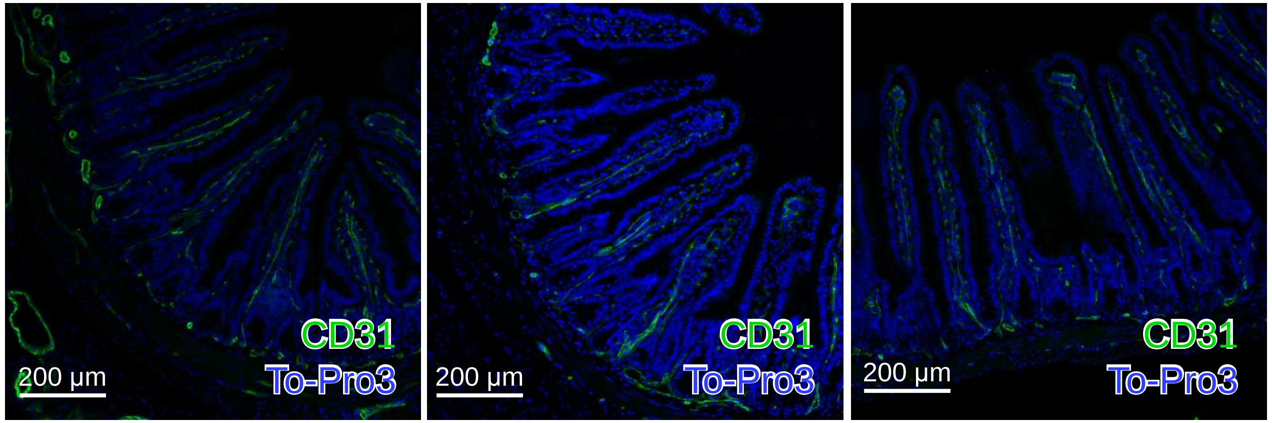

Extended Data Figure 4c

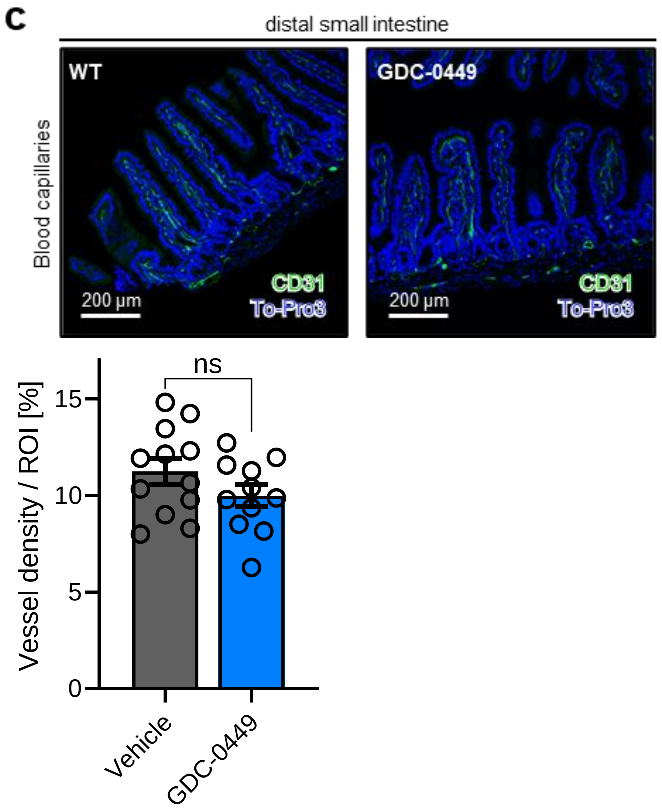

GDC6

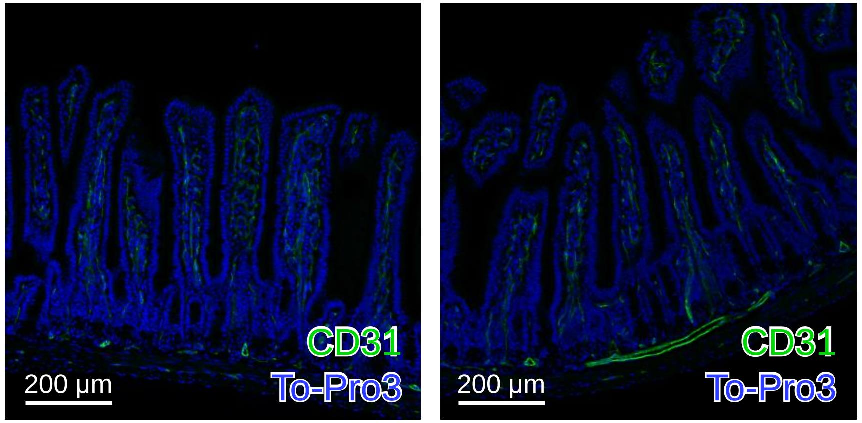

Veh5

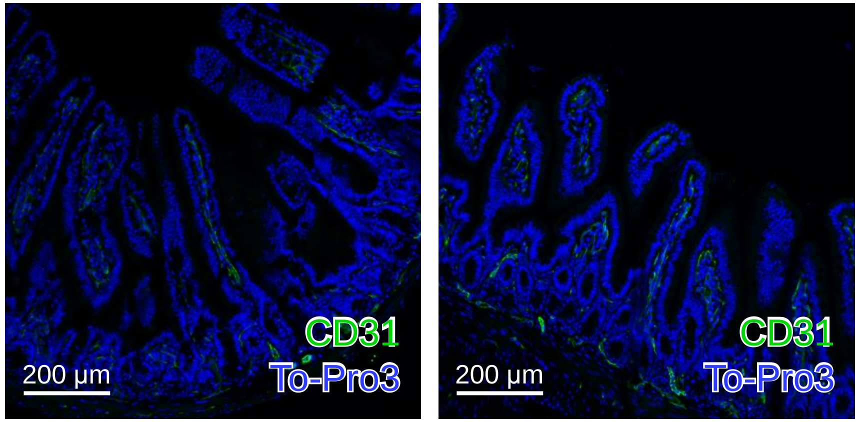

Extended Data Figure 4c

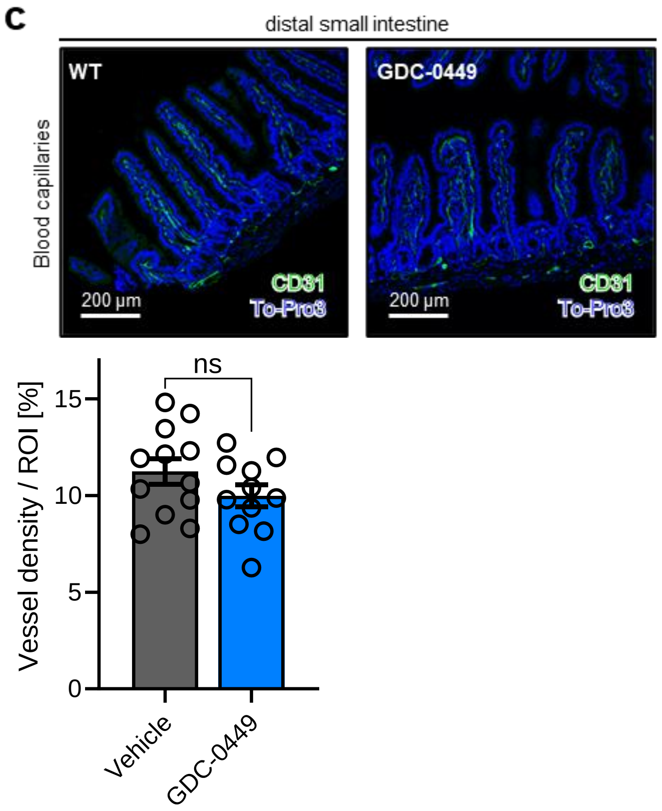

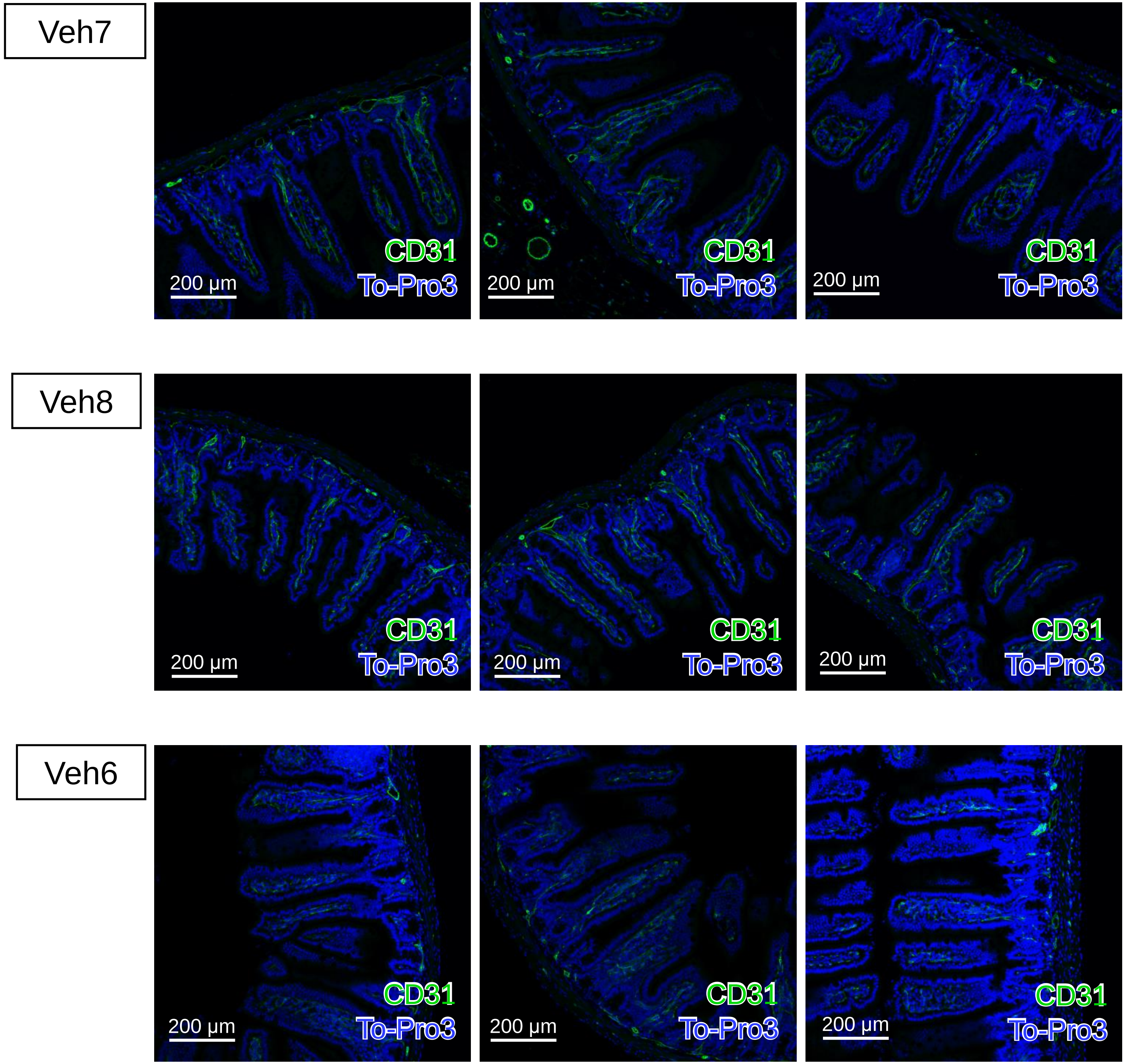

Extended Data Figure 4c

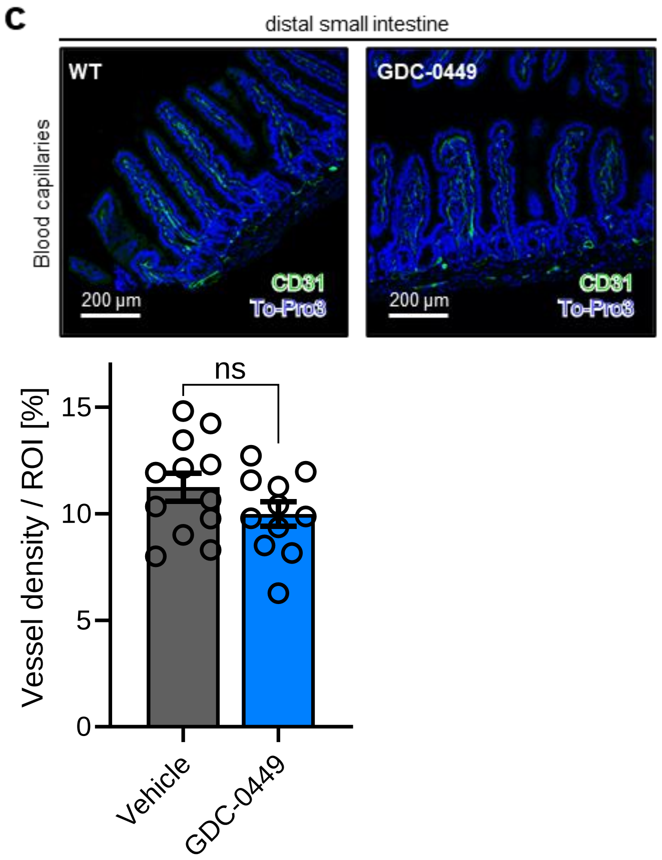

GDC7

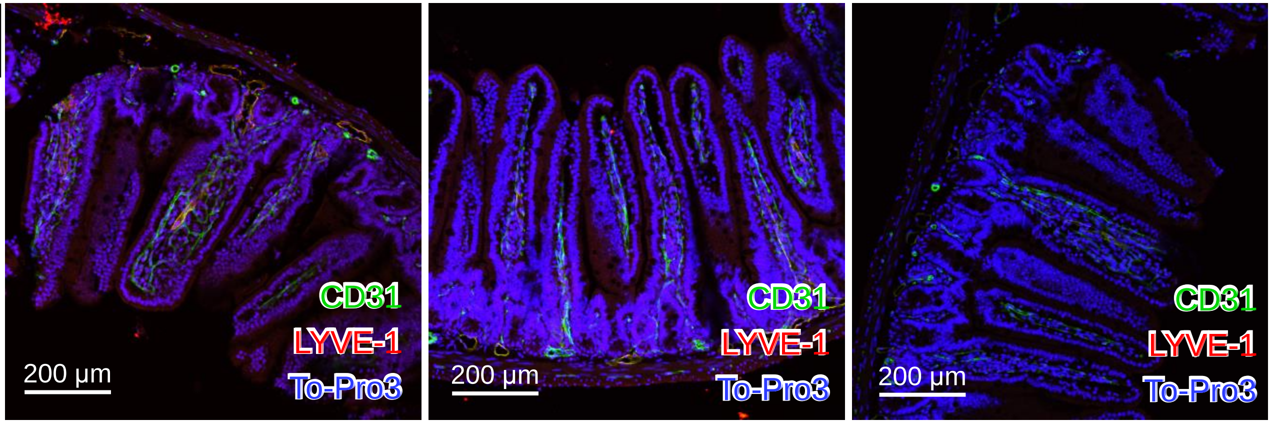

Veh9

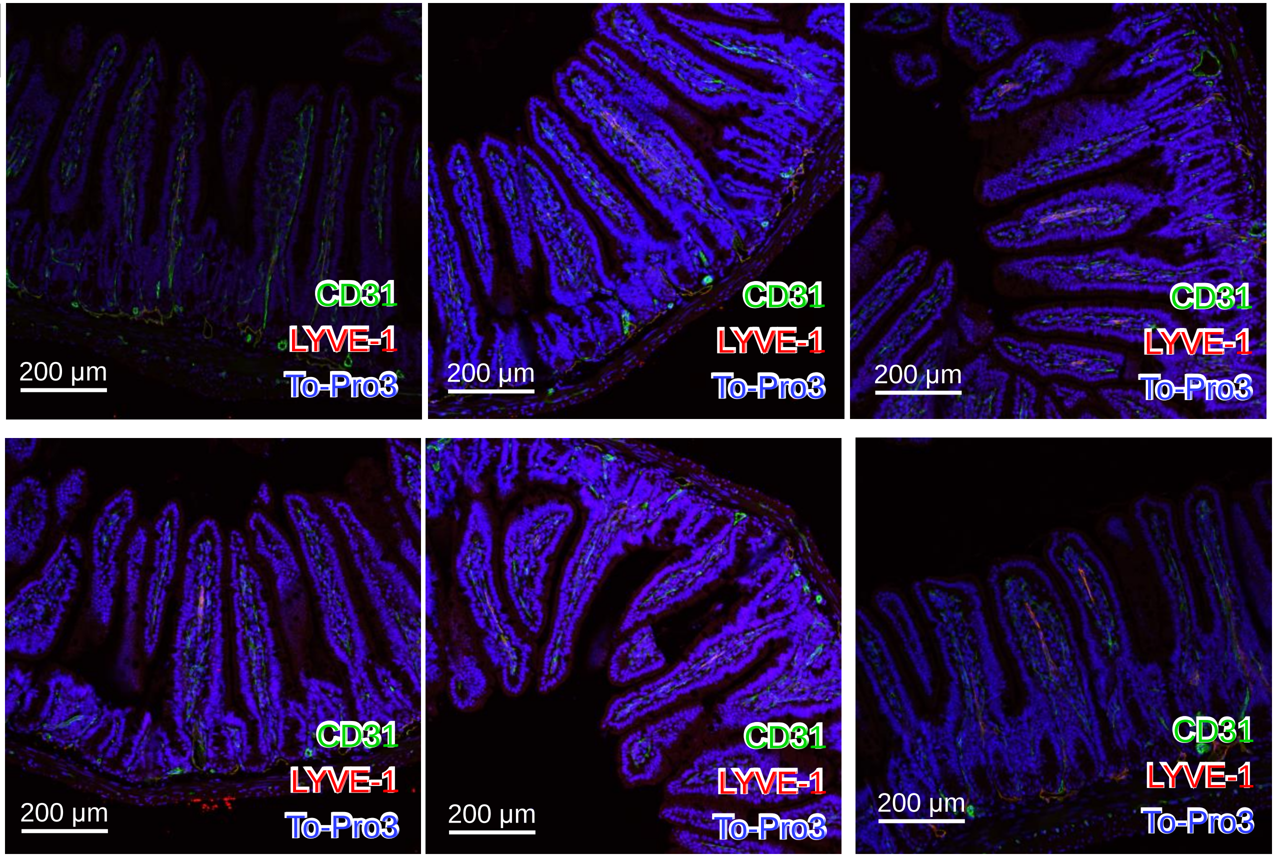

Extended Data Figure 4c

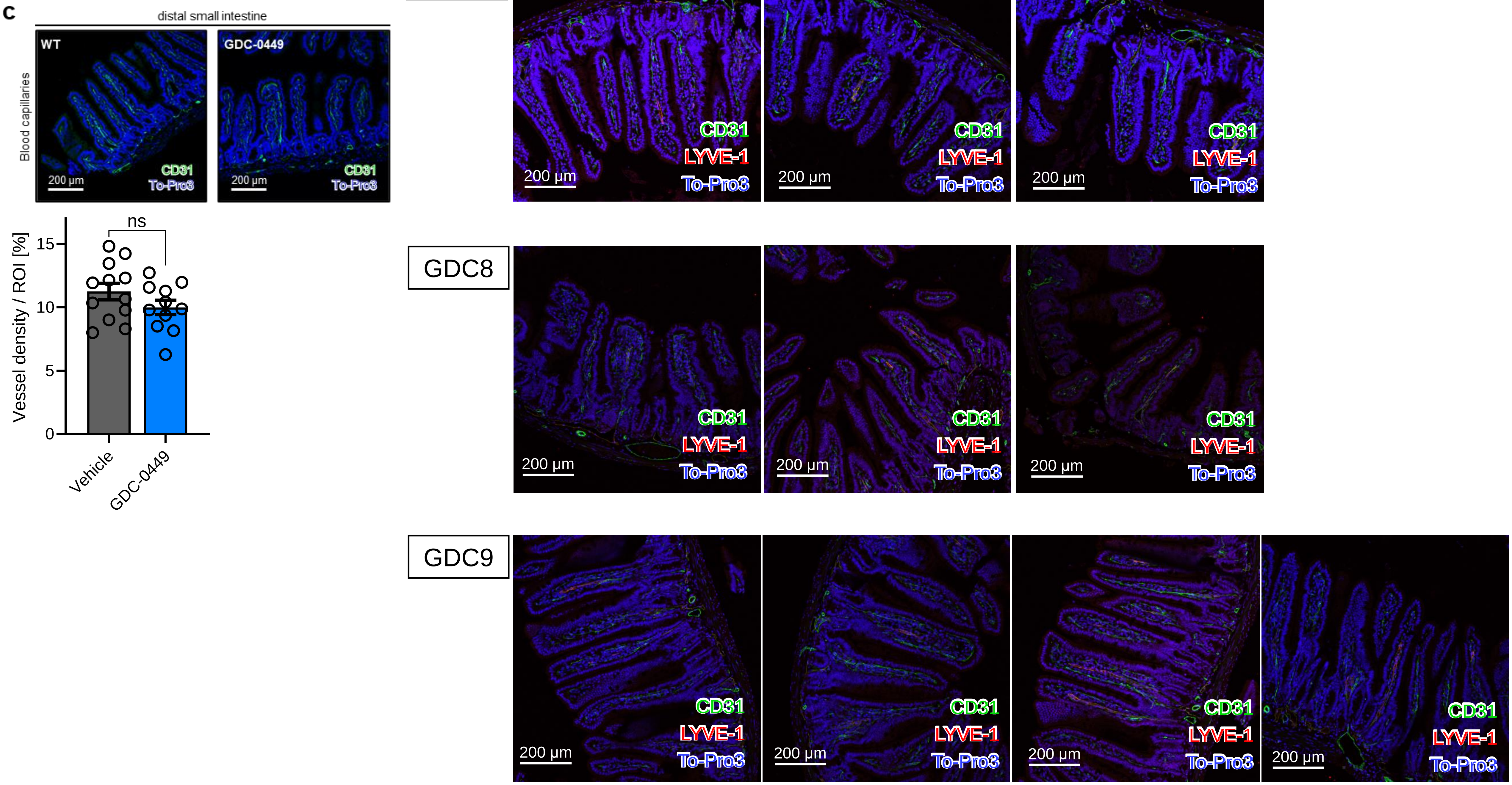

Extended Data Figure 4c

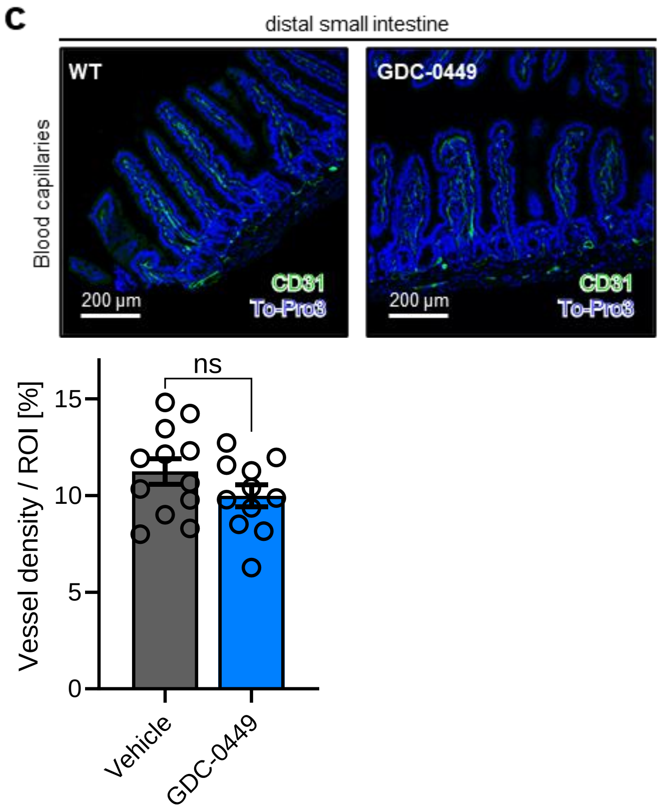

GDC10

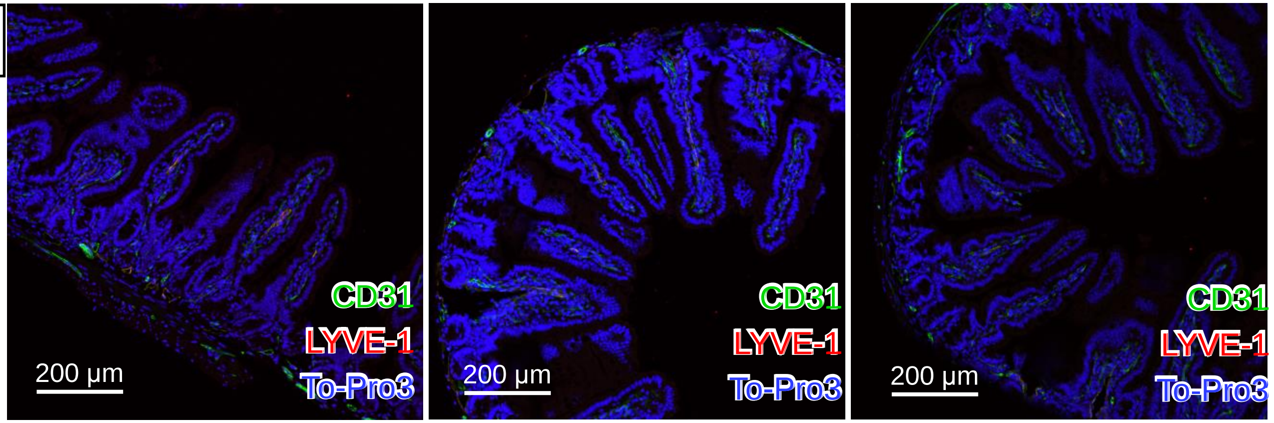

Veh11

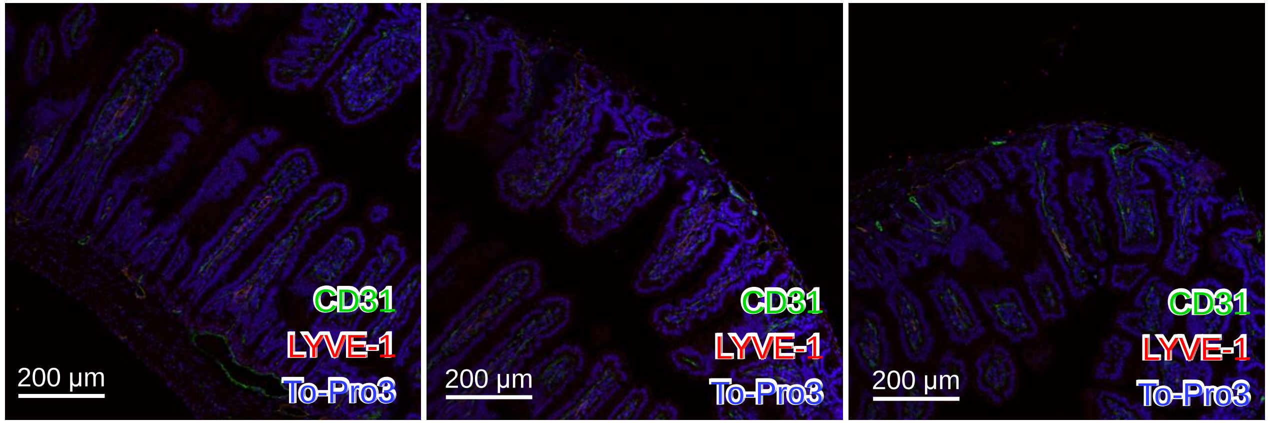

Excluded because of weak CD31 signal

Veh12

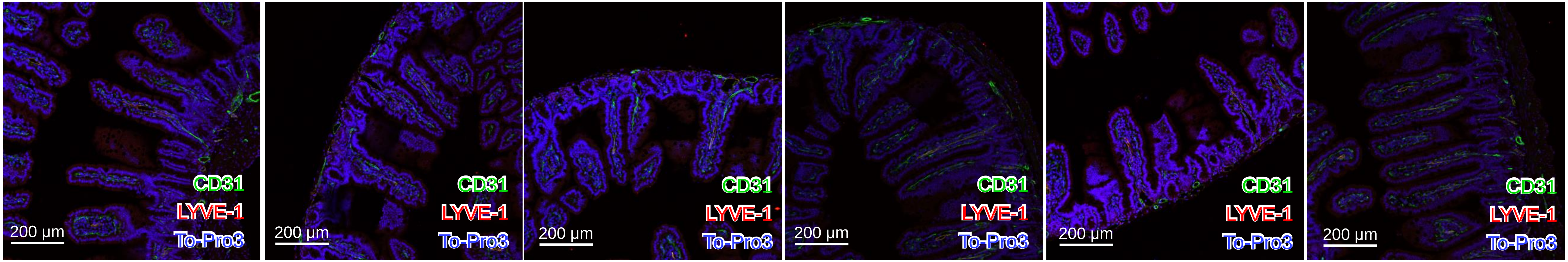

Extended Data Figure 4c

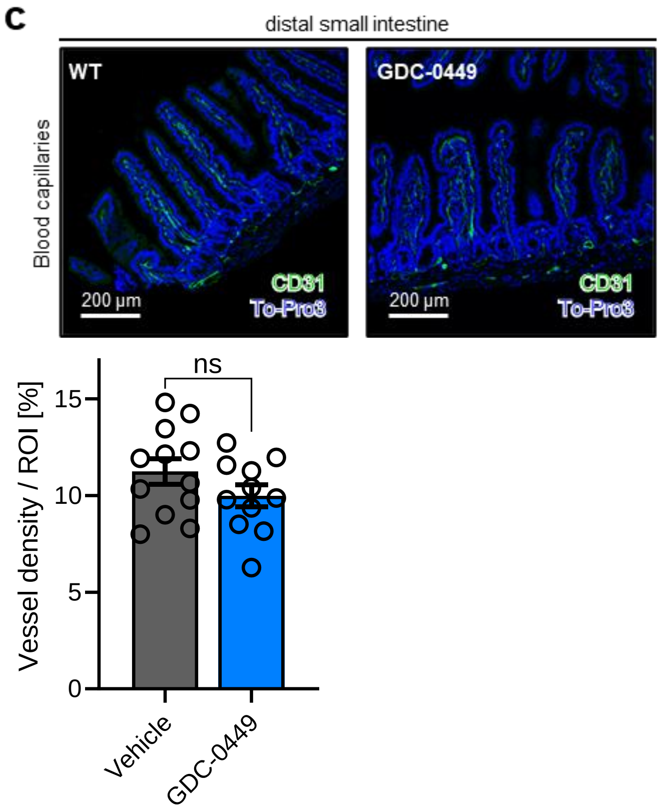

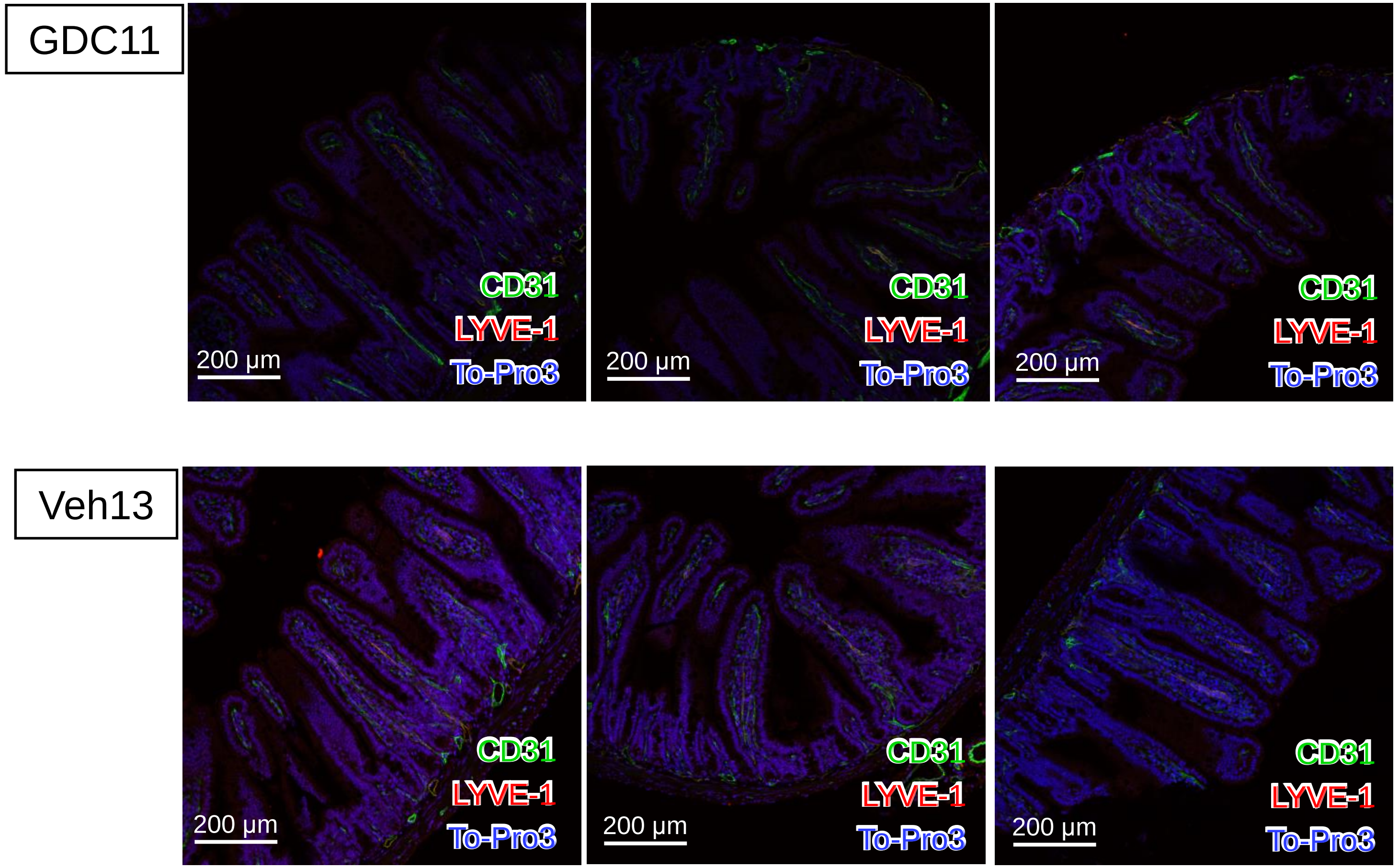

Supplementary Figure 4f - i

f

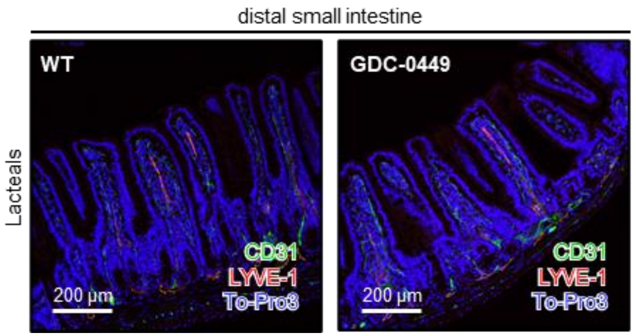

g

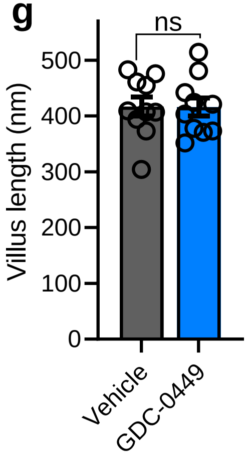

h

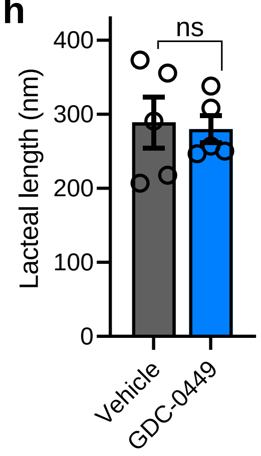

i

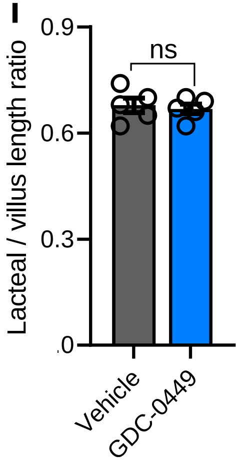

GDC7

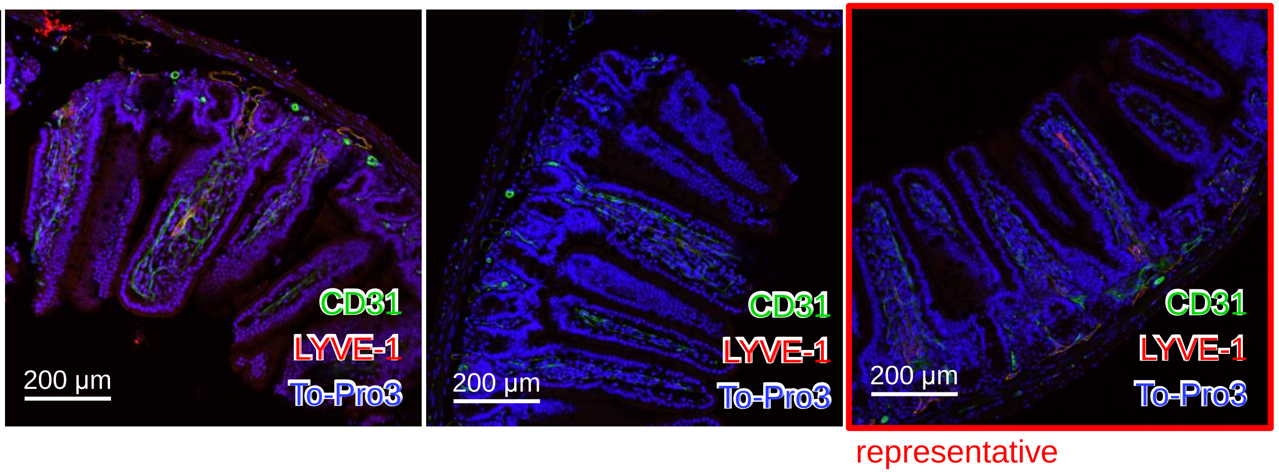

Veh9

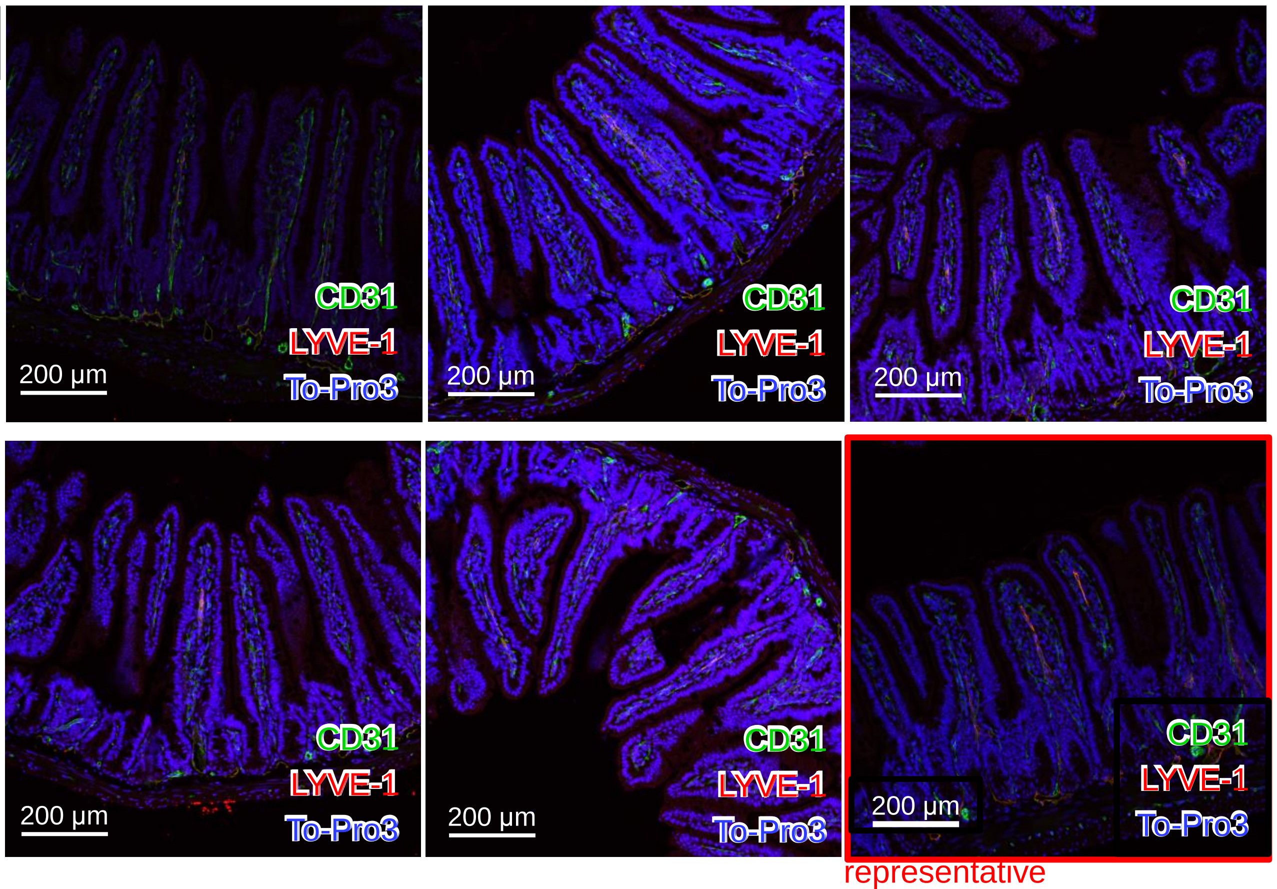

Note

1. Images of GDC1 – GDC5 and Veh1 – Veh5 from **Supplementary c – d** were also used for Villus length analysis in **Supplementary 4g**

Supplementary Figure 4f - i

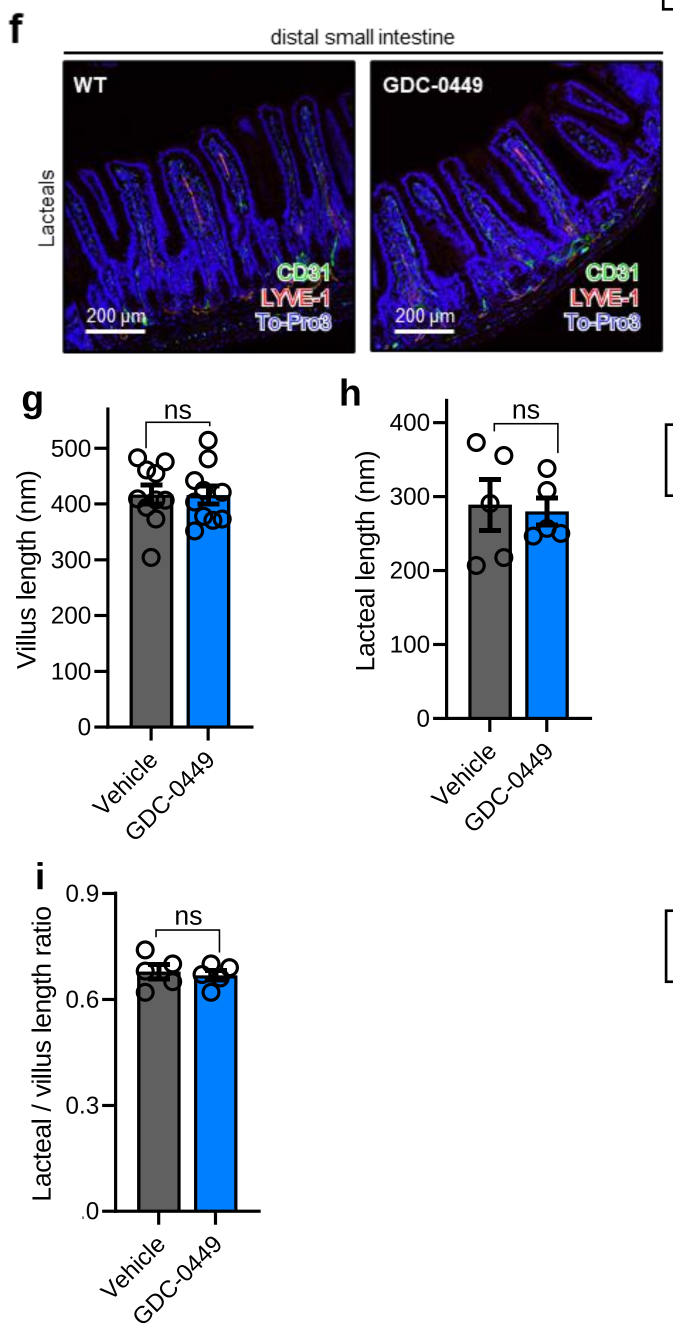

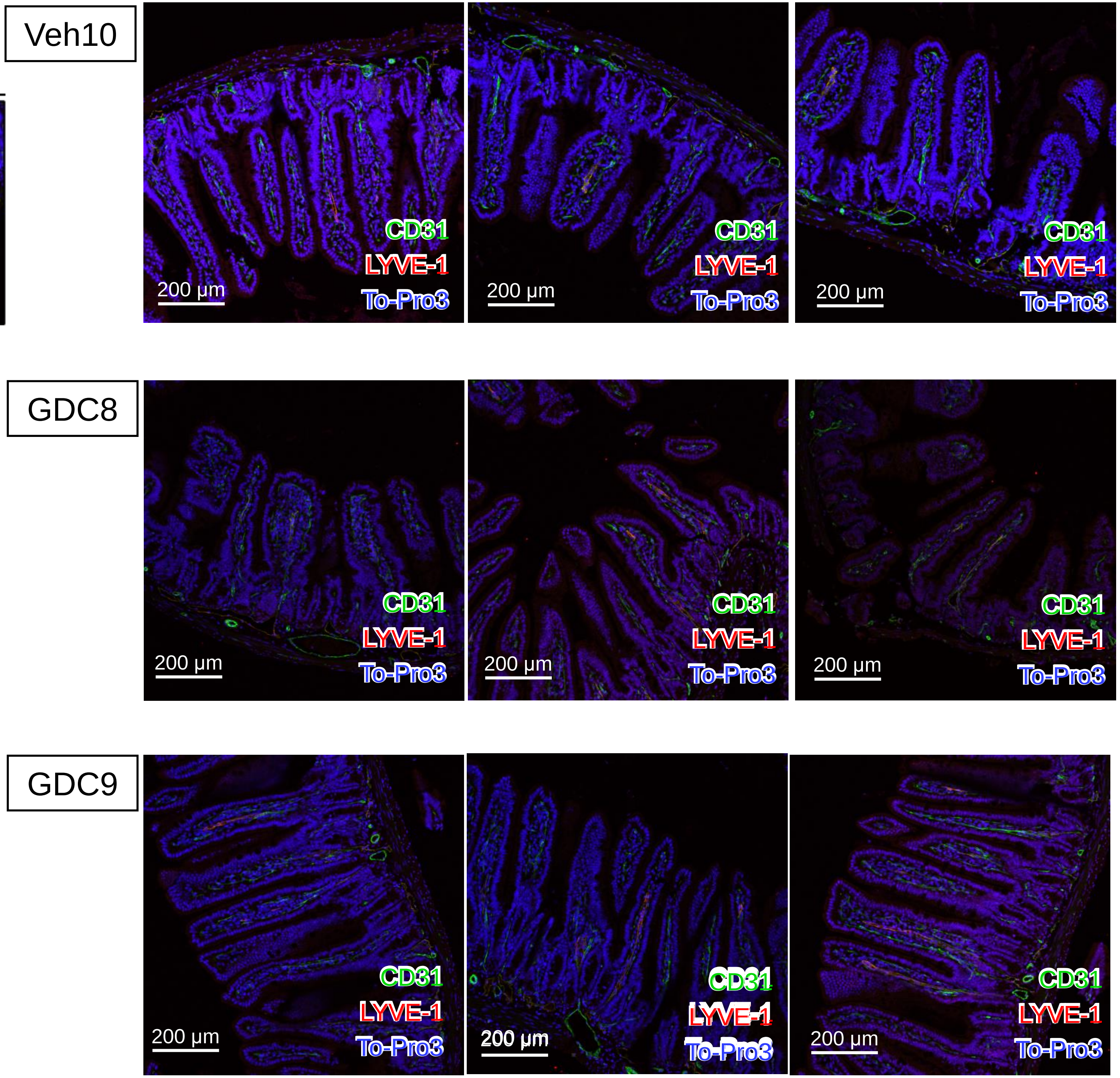

Supplementary Figure 4f - i

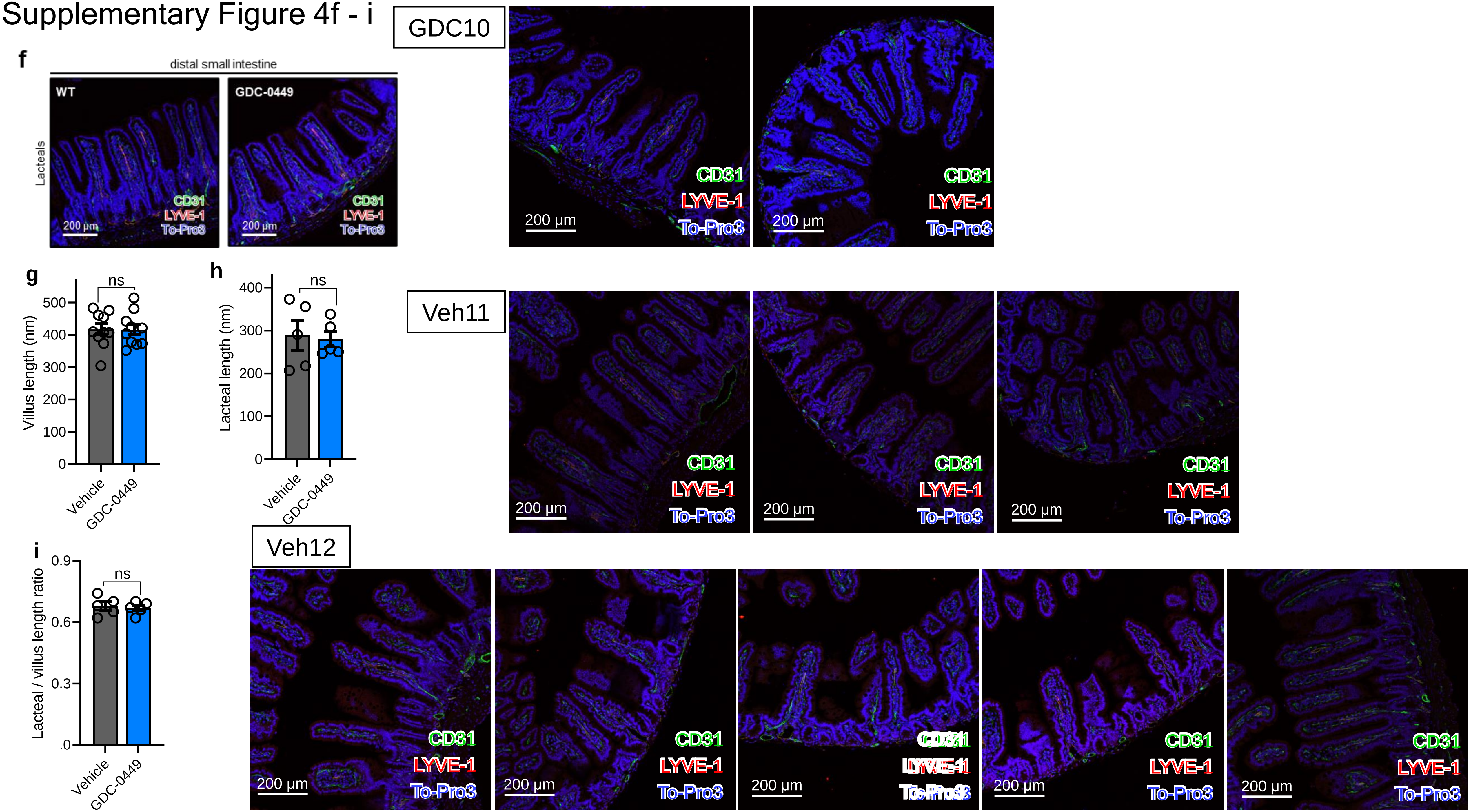

Supplementary Figure 4f - i

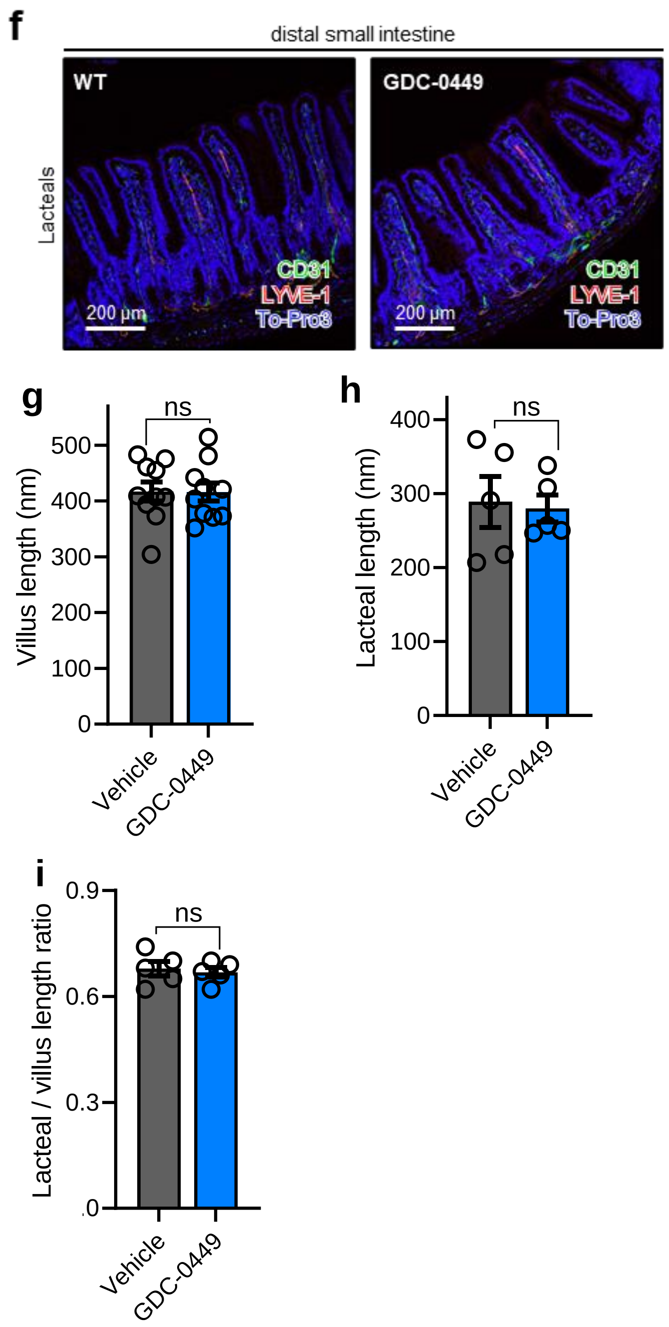

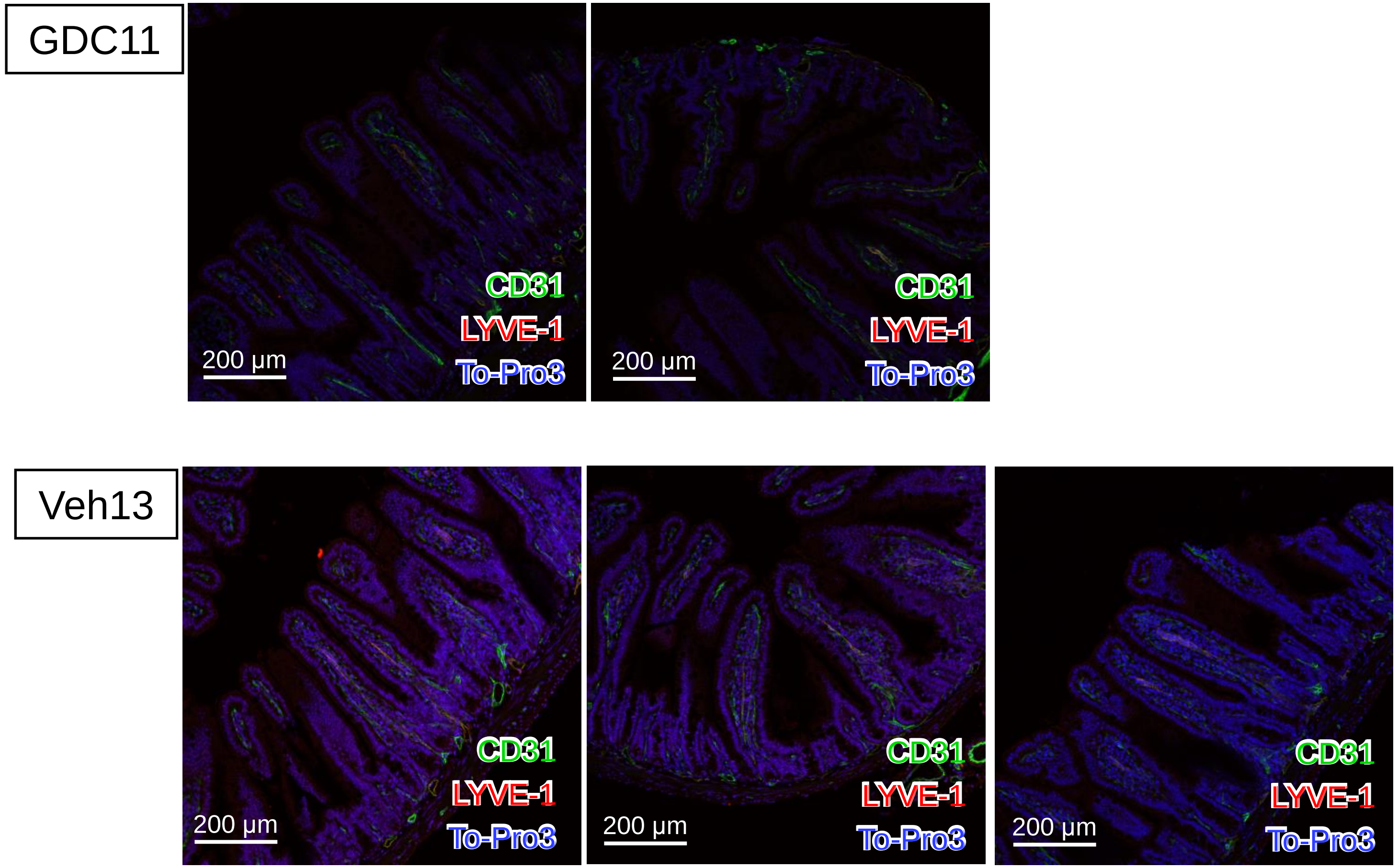

Supplement: Source Data Extended Data Fig. 4 — Micrographs for Extended Data Fig. 4. [file 42255_2023_828_MOESM21_ESM.pdf]
